# Highly Sensitive Detection of Individual HEAT and ARM Repeats with HHpred and COACH

**DOI:** 10.1371/journal.pone.0007148

**Published:** 2009-09-24

**Authors:** Fred Kippert, Dietlind L. Gerloff

**Affiliations:** 1 Edinburgh, Scotland; 2 Biomolecular Engineering Department, University of California Santa Cruz, Santa Cruz, California, United States of America; University of California, Riverside, United States of America

## Abstract

**Background:**

HEAT and ARM repeats occur in a large number of eukaryotic proteins. As these repeats are often highly diverged, the prediction of HEAT or ARM domains can be challenging. Except for the most clear-cut cases, identification at the individual repeat level is indispensable, in particular for determining domain boundaries. However, methods using single sequence queries do not have the sensitivity required to deal with more divergent repeats and, when applied to proteins with known structures, in some cases failed to detect a single repeat.

**Methodology and Principal Findings:**

Testing algorithms which use multiple sequence alignments as queries, we found two of them, HHpred and COACH, to detect HEAT and ARM repeats with greatly enhanced sensitivity. Calibration against experimentally determined structures suggests the use of three score classes with increasing confidence in the prediction, and prediction thresholds for each method. When we applied a new protocol using both HHpred and COACH to these structures, it detected 82% of HEAT repeats and 90% of ARM repeats, with the minimum for a given protein of 57% for HEAT repeats and 60% for ARM repeats. Application to *bona fide* HEAT and ARM proteins or domains indicated that similar numbers can be expected for the full complement of HEAT/ARM proteins. A systematic screen of the Protein Data Bank for false positive hits revealed their number to be low, in particular for ARM repeats. Double false positive hits for a given protein were rare for HEAT and not at all observed for ARM repeats. In combination with fold prediction and consistency checking (multiple sequence alignments, secondary structure prediction, and position analysis), repeat prediction with the new HHpred/COACH protocol dramatically improves prediction in the twilight zone of fold prediction methods, as well as the delineation of HEAT/ARM domain boundaries.

**Significance:**

A protocol is presented for the identification of individual HEAT or ARM repeats which is straightforward to implement. It provides high sensitivity at a low false positive rate and will therefore greatly enhance the accuracy of predictions of HEAT and ARM domains.

## Introduction

Internal tandem duplications have played an important role in protein evolution. Multiple duplications of segments 30 to 50 residues in length have been particularly successful as judged from their spread in the eukaryotic cell [1], [2]. The structures formed by these classical repeat families can be divided into closed structures with a fixed number of repeats in a clearly discernible domain (e.g. the β-propeller composed of WD40-type repeats), and open structures where the number of repeats can be highly variable from protein to protein and where insertions of non-repeat sequences are frequently observed. Most prevalent among the latter are α-helical multi-repeat arrays where subsequent repeats pack against each other around a common axis to form a continuous superhelix or solenoid. These include the Ankyrin, tetratrico peptide (TPR), as well as HEAT and ARM repeats [2].

**Figure 1:**
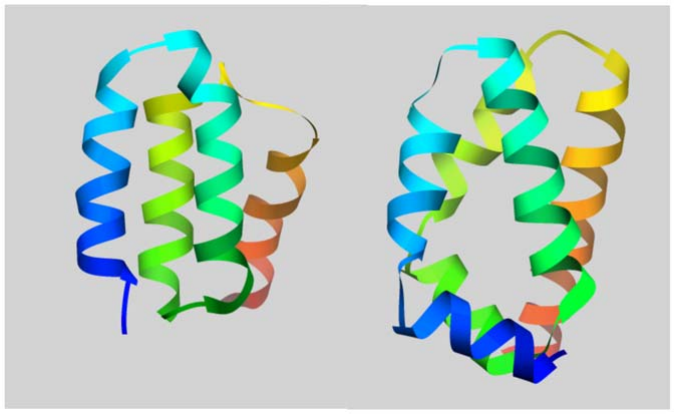
Archetypal HEAT and ARM repeats. The examples of repeat pairs shown correspond well to the described archetype. Left: HEAT (PDB∶1w63A), residues 376–499; right: ARM (PDB∶1ialA), residues 366–455. Images were rendered with USCF Chimera [33]; The structure are rainbow-coloured from N-terminus (blue) to C-terminus (orange).

HEAT and ARM repeats are structural units of typically two (HEAT) or three (ARM) α-helices which form one turn of a superhelix [3] (Fig. 1 shows examples that conform well with the established archetypes). Through hydrophobic interactions the helices of one repeat make contacts with their counterparts in pre- and succeeding repeats, thereby forming a continuous α-α-superhelix. Such tandem arrangements of repeats impose constraints on amino acid residue substitution that are characteristic for the repeat family [4]. Based on sequence, HEAT and ARM repeats can be distinguished from repeats found in other α-α-superhelix superfamilies such as TPR.

Sequence repeats belonging to the HEAT family were first observed in the regulatory subunit A of Protein phosphatase 2A [5]. Studying the Huntington's disease protein and using BLASTP, Andrade and Bork [6] noticed weak similarity between these two proteins. Further BLASTP as well as motif and profile searches revealed such repeats in 14 eukaryotic proteins and they were named HEAT after four of these proteins, *i.e. H*untingtin, *E*longation factor 3, regulatory subunit *A* of Protein phosphatase 2A, and *T*arget of rapamycin [6]. Andrade *et al.* [3] subsequently extended the list of eukaryotic HEAT repeat proteins and suggested that HEAT repeats could be clustered in three distinct classes: AAA which appears to comprise the majority of HEAT repeat proteins including the four name-giving ones; IMB specific for the Importin β family; and ADB specific for the Adaptins. A further collection of HEAT repeat proteins involved in chromosome-related functions was presented by Neuwald and Hirano [7].

**Figure 2:**
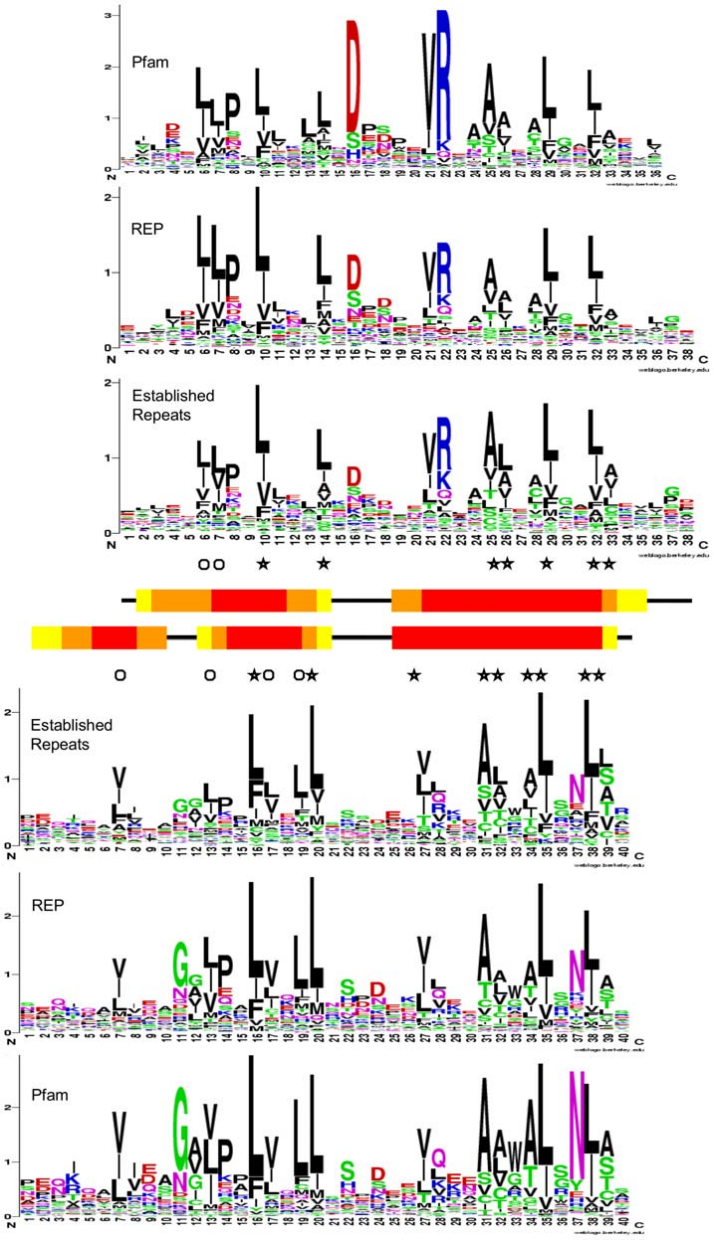
Sequence logos of HEAT and ARM repeats. The logos were generated with WebLogo [34] from the reference data sets described in Methods. Residues are shown in one letter code stacked in order of increasing frequency, with sizes proportional to their frequency at the position. The height of a column indicates the information content of the alignment at this position ranging from 0 if all amino acids are present at equal frequency to 4.32 ( = log_2_ 20) if there is no variation at the position. Asterisks mark positions where the frequency of hydrophilic residues (R, K, H, E, Q, D, N) is below 4%, circles mark additional positions were the frequency is between 4% and 10%. The consensus helices as indicated have been calculated from the information at the PDB web site and show positions where at least 90% (red), 70% (orange), or 50% (yellow) of repeats are annotated as α-helical.

ARM sequence repeats were first observed in yeast Importin α [8] and then in the *Drosophila* segment polarity protein Armadillo after which they were named [9]. Through iterative BLASTP searches, Peifer *et al.* [10] found similar repeats to be present in several unrelated eukaryotic proteins thereby indicating a repeat family. In the subsequent literature, this repeat family has variably, and largely interchangeably, been labelled Armadillo, Armadillo-like, or arm/Arm/ARM. Here we will use the term “ARM repeat” for the family and, in order to avoid confusion, will do so consistently also in cases where the cited literature used one of the other terms. When comparing the ARM repeats of Importin α with the HEAT repeats of Importin β, Malik *et al* [11] later realised that the originally suggested boundaries of ARM repeats [8], [9] did not correspond to those of the structural units. Once this was corrected, the relatedness between HEAT and ARM repeats became immediately apparent (see also Fig. 2), as has been further established by the comparative analysis of Andrade *et al* [3].

Within an α-α-superhelix the structural constraints are on the entire array rather than the individual repeat; a considerable degree of variability with respect to both sequence and structure can therefore be accommodated [2]. This “flexibility” has been suggested to form the basis of the evolutionary success of these repeats, allowing for rapid adaptation to different interaction partners [2]. At the sequence level this is reflected in the extent of sequence divergence that can be observed for individual repeats. This divergence makes identification of HEAT/ARM domains by fold prediction methodology far from trivial. First, there is the problem that some fold prediction programs tend to overpredict HEAT/ARM which has already led to a number of published mispredictions [12; a critical re-assessment of further published HEAT/ARM predictions will be presented elsewhere]. Second, in the case of multi-domain proteins, and many proteins with HEAT or ARM repeats belong to these, fold prediction programs are prone to extend alignments into adjacent non-HEAT/ARM regions if these are all α-helical and sometimes even if this is not the case. Because of these shortcomings, it is vital to extend any investigation to the individual repeat level.

The REP [4] and Pfam [13] servers are frequently used tools for the detection of single repeats. These methods match segments of single sequences to reference repeat profiles, or their derived Hidden Markov Models (HMM). Pfam uses standard HMMER2 [14] to search a library of template HMMs. REP is a specialist iterative algorithm where the detection of individual repeats is not independent of each other, taking into account that true repeat proteins will contain multiple copies. The significance thresholds applied by these two servers are stringent and one would expect them to detect only very typical repeats at a significant level. This expectation was confirmed by our benchmarking using proteins with known structures: many, and in some cases all, repeats of a protein remained undetected (see below).

Sensitivity can be increased when multiple sequence alignments rather than single sequences are used as queries. There are now several programs available that compare query profiles, or their derived HMMs, with a template HMM (for review see [15]), including HHpred [16] and COACH [17]. HHpred is a fast server for remote protein homology detection and structure prediction by comparing profile HMMs. COACH (Comparison of Alignments by Constructing Hidden Markov Models) aligns two multiple sequence alignments by constructing a profile HMM from one alignment and aligning the other to this HMM; it is freely available as a stand-alone version.

Over the past six years, the number of known structures of independent HEAT/ARM proteins deposited in the RCSB Protein Data Bank (PDB; [18]) has more than quadrupled. This allowed us to assemble meaningful reference alignments composed exclusively of established rather than predicted repeats. Using these data, we have developed a new protocol for the detection of HEAT and ARM repeats. In this paper we will i) calibrate HHpred and COACH and define confidence score ranges, ii) evaluate the sensitivity of our method with reference to all available HEAT/ARM structures, iii) investigate the occurrence of false positives, iv) apply the method to candidate proteins, and v) discuss potential limitations of the method when dealing with highly divergent family members.

## Results and Discussion

### New reference data sets composed exclusively of established repeats

**Figure 3:**
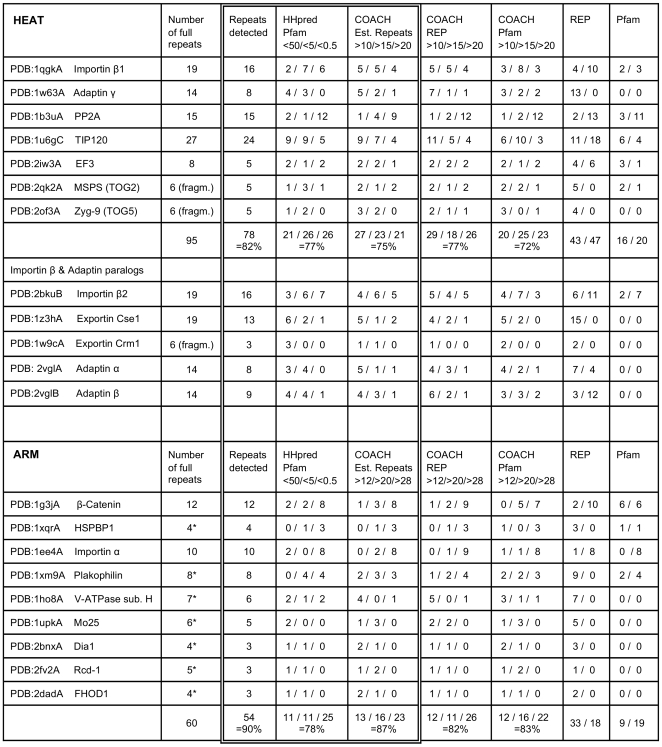
Detection of individual HEAT and ARM repeats in proteins with known structures. The number of full repeats was taken from the structures as deposited in PDB and/or associated publications; asterisks mark ARM proteins with truncated two-helix repeats at the N-terminus which were not included in the analysis. Repeats detected are those with matches better than the lowest threshold by HHpred (*i.e.* E-value <50) and/or COACH(Established Repeats; score >10 for HEAT and >12 for ARM). HHpred and COACH results were grouped in four classes as described in the text and the numbers of repeats falling in the three better scoring confidence classes are given here. REP and Pfam results are for subsignificant/significant matches as returned by the servers.

Since Andrade *et al.'s* systematic study [4], a considerable number of additional structures have been deposited in the PDB database for which an association with HEAT/ARM has been made by the authors. In order to build comprehensive reference data sets of eukaryotic HEAT and ARM repeats we looked at all structures where the link has been made by authors' statements and/or SCOP (Structural Classification of Proteins; [19]), or which were found by fold prediction using established HEAT/ARM proteins as queries. Details of all structures considered are given in Supporting Information S1. Only for a subset of these structures (listed in Fig. 3) was the classification as HEAT or ARM repeat proteins found to be valid.

We assembled multiple sequence alignments for each of these proteins as described in Methods. Only one paralog each was included of the Importin β and Adaptin families in order to avoid bias towards a particular class of HEAT repeats. The representatives of the final seven HEAT and nine ARM repeat protein families are specified in Fig. 3. The alignments were reduced to 15 orthologous sequences (seven sequences in case of metazoan-specific proteins) representing as wide a range of different taxa as possible. Segments covering individual repeats were selected according to secondary and tertiary structure information at the PDB [18] web site and/or associated publications, and were aligned to each other (note that we use “individual repeat” to refer to the structural entity in an alignment, while “single repeat” refers to the corresponding fragment in a single sequence). We excluded the very N-terminal and C-terminal repeats from the reference alignments because there are less structural constraints on these repeats, which frequently results in higher sequence divergence. The resulting alignments eventually comprised 1215 HEAT repeats and 575 ARM repeats, representing 81 and 49 individual repeats, respectively (for details see Methods). They will be referred to as “Established Repeat” alignments and are provided in Supporting Information S2. Fig. 2 shows that the sequence logos generated from these data are in substantial agreement with those based on the REP and Pfam reference data (see below).

### Characteristics of HEAT and ARM repeats - an update

**Figure 4:**
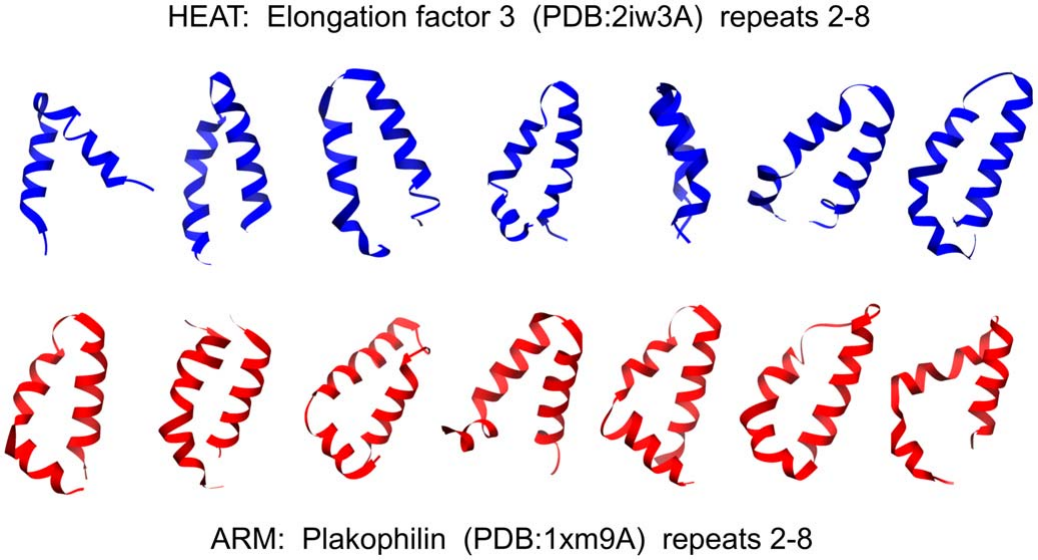
Structural variation amongst individual repeats of the same protein. Subsequent repeats of a HEAT (top, Elongation factor 3, PDB∶2iw3A, repeats 2–8) and an ARM (bottom, Plakophilin, PDB∶1xm9A, repeats 2–8) repeat protein are shown. Repeats are arranged such that the preceding repeat is approximately in the plane of the image with its central axis arranged vertically. Images were rendered with USCF Chimera [33].

A detailed look at the available structures gives a measure of the considerable variation between individual repeats and how much they can differ from the idealised picture painted by the repeat pairs shown in Fig. 1. To illustrate this, Fig. 4 shows seven consecutive repeats from the HEAT repeat protein Elongation factor 3 (PDB∶2iw3A) and the ARM repeat protein Plakophilin (PDB∶1xm9A). There is considerable variation in the length of the first helix in HEAT repeats, and in the length of the first and second helices in ARM repeats (see also Fig. 2). There is also substantive variation regarding the kink in the first helix in HEAT repeats, and the break between the first and second helices in ARM repeats. As Fig. 4 shows, this may cause some HEAT repeats to actually look like a typical ARM repeat, and *vice versa*. Furthermore, there are considerable differences in the various angles between consecutive repeats which determine how they pack to each other and thereby shape the overall geometry of the protein. In Fig. 4 the repeats have been arranged so as to emphasise the angle between the planes of subsequent repeats which defines the curvature at this point of the protein, and the angle between the central axes of subsequent repeats which defines the twist at this point of the protein (for a review of superhelix properties see [20]).

**Figure 5:**
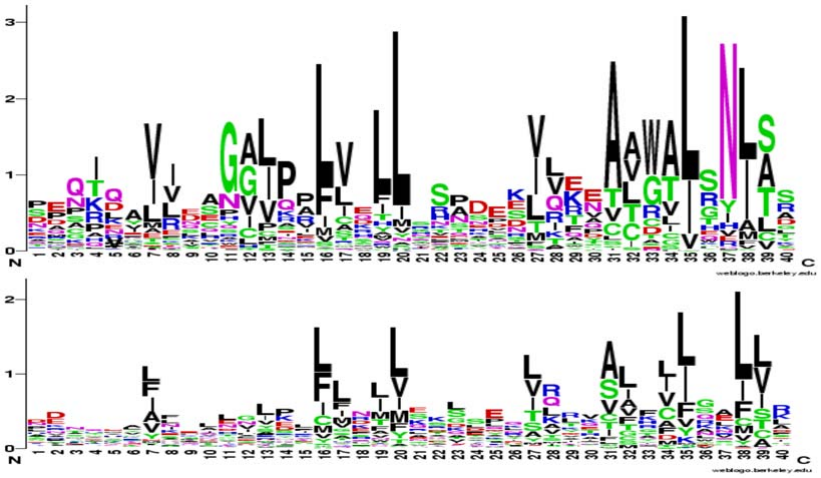
Sequence logos of typical and diverged ARM repeats. Comparison of “archetypal Armadillo” (top; PDB∶1g3jA, 1ee4A, 1xm9A) and more divergent ARM (bottom; PDB: 1xqrA, 1ho8A, 1upkA, 2bnxA, 2fv2A, 3dadA) repeats. The logos were generated with WebLogo [34] from the indicated subsets of the Established Repeats data, with details as in Fig. 2.

The observed variation in the structures of individual repeats is mirrored at the sequence level and it has been pointed out before that there are no characteristic sets of completely conserved amino acids that could be used as signature motifs [2]. In fact, a comparison of the Established Repeat alignments with the corresponding REP and Pfam data, as well as visual inspection of the alignments of individual structures, reveals that some positions previously believed to be characteristic of HEAT and ARM repeats do not show this preference in some proteins. In HEAT repeats, Pro8, Asp16 and Arg22 can be dramatically reduced. In ARM repeats, Gly11, Pro14, and Asn37 can be dramatically reduced or even completely absent. The latter is visualised in Fig. 5 which compares the profile of “archetypal Armadillo” repeats with that of the more divergent repeats (which are absent from the REP and Pfam reference alignments). Consequently, these positions are of limited diagnostic value in identifying more divergent HEAT/ARM repeats.

However, there are positions in the sequence alignments where there is a strong preference for the type of amino acid. At several positions in the alignment hydrophobic residues clearly dominate. Andrade *et al*. [2] have pointed out that the tandem arrangements of repeats impose constraints on amino acid substitution that are characteristic for each repeat family. Hydrophobic residues are found at specific positions at the buried face of the helices where they participate in intra- and inter-repeat packing, making up what has been called the “hydrophobic core” of the solenoid [3]. The availability now of substantive alignments composed exclusively of established repeats has enabled us to look at these positions more closely. Rather than looking at the prevalence of hydrophobic residues at a certain position, we found the absence of hydrophilic residues to be a more useful parameter. At seven positions in the HEAT alignment and nine positions in the ARM alignment (marked by asterisks in Fig. 2) we found the frequency of hydrophilic residues (D,E,H,K,N,Q,R) to be less than 4%; on average only 1.6% in HEAT and 1.3% in ARM. Amongst the 1215 repeats in the HEAT Established Repeats alignment, we found only five repeats with hydrophilic residues at more than one of these seven positions. Amongst the 575 ARM repeats, we found only five repeats with hydrophilic residues at more than one of these nine positions. Similar results were obtained for the REP and Pfam data. Closer inspection of the repeats in these alignments which did not conform revealed them to be either obvious misalignments or apparent false positives. Furthermore, the analysis of over 800 individual repeat units from *bona fide* HEAT or ARM proteins also showed strict agreement (data not shown). While we would not advocate the “constraints against hydrophilic substitutions” rule to be used as a diagnostic on its own, it serves as a valuable additional criterion for dealing with borderline cases (see below).

### Calibration and prediction thresholds for individual repeat detection with HHpred and COACH

Since there are no characteristic sets of completely conserved amino acids, identifying individual repeats within HEAT and ARM proteins based on their sequences can be a challenging task. Using single sequences as queries, expectations regarding sensitivity and specificity *a priori* have to be low when dealing with sequence segments as short as HEAT and ARM repeats. Submission of the sequences of the solved structures to the REP and Pfam servers confirmed these expectations. The overall detection rate was low, and for about half of the structures both servers failed to detect a single repeat at significant level (Fig. 3).

Both sensitivity and specificity can be dramatically improved by using multiple sequence alignments as queries. Various programs are publicly available that derive conservation/variation information from the aligned sequences of a known group (typically in the form of sequence profiles and/or HMMs) and then use it to search for related sequence regions. Recently, advanced methods have extended the profile strategy to considering multiple sequence information on both sides, target and template, to improve performance [15]. We tested methods which are readily available, as web servers or stand alone programs, and found HHpred [16] and COACH [17] to display the most promising performance.

**Figure 6:**
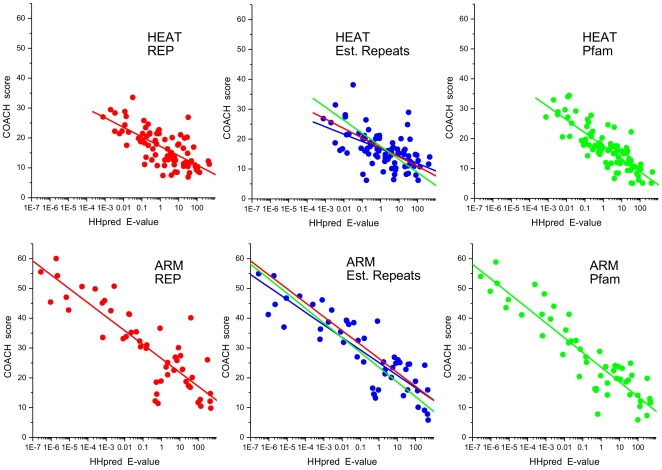
Correlation between HHpred E-values and COACH scores. HHpred and COACH results for identified repeats from the HEAT (top) and ARM (bottom) structures as specified in Fig. 3. Only repeats with HHpred E-values <500 and COACH scores >5 are included. The reference data sets for COACH analysis were: REP (left), Established Repeats (middle) and Pfam (right). Linear regressions are shown (all p<0.001); for comparison, the regressions for the REP (red) and Pfam (green) reference sets are also displayed in the middle panels.

For both programs calibration is necessary for them to be useful in routine applications. Because of the shortness of the segments and their high divergence, HHpred E-values for many repeat alignments will be much higher than one would find acceptable in full-length protein/domain structure prediction. The “SAM-style reverse scores” reported by COACH are not normalised. We therefore undertook a calibration using the Established Repeats alignments (here including the N-and C-terminal repeats). A total of 95 HEAT repeat and 60 ARM repeat segments were used for calibration. HHpred searches against the Pfam database, and COACH comparison with the REP, Pfam and Established Repeats reference data were carried out as described in Methods (for COACH analysis against the Established Repeats data the matching 15 sequences were removed from the reference alignment each time). Fig. 6 shows the COACH scores plotted against HHpred E-values of hits to PF02985 “HEAT repeat” (top row panels) and PF00514 “Armadillo/beta-catenin-like repeat” (bottom row panels), respectively. Correlation is apparent between the order of magnitude of the HHpred E-values and the COACH scores in all six plots.

Based on these data, prediction thresholds can be set. For HEAT repeats we found it useful to define four confidence classes corresponding to different degrees of “signal strength”, *i.e.* HHpred E-values ≥50, <50, <5, and <0.5, and COACH scores ≤10, >10, >15 and >20. The three higher score classes (HHpred E-values <50 and COACH scores >10), in which the reference repeats distributed approximately evenly (Fig. 3), indicate similarity to established HEAT profiles. Repeats falling into the respective score classes will be referred to as “detected” by the method. Dependent on which program was used, and in the case of COACH which reference data, detection rates ranged from 72% to 77% (Fig. 3).

The calibration for ARM repeats was somewhat complicated by the fact that 60% of the reference repeats came from the “archetypal Armadillo repeat” proteins that make up most of the Pfam and REP reference data. Unsurprisingly we found very low HHpred E-values and/or very high COACH scores for many of these repeats (Fig. 6) and when dealing with these proteins using a single threshold (instead of four classes as for HEAT) may seem sufficient. However, E-values and scores spread over a wider range for the more divergent ARM repeat proteins. Based on the analysis of additional candidate repeat fragments from *bona fide* ARM repeat proteins, we decided to adopt the same HHpred thresholds as for HEAT repeats, but slightly higher COACH thresholds, *i.e.* 12, 20 and 28. With these thresholds, detection rates ranged from 78% to 87% (Fig. 3).

As is evident from all six correlations shown Fig. 6, some repeats were detected by only one of the programs, either HHpred or COACH. This suggested that the results from both methods should be used in combination. If we consider a repeat detected if either its HHpred E-value or its COACH (Established Repeat reference alignment) score is better than the proposed thresholds, 82% of HEAT repeats and 90% of ARM repeats were detected (Fig. 3). This is clearly better than the rates observed for HHpred or COACH alone. Unsurprisingly many of the undetected repeats map to the very N- and C-termini of the proteins or domains. For the internal repeats, detection rates rose to 86% for HEAT and 95% for ARM.

### A screen of the Protein Data Bank reveals false positives to occur in low numbers

Our calibration results indicated that highly sensitive detection of HEAT and ARM repeat can be achieved with our suggested methods and thresholds. However, to be successful, predictions must also be highly accurate, *i.e*. overprediction must be kept at a minimum. In order to obtain a measure of the specificity of our protocol, a systematic screen for false positive repeat predictions was conceived. We used HHpred to search against the HMMs of all PDB entries, using the Established Repeats, REP and Pfam reference alignments as queries (for details see Methods). All hits to non-HEAT/non-ARM structures returned with E-values of up to 200 were then investigated according to our standard protocol. For HEAT we found 36 false positives hits in 31 proteins. For ARM we found only eight false positive hits in eight proteins. Five of the HEAT, but none of the ARM matches had scores in the two higher confidence classes. According to the latest version of SCOP there are 3464 protein families with known structures and, based on that number, false positives would occur in 1.4% (HEAT) and 0.4% (ARM) of families. Since the composition of the PDB database is somewhat biased, we also searched the Pfam and SMART databases (data not shown). The results suggest that false positive hit rates may be roughly double compared to what we calculated for the PDB; overall, 3% for HEAT and 1% for ARM seem realistic estimates.

We then tested what effect it would have on the false positive rate if we used less stringent thresholds in our detection protocol (which may allow detection of more true repeats). When we changed the prediction thresholds to E-values <200 for HHpred and scores >5 for COACH, false positive rates more than quadrupled. This was accompanied by only a modest increase in the detection rate with an additional six (of 95) HEAT and four (of 60) ARM repeats. Since the effect would be an undesirable shift towards overprediction, a lowering of the prediction thresholds was rejected.

Obviously one has to view false positive hits in the context of a whole HEAT/ARM domain or protein. We found only five proteins with two *bona fide* false positive hits against HEAT, and two further proteins with three such instances; no protein with more than one false positive ARM hit was observed. In contrast, we have not seen any established HEAT/ARM repeat proteins with less than two of their repeats scoring in the two higher confidence classes. Thus while a true HEAT or ARM repeat protein/domain can be expected to consist of several detectable repeats including some in the higher confidence classes, false positive hits in other proteins are generally isolated incidents occurring only at the lowest confidence level.

### Practical optimisation of a new protocol for HEAT and ARM repeat detection

**Figure 7:**
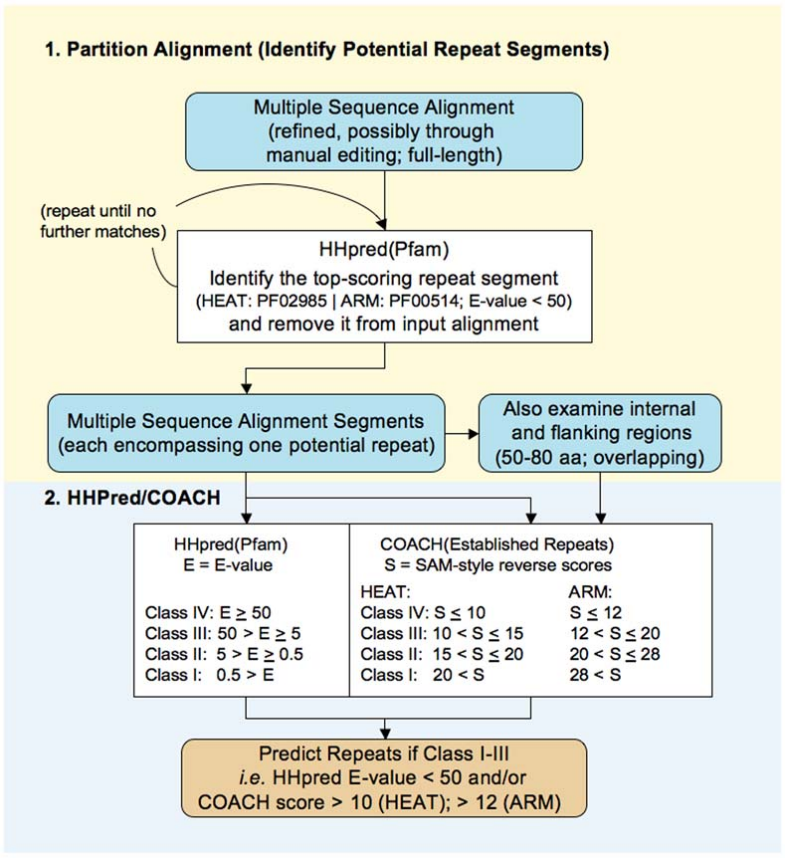
Protocol for repeat detection by HHpred and COACH. Flow chart of the protocol, for further details of the individual steps see Methods.

Based on the results of the calibration and false positive screen, we formulated a simple protocol for the detection of HEAT and ARM repeats in multiple sequence alignments (Fig. 7, see also Methods). With the usefulness of the prediction thresholds established it was important to optimise this new protocol with respect to practical considerations. Ideally, our detection protocol should i) detect individual repeats with near-optimal sensitivity in a few analysis steps, *i.e.* requiring only a small effort from the user, and ii) not be sensitive to variation in the query and reference alignments used as input. To this end we analysed in detail the scores obtained with different combinations of program, query alignments, and COACH reference alignments (the analysis of any such combination will be referred to as a “run” below).

As described above, combining the results from HHpred and COACH(Established Repeats) resulted in a noticeable increase in the detection rate. We also observed an increase through combining the methods when the REP and Pfam alignments were used as reference data instead. This strongly suggests hat both HHpred and COACH analysis should be performed routinely. The advantage became even more obvious when we looked at the detection rates for individual proteins, where the performance of the two programs may differ more substantially than overall. An example are the more divergent ARM repeat proteins where HHpred performs comparatively poorly (Fig. 3).

**Figure 8:**
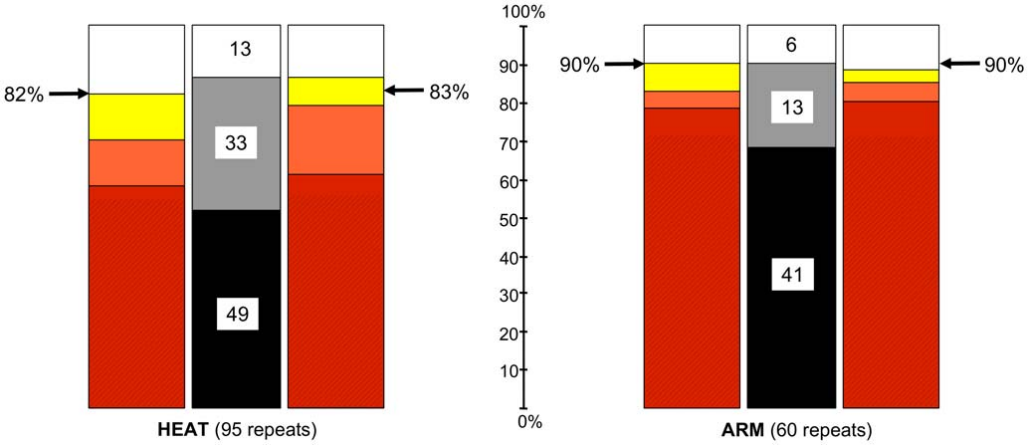
Repeat detection in dependence of reference and query alignments. The outer bars summarise the results of COACH runs with the three reference alignments, and different query alignments as described in the text (left-hand bars: query alignments as used in the calibration; right-hand bars, query alignments taken from the Established Repeat data, including N-and C-terminal repeats). Red: repeats detected with all three reference alignments (hatched area: repeats also detected by HHpred); orange: detected with two of the reference alignments; yellow: detected with one of the reference alignments; white: not detected with any of the reference alignments. Arrows mark the respective detection rates achieved with the protocol, which combines the results from the HHpred and the COACH (Established Repeats) runs. The inner bars summarise the results from all runs for each repeat type, including HHpred. Black: repeats detected in all eight runs; grey: repeats detected in one to seven runs; white: repeats not detected in any of the runs. The numbers given are how many repeats fall into each category.

HEAT repeat detection rates with COACH were very similar for the three sets of reference alignments (Established Repeats, REP and Pfam); each of the COACH runs detected between 72% and 77% of the 95 repeats (Fig. 3), with an average of 75%. However, the fraction of HEAT repeats detected with all of these data sets was only 58%, while the fraction detected with at least one of them was considerably higher at 82% (Fig. 8). Thus 24% of HEAT repeats were detected with only one, or two, of the reference alignments. This raised the question whether COACH analysis should routinely be performed with all three reference alignments. When we compared the detection rates achieved with the three possible combinations of HHpred and COACH, we found that when we combined the results from HHpred with those of the two COACH runs using the Established Repeat and REP reference alignments, this yielded only two more repeats than including only one of these COACH runs. No further repeats were detected by also including the results using the Pfam reference alignment.

Andrade *et al.* [3] reported that using separate reference data sets for the three suggested classes of HEAT repeats (AAA, IMB, and ADB) increased the detection rate with REP by about 50%. We therefore also considered the use of separate reference data in COACH analysis. However, using the corresponding class-specific subsets of the Established Repeat data did not result in the detection of any additional repeats. Moreover, neither pairwise comparisons of cumulative protein-specific repeat alignments through COACH, nor visually comparing the profiles derived from these alignments lent any support for the suggested partition.

For ARM repeats the fraction of repeats detected by COACH with all three reference alignments was higher with 78% (Fig. 8). Using the Established Repeats reference alignment gave the highest detection rate, due to better recognition of the more divergent ARM repeats. Including a COACH run with the REP reference alignments resulted in the detection of only one additional repeat and no further improvement was achieved using also the Pfam reference alignment.

In conclusion, combining HHpred and COACH(Established Repeats) results as specified in the protocol yielded the highest detection rates. Including an additional COACH run with the REP reference alignment may occasionally result in detection of an additional repeat, but for routine applications the extra effort seems disproportionate to the minor improvement. Our Established Repeats reference data also have the advantage that they can be updated once new structures become available in the PDB, which may eventually lead to a moderate further increase in detection rates, in particular for HEAT repeats.

To investigate the effect of different query alignments, we first repeated the analysis using query repeat alignments that had been further optimised. Query alignments taken from the Established Repeat data (here including the N- and C-terminal repeats) were different from those used in the calibration in that insertions between the helices had been removed and that their length was exactly the same as that of the reference alignments. Unsurprisingly, the average scores obtained with these query alignments were slightly better (data not shown). However, in terms of detection the overall results of this analysis were remarkably similar to those obtained in the calibration (outer bars in Fig. 8). Only three additional repeats became detectable by our protocol if these especially tailored alignments were used as input, indicating that further laborious refinement of our input alignments would not be worthwhile. As found for the calibration data, the combination of HHpred and COACH(Established Repeats) performed best overall with detection rates of 83% for HEAT repeats and 90% for ARM repeats.

From these two sets of runs with “high quality” query alignments a pattern emerged that we found consistently supported in our further analyses. Repeats can be grouped into three categories of detectability (middle bars in Fig. 8): those whose detection appears largely “fail-safe” *i.e.* which were reproducibly detected independently of program and input parameters; those that can be detected by some combinations of program, query alignment and reference alignment but not others; and a small fraction of repeats which remain undetectable by our methodology. For HEAT repeats, the 82% and 83% obtained with our protocol (arrows in Fig. 8) were close to the maximum that seems achievable with the methodology (86%). For ARM repeats, the maximum of 90% for the current set of repeats was matched.

While this analysis indicated that further improvement of the query alignments had only a negligible effect, using poorer alignments could impact more strongly on the detection rates. This is an important issue because the assembly of alignments as described (Methods) often required more time than the repeat analysis *per se*. For most of the proteins analysed here, the initial automated alignments (see Methods) were unsatisfactory over short segments and manual adjustments were indicated. However, in many of these instances the misalignment affected only part of a repeat, or a minor fraction of the sequences in the alignment, and the scores obtained with the raw alignment were often still better than prediction threshold (data not shown). Besides alignment quality the sequence composition of the multiple sequence alignment could potentially affect results. The non-redundant (nr) database at the National Center for Biotechnology Information (NCBI) is strongly biased towards sequences from just a few taxonomic groups. For the present study we produced better balanced alignments by selectively culling sequences from over-represented groups. To investigate the effect of biased query alignments, we subjected metazoan- and fungal-specific subsets to our protocol and found very little effect on the detection of repeats considered “fail-safe”.

For all sub-optimal alignments analysed in our study, the detection of the fail-safe repeats never dropped below 46 (out of 50) for HEAT repeats, and never below 39 (out of 41) for ARM repeats. Overall detection rates never dropped below 71% for HEAT and 82% for ARM, which would be perfectly adequate for many practical applications. Clearly users who spend additional effort on improving the different aspects of the input alignments will be able to achieve higher detection rates. In practice, how much effort each user will want to spend on such improvements will depend on the purpose of the prediction and the desired resolution. Notably, throughout our analyses the effect of sub-optimal alignments on specificity remained negligible.

### Application of the protocol to candidate HEAT and ARM repeat proteins

**Figure 9:**
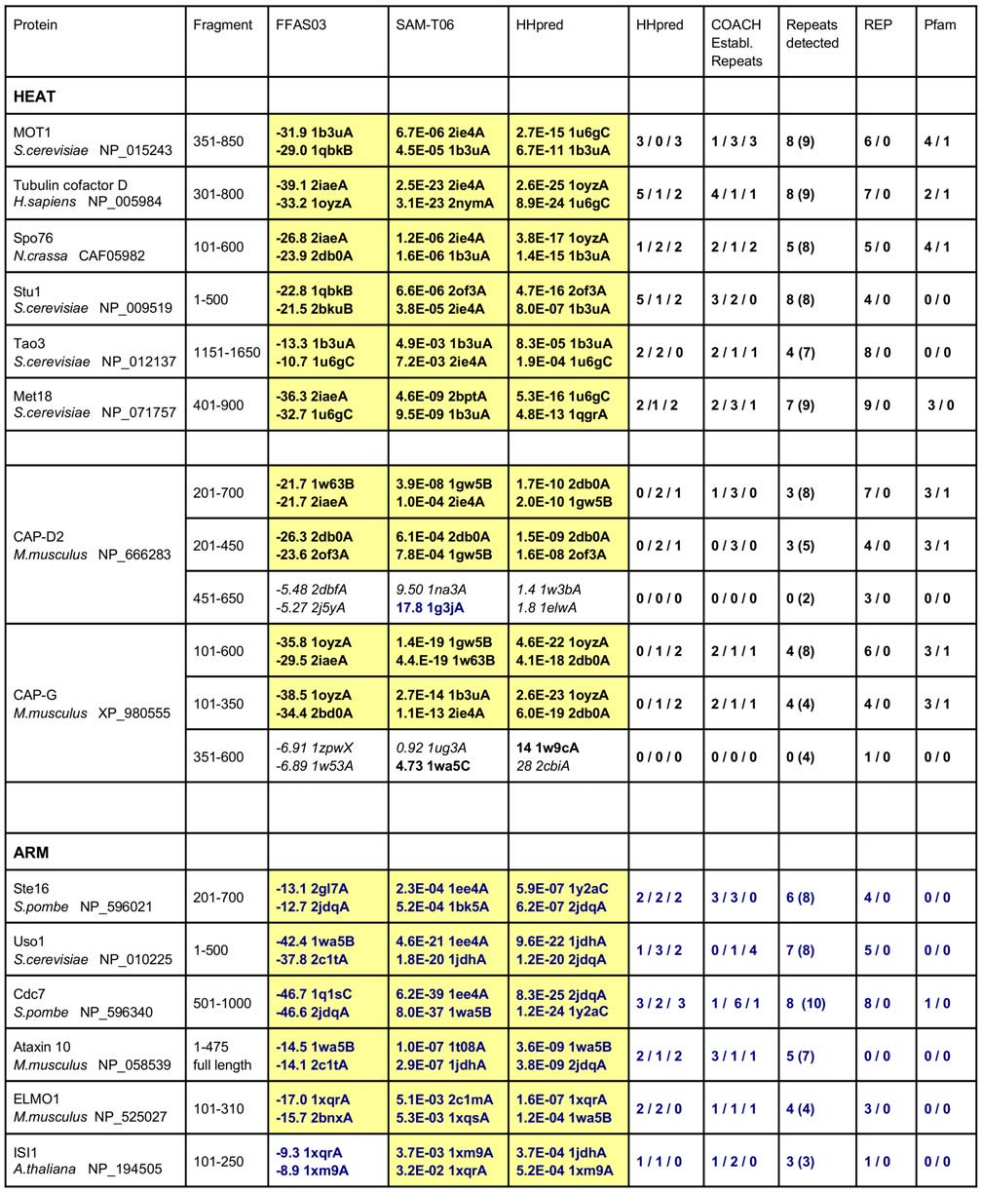
Fold prediction and individual repeat analysis of candidate proteins. The results of the application of fold prediction and individual repeat analysis to selected *bona fide* HEAT and ARM repeat proteins fragments are shown. The two top hits retrieved from fold prediction servers FFAS03 [25], SAM-T06 [24] and HHpred [16] are given. HEAT (both eukaryotic and prokaryotic) and ARM templates are shown in bold font, ARM templates in blue. Highlighted in yellow are matches with “significant” scores (see Methods). HHpred and COACH(Established Repeats) results were grouped in four classes as described in the text and the numbers of repeats falling in the three better scoring classes are shown here. Repeats detected are those with matches better than the lowest threshold by either HHpred or COACH(Established Repeats); given in brackets is the number of potential repeats, *i.e.* identified repeats plus additional helical segments of appropriate size. REP and Pfam results are for subsignificant/significant matches as returned by the servers.

To investigate how our protocol fares in routine predictive analysis, we applied HHpred and COACH to a large number of previously suggested candidate proteins/domains a selection of which is shown in Fig. 9. The HEAT repeat proteins are from Andrade *et al.* [3] or Neuwald and Hirano [7]. Three of the ARM repeat proteins are also from Andrade *et al.* [3]. Unfortunately all of the ARM proteins in that paper, as well as in the REP and Pfam reference data, belong to the group of “archetypal Armadillo” repeat proteins. To also look at more divergent repeat proteins we chose three proteins that were predicted to be ARM in recent years [21], [22], [23] and where multiple sequence alignments and consensus secondary structure predictions allowed us to confidently predict domain boundaries.

The first port of call for identifying HEAT/ARM proteins or domains will normally be fold prediction. As we will show elsewhere in a re-assessment of recently published HEAT/ARM predictions (FK & DLG, in preparation), some programs have a tendency to overpredict HEAT/ARM, sometimes dramatically so. We found profile-based fold prediction servers most reliable and routinely use HHpred [16], SAM-T06 [24] and FFAS03 [25]. Based on the servers' suggestions and our extensive control submissions we determined useful guideline thresholds (see Methods). Notably, these thresholds are only for proteins/fragments of up to 500 residues. We found scores to be length-dependent with all servers and even the most reliable ones tend to overpredict HEAT/ARM when queried with very long, largely α-helical proteins. On the other hand it has to be considered that fold prediction scores for shorter domains, frequently found in the case of ARM repeats, may struggle to surpass the thresholds, as the example of the three repeat domain in ISI1 shows (Fig. 9). A complication often found with HEAT repeat proteins is that their repeats may be rather widely spaced and in some proteins tend to occur in clusters [7]. Larger non-repeat insertion may result in comparatively poor scores as illustrated by the Tao3 example in Fig. 9. We also frequently observed considerable variation in the scores when we submitted homologous sequences from different taxonomic groups. Thus, except for the most clear-cut cases it is crucial to submit several orthologs and, where available, paralogs. If fold prediction results scatter around the suggested guideline thresholds, the protein in question should be treated as a twilight zone case (see below).

Fig. 9 indicates that our fold prediction methods of choice allow to distinguish between the two repeat types and we have observed that this applies generally. While there is “cross-detection” between HEAT and ARM (*i.e.* matches to the respective other repeat type still come up with scores better than prediction thresholds), they usually do so with much lower rankings than the correct type (data not shown). The equivalent situation is found at the single repeat level. We observed numerous matches better than threshold for the respective other repeat type but both the numbers of repeat matches in each protein/domain and their average scores were significantly reduced (data not shown).

As was the case for proteins with known structures, the individual repeat detection with HHpred and COACH proved to be highly successful with the HEAT and ARM candidate sets (Fig. 9). Our protocol detected 80% of the potential HEAT repeats and 83% of the ARM repeats. No efforts were made here to determine whether helical segments without match were in fact likely to be sub-threshold repeats; the fractions of undetected repeats may therefore be even lower. It is reassuring to note that the structure of the human ortholog of Uso1 has been solved [26] since we produced our prediction data; our repeat assignments were verified and only one repeat was missed by our protocol (data not shown).

Individual repeat detection is a valuable tool where fold prediction results are of borderline confidence, *i.e.* are insufficient to decide whether a protein is likely to have HEAT/ARM repeats or not. It is indispensable if one wishes to determine the boundaries of repeat domains or segments, and no published prediction should go without this. Individual repeat information cannot normally be garnered from fold prediction as all programs will, to varying degrees, tend to extend the query to template alignment substantially beyond the repeat segments, in particular if the adjacent regions are α-helical. Fig. 9 shows two examples (CAP-D2 and CAP-G) for this phenomenon. They are from Neuwald and Hirano [7] who reported the tendency of HEAT repeats to occur in clusters in many proteins. For these two examples, they found HEAT repeats only in the first half of the fragment in question (but further repeats more C-terminal). For the second half of the CAP-D2 fragment they observed compositional bias and we subsequently found this to be a small domain also occurring in unrelated proteins (FK & DLG, in preparation). By contrast, fold prediction servers we consulted yielded matches to HEAT templates over the full length when queried with the whole fragment. Only if the N- or C-terminal halves were submitted separately became the difference between them apparent (Fig. 9). The fold prediction results for the smaller fragments are in good agreement with those from HHpred/COACH analysis, indicating that combining the two methodologies is a good approach to at least considerably narrow down HEAT and ARM domain boundaries. Because of the higher divergence frequently observed in terminal repeats, a more detailed analysis of the flanking regions may still be necessary, unless the continuation of the HEAT/ARM can be ruled out for other reasons (e.g. incompatibility of predicted secondary structure).

### Limitations of HHpred/COACH repeat detection: application in the twilight zone

In light of the high sensitivity and high specificity demonstrated here one might expect that correct prediction of HEAT/ARM repeats and domains should be quite straightforward. However, as with any methodology, one must expect that some cases fall into a twilight zone. Here, true repeats may have diverged to such a degree that signals a very poor. Indeed, whichever program, query alignment and reference alignment we used, 13 HEAT repeats ( = 14%) and six ARM repeats ( = 10%) amongst the repeats from known structures remained undetected. These we consider to have diverged beyond recognition at the sequence level whilst having retained a HEAT/ARM repeat structure. If this applies to multiple repeats of a given protein, prediction can become problematic. On the other hand, in some proteins false positives may occur at a higher rate, possibly because these proteins share some common features with HEAT and ARM proteins (e.g. evolutionary unrelated α-α-superhelices).

Obviously a discussion of the twilight zone cannot be based on predictions but requires verified structures. Fortunately there are two structures which provide some insight into the issue. The first is that of the PP2A regulatory subunit B56 (PDB∶2iaeB, 2nnpA) which adopts an α-α-superhelix fold with eight closely spaced repeats, structurally not dissimilar to HEAT repeats. However, as has been acknowledged by the authors of the structure papers, there is no sequence similarity to HEAT repeats [27], [28]. Fold prediction results (hits ranked below the direct matches) had scores around our suggested guideline thresholds (see Methods): HHpred 9.0E-04, FFAS03 -10.8, SAM-T06 2.1E-02. On the other hand there is the structure of a fragment of the Exportin CRM1 (PDB∶1w9cA) comprising the six C-terminal HEAT repeats. Although it has been demonstrated that the Exportins are members of the Importin β family [29], their C-termini (six to eight repeats) have diverged to such a degree that PSI-BLAST searches using only the C-terminus do not find the Importin β paralogs (data not shown). Fold prediction results for the fragment encompassing the six repeats were also around the thresholds: HHpred 6.8E-03, FFAS03 -9.8, SAM-T06 2.1E-04 (but note that scores for more N-terminal fragments were highly significant). Looking at the results of the HHpred and COACH analysis for these two structures we find three matches in both cases. Thus these two proteins/fragments elicit very similar fold prediction scores and HHpred/COACH results, and can be considered to indicate a twilight zone where low scoring true hits and false positives overlap for both branches of our approach.

In cases where the combination of fold prediction and individual repeat analysis does not have sufficient diagnostic power to distinguish between true and false positives, further, more detailed analysis is required. Valuable information may come from an analysis of cumulative repeat alignments. For example, in the case of the Exportin fragment the cumulative alignment of the five internal repeat segments was “detected” by both HHpred (E-value 4.3) and COACH (score 11.2). Positional analysis revealed that none of the 75 CRM1 repeats in this cumulative alignment had more than one hydrophilic substitution at any of the seven core positions. In contrast, the cumulative alignment of the six B56 internal repeats scored very poorly (HHpred E-value 630, COACH score 1.3) and numerous repeats showed violations of the “constraints against hydrophilic substitutions” rule with two or three hydrophilic substitutions at any of the seven core positions. Our observations with B56 and CRM1 suggest that for HEAT repeats a number of cases will remain which cannot be satisfactorily resolved by fold prediction and standard individual repeat analysis with HHpred and COACH. These cases require further detailed analysis and expert judgement.

For ARM repeats we found the situation to be quite different. In a comprehensive survey of ARM repeat proteins in fungal proteomes (FK, unpublished observations) we found no indication that a similar twilight zone exists. We have not seen any example of a false positive candidate with more than one hit in HHpred/COACH analysis, and the rarely occurring hits by one of the two programs were always with scores in the lowest confidence class. Our studies indicated that the two proteins Mo25 and Rcd-1, whose structures are known, are probably among the most divergent ARM repeat proteins there are and we have not seen an example were the detection rate for internal repeats was below the 75% found for Rcd-1. Undoubtedly, future research will reveal some difficult cases also for ARM, but for the majority of cases ARM repeat prediction with our protocol will be highly robust and reliable.

## Methods

### Multiple sequence alignments and secondary structure prediction

The starting point of the new detection protocol are multiple sequence alignments which can be obtained as described in the following, or assembled with alternative methods preferred by the user. Homologous sequences of established and candidate HEAT/ARM proteins were identified through PSI-BLAST [30] searches (two iterations; default parameters) against the non-redundant (nr) protein sequence database at the NCBI using full-length sequences as queries. The number of iterations was restricted to two in order to avoid inclusion of spurious matches. Our sequence sets usually contained *bona fide* orthologs, but available paralogs were included where orthologs were restricted to a narrow taxonomic group and/or displayed high levels of identity. Each set of retrieved sequences was aligned with the implementation of MUSCLE [31] at the MPI Toolkit WWW-server [32]. Sequences with unusual insertions and/or deletions, stemming most likely from gene prediction errors, were removed, as were sequences that aligned very poorly (e.g. some protozoan parasite sequences). Because of the observed variation in fold prediction results (see below) we avoided, as a precautionary measure, bias toward any particular phylogenetic group(s). A balanced sequence set was obtained using HHfilter [32] to ‘cull’ the metazoan and fungal subsets of proteins. The resulting alignments for proteins occurring universally across the species range typically consisted of 20–30 sequences. Finally, adjustments were made manually where this obviously improved the initial automated alignment (this applied to the majority of proteins analysed). In the absence of manual refinement, repeats may remain undetected if they are particularly divergent between the different sequences, and poorly aligned by automated methods.

For secondary structure prediction alignments were submitted to Quick2D at the MPI Toolkit WWW-server [32] which returns predictions from four independent algorithms.

### Automated protein fold predictions

Full-length sequences or, in the case of larger proteins, sequence fragments of up to 500 residues of *bona fide* HEAT/ARM proteins were submitted to the three fold prediction servers HHpred [16], SAM-T06 [24] and FFAS03 [25]. When individual sequences were submitted to HHpred, global alignment mode and three PSI-BLAST iterations were selected; for alignment submissions the PSI-BLAST step was omitted. Corresponding fragments of several orthologous and, where available, paralogous proteins were also analysed to ascertain consistency as we frequently observed substantial variation in scores for sequences from different taxonomic groups. HHpred submissions were also made using the multiple sequence alignments as queries. Prediction scores as presented were collected October 2007 to February 2008 (results from a second set of submissions in January 2009 were fully consistent).

For the interpretation of results the following significance thresholds were used as guidelines. The FFAS03 server states “FFAS03 scores below −9.5 usually mean significant similarity (less than 3% of false positives)” and we adopted this threshold. SAM-T06 does not suggest a particular cut-off but rather states “E-values less than about 1.0E-5 are very good hits and are very likely to have a domain of the same fold as the target. E-values larger than about 0.1 are very speculative”. Based on our test submissions, we chose 1.0E-02 as threshold for SAM-T06 as we found little evidence for false positives with lower E-values. HHpred likewise does not suggest thresholds; results are given as E-values and probabilities and generally the authors suggest to use the latter. However, probabilities are less useful in the case of large all α-helical proteins (J. Söding, pers. commun.). We therefore used E-values and considered the ranking on the basis of the E-values, not probabilities. Based on the average correlation between probabilities and E-values in HHpred, as well as between the E-values of HHpred and SAM-T06 we found 1.0E-03 to be an appropriate threshold for HHpred.

### Reference data sets

REP reference data [4] were retrieved from the server's web site. The HEAT alignment contains 436 repeats, 427 of which are from *bona fide* eukaryotic HEAT repeat proteins. Two positions in the turn which were present in less than 11% of sequences were excised from the alignment. We noted that, for reasons unknown, the sixth alignment position is missing in the REP alignment; since there is little conservation at the N-terminus of the repeats, this should have only minimal effect on an analysis using the REP alignment. The Armadillo alignment contains 461 repeats, 353 of which are from *bona fide* ARM repeats, in the majority Importin α and β-Catenin. Of these repeats 54 are from *bona fide* HEAT proteins, mainly Importin β, and 47 are apparent false positives from three proteins (with distinct structures determined for all or part of these proteins). Three positions in the turns present in 6% to 15% of sequences were excised from the alignment.

Pfam reference data [13] were retrieved from the servers web site. The HEAT (PF02985) seed alignment contains 703 repeats, 646 of which are from *bona fide* eukaryotic HEAT proteins, with particular emphasis on Importin β and Protein phosphatase 2A. Eight positions present in less than 4% of sequences were excised from the alignment. Also excised were the three N-terminal positions as these are not included in the REP alignment and Pfam full alignments. The Armadillo/beta-catenin-like (PF00514) seed alignment contains 244 repeats, 240 of which are from *bona fide* ARM proteins. Eight positions, mostly in the turns, present in 2% to 31% of sequences were excised from the alignment. Also excised was the one N-terminal position which extends beyond the REP alignment.

For the Established Repeat reference data 15 representative orthologs (seven orthologs in case of metazoan-specific proteins) from different taxa were selected from the multiple sequence alignments described above. Fragments comprising full repeats were assigned according to PDB structures and associated publications. The fragments were aligned to each other strictly on a sequence basis. Insertions in the turns between the helices were removed and fragment length was restricted to 38 and 40 residues, respectively, to match the established repeat profiles [3]). The very N-terminal (except in the case of ARM proteins with a truncated repeat at the N-terminus with only two helices) and C-terminal repeats were removed. The resulting reference alignments include 1215 HEAT repeats and 575 ARM repeats, respectively (provided in Supporting Information S2).

### Protocol for individual repeat detection with HHpred and COACH (Fig. 7)

First, candidate fragments spanning individual repeat units are identified through an iterative procedure using HHpred [16] as follows. The full-length multiple sequence alignment is used in a search against the Pfam [13] database. If a match to PF02985 (“HEAT repeat”) or PF00514 (“Armadillo/beta-catenin-like repeat”) is found, the corresponding fragment in the multiple sequence alignment is considered a potential repeat fragment. It is deleted from the alignment and the remainder is input to another HHpred search. This is continued until no further match is found.

All thus identified candidate fragments are then individually submitted to HHpred and COACH. COACH analysis is performed with template HMMs derived from the Established Repeats alignments (Supporting Information S2) and the returned “SAM-style reverse scores” are recorded. Regions outside these candidate segments, and fragments without HHpred match in the initial searches, are investigated analogously, in overlapping fragments of 50–80 residues, taking into account the secondary structure predictions. A repeat is considered detected if the HHpred E-value is lower than 50 or the COACH score exceeds 10 (12 for ARM repeats).

### HHpred and COACH program availability and application

HHpred is a web server available at http://toolkit.tuebingen.mpg.de/hhpred which was used in this study. Recently it has also become available at http://toolkit.lmb.uni-muenchen.de/hhpred. HHpred is based on HHsearch for which new versions are released regularly. All results presented here were based on version 1.5 but preliminary results obtained with version 1.4 as well as results obtained with version 1.6 (which is already implemented at the new server site) have all been consistent throughout. It is conceivable that future development of HHpred may affect E-values and it is therefore advisable that users check whether the results obtained with future versions are still consistent with the calibration presented here. If necessary a re-calibration can be performed using the Established Repeat reference data provided in Supporting Information S2. These repeat alignments can also be used to test and compare novel profile- and HMM-based methods if and when they become available.

For individual repeat alignments HHpred submissions were made without an additional PSI-BLAST step and in global alignment mode. To detect potential matches irrespectively of their statistical significance, which is generally low in searches with such short query sequences, the minimum probability cut-off was set to 1%, and the maximum number of hits extended to 500. HHpred returns both E-values and probabilities but we found only E-values useful for this application.

COACH can be downloaded as part of the Lobster suite of programs from http://www.drive5.com/lobster (as Windows and Linux binaries, and also as source code; a tutorial is available from the same site) and is easy to install. As a small, single-task program with few input options it is straightforward to use also for non-experts. According to the program's developer no new versions are planned and the current version will be available from this site in the long term (R. Edgar, pers. commun.). There are no alternative parameter options in COACH, but it is necessary to add “-rev” to the command line in order to also obtain “SAM-style reverse scores” (the Viterbi scores delivered by default are not suitable for this application).

For HEAT and ARM candidate proteins, the protocol was applied exactly as described above. In the case of the HEAT and ARM proteins whose structures had been determined (Fig. 3), repeat fragments as indicated in PDB and/or the associated publications were subjected to HHpred and COACH analysis. For benchmarking, individual sequences were also submitted to the REP [4] and Pfam [13] servers to determine matches to HEAT and Armadillo profiles at significant and subsignificant levels as specified by the servers.

### False positive screen of PDB

The RCSB Protein Data Bank [18] was searched with HHpred using the Established Repeat, REP and Pfam reference data. All hits to structures other than established HEAT/ARM with E-values of up to 200 were investigated further. The identified sequences were used as queries in HHpred searches against Pfam (two PSI-BLAST iterations, otherwise as above). For the analysis of candidate segments, the HHpred query alignments, rather than custom alignments were used. Only if the alignment fragment that had elicited a hit in the screen was found to be detected applying our standard protocol was the remainder subjected to the iterative procedure as described above. The numbers of false positive hits were calculated according to the following criteria: i) hits for a particular fragment were counted only once per protein family; ii) if for a particular fragment hits were found for both HEAT and ARM, only the hit with the better score was counted as the true or false positive. iii) hits to *bona fide* prokaryotic-type HEAT repeat proteins (see Supporting Information S1) were in this context considered true positives.

## Supporting Information

Supporting Information S1 HEAT/ARM-repeat structures in the Protein Data Bank
(0.23 MB PDF)

Supporting Information S2 Established Repeat reference alignments
(0.92 MB PDF)
