# Supplementary material for: Highly Sensitive Detection of Individual HEAT and ARM Repeats with HHpred and COACH
Source: PLoS One. 2009 Sep 24;4(9):e7148. doi: 10.1371/journal.pone.0007148 (PMC2744927; doi:10.1371/journal.pone.0007148)
Supplement: Supporting Information S1 — HEAT/ARM-repeat structures in the Protein Data Bank (0.23 MB PDF) [file pone.0007148.s001.pdf]

Supporting Information S1 - HEAT/ARM-repeat structures in the Protein Data Bank

The primary resource for classification of structures deposited in the Protein Data Bank (PDB) [1] is the Structural Classification of Proteins (SCOP) database [2] and *a priori* all domains assigned to the superfamily “ARM repeat” in the 1.73 (November 2007) release and the pre-SCOP database (as of December 2008) release were considered to be HEAT/ARM candidate domains. In order to identify domains that have not yet been included in SCOP, or may have been misclassified, we performed two searches of the PDB database. First, we used the PDB keyword search to find all domains where authors have associated structures in some way with HEAT/ARM. Second, we submitted established HEAT and ARM domains to fold prediction (HHpred, FFAS03, SAM-T06) to find matches to additional structures with E-values / scores of at least around our ‘indicative’ thresholds. We considered all thus retrieved domains also to be HEAT/ARM candidates (listed in Table ST1). They were initially subjected to fold prediction to determine whether they could be matched, at significant level, to established HEAT/ ARM domains. Once our protocol for individual repeat detection had been developed, these domains were also submitted to individual repeat analysis by HHpred and COACH. In total 21 eukaryotic domains were identified as true HEAT or ARM (Table ST1.A and Fig.3 in main text) and the repeats from seven HEAT (only one member each of the importin β and adaptin families) and nine ARM repeat domains were finally included in the reference data set.

Omitted from the reference data were the members of the ENTH/VHS domain family (Table ST1.B). These are currently classified in their own SCOP superfamily, but have repeatedly been linked to HEAT/ ARM. They normally have three repeats, though some may have C-terminal extensions. They have been described, rather curiously, by some authors as having two HEAT and one ARM repeat, but others have not made any association beyond ENTH/VHS. In our individual repeat detection, the middle repeat attracted a significant match to HEAT in all ENTH/VHS structures, in some cases with very high scores. In some VHS domains there was also a significant HEAT match for the third repeat. This suggests that ENTH/VHS domains may well be evolutionary related to HEAT, but because of the shortness of the domain, any association must remain at a tentative level. We have excluded ENTH/VHS domains from the HEAT reference data primarily for two more practical reasons. First, because these domains are small and have a fixed number of repeats, identification is better approached at the whole domain rather than individual repeat level. Second, VHS/ENTH domains would have contributed only a single internal repeat to the HEAT reference alignment which would not have had any discernible effect.

Our reference data sets consist exclusively of repeats from eukaryotic proteins and proteins with prokaryotic HEAT(-like) repeats (Table ST1.C) were deliberately omitted. The HEAT-like repeats found in prokaryotes show some similar characteristics, as is apparent from their Pfam (PF03130; “PBS lyase HEAT-like repeat”) profile as well as from the structures currently in PDB. However, closer analysis of profile and structures showed that both helices of a repeat are, on average, a full turn shorter at their respective inter-repeat sides (data not shown). If eukaryotic HEAT repeats had evolved from prokaryotic ones, duplication of a single, lengthened repeat would appear the most likely scenario; in which case the duplication of an evolutionary unrelated α-hairpin would seem as likely a conjecture. In practical terms of repeat detection it is important to note that our searches have indicated that prokaryotic-type HEAT repeats are very rare in eukaryotes. In only a single case is the association obvious because of the strongly repetitive sequence [3]. One of the three further candidates we have found so far is lipovitellin (PDB:1lshA), currently in its own SCOP superfamily “Lipovitellin-phosvitin complex”. However, a more

thorough characterisation of prokaryotic-type repeats would be needed before any association should be made witch confidence. In practical terms it is important to note that in fold prediction there is a strong cross-reactivity between eukaryotic and the longer prokaryotic proteins, in particular 1oyzA and 2b0A (several examples in Fig.9 in the main text).

Notably we have excluded eight eukaryotic protein families even though they have been assigned to the SCOP superfamily “ARM repeat” (Table ST1.D). According to the SCOP classification criteria, for inclusion in a superfamily, proteins should have a “probable common evolutionary origin”, which means that “proteins that have low sequence identities, but whose structural and functional features suggest that a common evolutionary origin is probable are placed together in superfamilies”. In this respect, the SCOP fold “alpha-alpha superhelix” is a somewhat special case as most of its superfamilies share structural and functional features even though they are not evolutionary related. This suggests that assignment to a particular superfamily within this fold is sensible only if based on detectable sequence similarity. We found this to apply only to the proteins given in Table ST1.A, but not to the other families of eukaryotic-specific proteins within the “ARM repeat” superfamily (using PSI-BLAST, fold prediction and analysis at the individual repeat level). We must assume that in most cases the classification has been based solely on authors’ statements in the describing publications. From this literature it is apparent that some authors may simply have confused HEAT repeats with pairs of anti-parallel α-helices in general [4]. In other cases, the terminology used already indicates that the association should not be made at the superfamily level, *e.g.* for the “pseudo-HEAT repeat analogous topology domain” [5] or the “HEAT analogous motif “ [6] (see also Table ST1.C). In many of these cases the authors’ statements have been based on the interpretation of structure comparisons using the DALI server [7]. That structural similarity does not necessarily reflect sequence similarity has been implicitly acknowledged by some authors when using terms such as “resembles”, “structural resemblance”, similar in its topology”, or “fold akin to”. It is then very unfortunate that such cautious statements have the tendency to become transformed, either in the same paper on in subsequent literature, such that an evolutionary and therefore sequence-based association with HEAT/ARM is made. Fortunately the mis-classifications seem to have little impact in practical terms: neither in the present study, nor in further analyses, have we ever observed matches to these proteins at a significant level, with the notable exception of the B56 regulator subunit of PP2A (for a discussion of this “twilight zone case” see main text).

Table ST1. **Structures linked to HEAT/ARM**. Structures in the Protein Data Bank that have been linked with HEAT/ARM through SCOP classification, authors' statements, or fold prediction searches. SCOP classifications are given where available; SF = superfamily, F = Family. Authors' statements were taken from the describing publications, references given at the PDB site. Results from fold prediction with FFAS03, HHpred, and SAM-T06 were grouped in four categories: “++”, highly significant matches at >99% confidence level; “+”, matches around or above our selected thresholds, largely consistent between the three servers; “(+)”, some matches below the thresholds, often with differences between the three servers; “-”, no matches. RD: repeats detected versus structural repeats. [a] only one example each given for ENTH/VHS families; [b] LRV is composed of short 3_{10} and alpha-helical repeats, potentially related to prokaryotic HEAT repeats; [c] confidently predicted to be composed of tetratrico peptide (TPR) repeats; [d] contains only a single alpha hairpin; [e] for a further discussion of the B56 family see main text.

SCOP classification	Protein / PDB code	Author's statement	FP	RD
**A. Established HEAT or ARM repeat proteins**				
SF ARM repeat<br>F Armadillo	β-catenin<br>1g3jA	Established HEAT/ARM proteins	++	12/12
SF ARM repeat<br>F Armadillo	Importin α<br>1ee4A		++	10/10
SF ARM repeat<br>F Armadillo	Importin β1<br>1gqkA		++	16/19
SF ARM repeat<br>F Armadillo	Importin β2<br>2bkuB		++	16/19
SF ARM repeat<br>F Armadillo	Exportin Cse1<br>1wa5C		++	13/20
SF ARM repeat<br>F Clathrin adaptor core protein	Adaptin α C<br>2vglA		++	8/14
SF ARM repeat<br>F Clathrin adaptor core protein	Adaptin β<br>2vglB		++	9/14
Class Low resolution protein structures<br>F Clathrin assemblies	Adaptin γ1<br>1w63A		++	8/14
SF ARM repeat<br>F HEAT repeat	PP2A regulatory subunit α<br>1b3uA		++	15/15
SF ARM repeat<br>F HEAT repeat	Cand1/Tip120<br>1u6gC		++	24/27
SF ARM repeat<br>F Mo25 protein	Mo25<br>1upkA		++	5/6
SF ARM<br>F V-type ATPase Regulatory subunit H	V-type ATPase regulatory subunit H<br>1ho8		++	6/7
SF ARM repeat<br>F Exportin HEAT-like repeat	Exportin Crm1, six repeat fragment<br>1w9cA		+	3/6
SF ARM repeat<br>F HspBP1 domain	HspBP1<br>1xqrA		++	4/4
SF ARM repeat<br>F Diap1 N-terminal region-like	Dia1<br>2bnxA		++	3/4
SF Arm repeat<br>F Plakophilin 1 helical region	Plakophilin 1<br>1xm9A		++	8/8
-	Stu2 6 repeat TOG cassette<br>2qgk1		++	5/6
-	Zyg-9 6 repeat TOG cassette<br>2of3A		++	5/6
-	Elongation factor 3<br>2iw3A		++	5/8
-	Rcd-1<br>2fv2		+	3/5
-	FHOD1<br>3dadA		+	3/4
**B. ENTH/VHS-like domains, potentially HEAT-related**				
SF ENTH/VHS domain<br>F ENTH domain	Epsin 1<br>1eduA	described as having two HEAT repeats and one ARM repeat by some authors, while others did not suggest any association beyond ENTH/VHS	-	1/3
SF ENTH/VHS domain<br>F VHS domain [a]	HRS<br>1dvpA		(+)	2/3
SF ENTH/VHS domain<br>F Phosphoinosit.-binding clathrin adaptor[a]	AP180<br>1hx8A		-	1/3
SF ENTH/VHS domain<br>F RPR domain	PCF11<br>1szaA		(+)	1/3

SCOP classification	Protein / PDB code	Author's statement	FP	RD
**C. Proteins with prokaryotic-type HEAT repeats**				
SF ARM repeat<br>S Leucine-rich repeat variant [b]	Leucine-rich repeat variant<br>1lrvA	novel repetitive protein structural motif	na	na
SF ARM repeat<br>F PBS lyase HEAT-like repeat	Hypothetical protein YibA<br>1oyzA	unpublished	na	na
SF ARM repeat<br>F PBS lyase HEAT-like repeat	MTH187<br>1te4A	HEAT-like repeats	na	na
SF ARM repeat<br>F BC3264-like	Hypothetical protein BC3264<br>1t06A	unpublished	na	na
SF ARM repeat<br>F BC3264-like	Hypothetical protein EF3068<br>2b6cA	unpublished	na	na
SF ARM repeat<br>F BC3264-like	DNA glycosylase AlkD<br>3bvsA	HEAT-like repeats	na	na
SF ARM repeat<br>F GUN4-associated domain	GUN4-like protein Ycf53<br>1z3yA	resembles ARM or HEAT repeat	na	na
SF Aconitase B, N-terminal domain<br>F Aconitase B, N-terminal domain	E.coli Aconitase B<br>1l5jA	HEAT-like domain	na	na
SF Lipovitellin-phosvitin complex<br>S Lipovitellin-phosvitin complex	Lipovitellin<br>1lshA	no association suggested	na	na
-	P0542<br>2db0A	unpublished	na	na
**D. No or insufficient relationship to HEAT or ARM by fold prediction and individual repeat detection**				
SF ARM repeat<br>F MIF4G domain-like	Eukaryotic initiation factor eIF4G<br>1hu3A	arranged as five antiparallel a helical pairs or HEAT repeats	-	0/5
SF ARM repeat<br>F MIF4G domain-like	Eukaryotic initiation factor eIF4G<br>1ug3A (different MA3 domains)	atypical HEAT domains	-	2/10
SF ARM repeat<br>F MIF4G domain-like	eIF-2b epsilon<br>1paqA	organized in a manner similar to HEAT repeats	-	0/5
SF ARM repeat<br>F MIF4G domain-like	UPF2<br>1uw4A	no association suggested	-	0/5
SF ARM repeat<br>F MIF4G domain-like	CBP80<br>1h6kA	do not have a recognizable signature	(+)	2/15
SF ARM repeat<br>F MIF4G domain-like	PDCD4<br>2nszA	analogous to HEAT domains	-	0/5
SF Arm repeat<br>F Phat domain	Smaug<br>1oxjA	pseudo-HEAT repeat analogous topology domain	-	0/2
SF ARM repeat<br>F Leukotriene A4 hydrol. C-terminal	Leukotriene A4 hydrolase<br>1hs6A	resembles armadillo repeats or HEAT motif regions	-	0/4
SF ARM repeat<br>F Clathrin heavy chain proximal leg [c]	Clathrin heavy chain<br>1b89A	resembles a tetratrico peptide repeat	TPR	0/10
SF ARM repeat<br>F Clathrin heavy chain linker domain [d]	Clathrin heavy chain<br>1utcA	no association suggested	-	0/1
SF ARM repeat<br>F Phosphoinosit. 3-kinase helical domain	Phosphoinositide 3-kinase<br>1e7uA	fold akin to HEAT repeat containing structures	(+)	1/5
SF ARM repeat<br>F Pumilio repeat	Pumilio 1<br>1m8zA	similar to the topology of Armadillo (ARM) repeats	-	0/8
SF ARM repeat<br>F eIF3k, N-terminal domain	eIF3k<br>1rz4A	HEAT repeat-like HAM (HEAT analogous motif) domain	-	0/3
SF ARM repeat<br>F B56-like [e]	PP2A regulat. subunit B56-gamma<br>2nppB / 2iaeB	HEAT-like repeat motifs / pseudo-HEAT repeats	+	3/8
SF TROVE domain-like<br>F TROVE domain-like	Ro Autoantigen<br>1yvrA	composed of HEAT repeats	-	0/~9
SF IP3 receptor type 1 binding core<br>F IP3 receptor type 1 binding core	Inositol 1,4,5-trisphosph. receptor<br>1n4kA	‘armadillo repeat’-like fold	-	0/3
-	FancF<br>2iqcA	structural resemblance to HEAT repeat motifs	-	0/5

References

1 Berman HM, Westbrook J, Feng Z, Gilliland G, Bhat TN et al. (2000) The Protein Data Bank. Nucleic Acids Res 28: 235-242.

2 Murzin AG, Brenner SE, Hubbard T, Chothia C (1995) SCOP: a structural classification of proteins database for the investigation of sequences and structures. J Mol Biol 247: 536-540.

3 Park JH, Aravind L, Wolff EC, Kaevel J, Kim YS et al (2006) Molecular cloning, expression, and structural prediction of deoxyhypusine hydroxylase: a HEAT-repeat-containing metalloenzyme. Proc Natl Acad Sci U S A 103: 51-56.

4 Marcotrigiano J, Lomakin IB, Sonenberg N, Pestova TV, Hellen CU et al. (2001) A conserved HEAT domain within eIF4G directs assembly of the translation initiation machinery. Mol Cell 7: 193-203.

5 Green JB, Gardner CD, Wharton RP, Aggarwal AK (2003) RNA recognition via the SAM domain of Smaug. Mol Cell 11:1537-1148.

6 Wei Z, Zhang P, Zhou Z, Cheng Z, Wan M (2004) Crystal structure of human eIF3k, the first structure of eIF3 subunits. J Biol Chem 279: 34983-34990.

7 Holm L, Park J. (2000) DaliLite workbench for protein structure comparison. Bioinformatics 16: 566-567.
