# Supplementary material for: Highly Sensitive Detection of Individual HEAT and ARM Repeats with HHpred and COACH
Source: PLoS One. 2009 Sep 24;4(9):e7148. doi: 10.1371/journal.pone.0007148 (PMC2744927; doi:10.1371/journal.pone.0007148)
Supplement: Supporting Information S2 — Established Repeat reference alignments (0.92 MB PDF) [file pone.0007148.s002.pdf]

Supporting Information S2 - Established Repeat reference alignments

Established Repeat alignments as used as reference data in the COACH analyses.

For each individual repeat alignment the HHpred E-values as obtained in the current study is given to facilitate recalibration with new versions of HHpred in the future, if necessary.

<u>HEAT repeat reference alignments</u> **current HHpred E-values**

```
>pp2a_2_M.musculus                              2.7
RTRSELLPFLTDTIYDED-EVLLALAEQLGTFTTLVGG
>pp2a_2_S.cerevisiae
RTRNELIPFLTEVAQDDEDEVFAVLAEQLGKFVPYIGG
>pp2a_2_O.luminarius
RARSELVPFLAERDDEDD-ECLLAIAGELATLIDAVGG
>pp2a_2_Y.lipolytica
RCRDELIPFLEDVTQEDEDEVLTVLAEELANLVPYIGG
>pp2a_2_S.pombe
RTRDELIPFLDESIDDED-EVLSALADQLGNFVDYVGG
>pp2a_2_A.niger
RTREELIPFLDDSVEDED-EVLTALSEELGNFIEYVGG
>pp2a_2_U.maydis
RARDELIPFLQDSLDDED-EVLLALAQELGSFVEYLGG
>pp2a_2_D.discoideum
RTRTELIPYLQDSVLEDEDEVLVVLSEELGNLIEFVGG
>pp2a_2_A.thaliana
RTRKELIPFLSENSDDDD-EVLLAMAEELGVFIPFVGG
>pp2a_2_O.sativa
RTRKELIPFLSENNDDED-EVLLAMAEELGVFIPYVGG
>pp2a_2_C.elegans
RTRNELIQFLTDTIYDED-EVLLVLAEQLGNFTPLVGG
>pp2a_2_D.melanogaster
RTRSELIPFLTETIYDED-EVLLALADQLGNFTSLVGG
>pp2a_2_X.tropicalis
RTRSELLPFLTDTIYDED-EVLLALAEQLGTFTSLVGG
>pp2a_2_D.rerio
RTRTELLPFLTDTIYDED-EVLLALAEQLGSFTVLVGG
>pp2a_2_A.mellifera
RTRSELIPFLTESMYDED-EVLLALAEQLGQFTPLVGG
>pp2a_3_M.musculus                              0.11
EYVHCLLPPLESLATVEETVVRDKAVESLRAISHEHSP
>pp2a_3_S.cerevisiae
QYATILLPVLEILASAEETLVREKAVDSLNNVAQELSQ
>pp2a_3_O.luminarius
EHVHVILAPLETLITVEETVVRAKAVETACAVGRAMTP
>pp2a_3_Y.lipolytica
EYSHLLIPSLETLSCMEEPVVRDKAVESINRICEGLPS
>pp2a_3_S.pombe
EYAHVLLSPLENLAATEETVVRDKAVDSLNKVCICLSQ
>pp2a_3_A.niger
EYGHVLLSPLENLAAIEEPLVREKAVESLNKIGEQLSE
>pp2a_3_U.maydis
PYAHLLLGPLENLAAVEESVVREKAAESIVKIAEVLSE
>pp2a_3_D.discoideum
EHAVCLLPPLQILAGAEELVVREKAVESLCKIAKEIPT
```

```
>pp2a_3_A.thaliana
EFAHVLLPPLESLCTVEETCVREKAVESLCKIGSQMKE
>pp2a_3_O.sativa
EHAHVLLPPLETLCTVEETCVRDKAVESLCRIGAQMKE
>pp2a_3_C.elegans
DHVHCLLLPLENLATVEETVVRDKAVESLRKIADKHSS
>pp2a_3_D.melanogaster
EFAMYLIPPLESLATVEETVVRDKAVESLRTVAAEHSA
>pp2a_3_X.tropicalis
EFVHCLLPPLESLATVEETVVRDKAVESLRAISHEHSP
>pp2a_3_D.rerio
EFVHCLLPPLESLATVEETVVRDKAVDSLRKISQEHSP
>pp2a_3_A.mellifera
EYVHCLLKPLESLATVEETVVRDKAVESLRTIAAQHSS
>pp2a_4_M.musculus                              49
DLEAHFVPLVKRLAGGDWFTSRTSACGLFSVCYPRVSS
>pp2a_4_S.cerevisiae
QLFSDFVPLIEHLATADWFSSKVSACGLFKSVIVRIKD
>pp2a_4_O.luminarius
GIGKYFVPLIERLAQGDWFTARVSACGLFATAFERSEE
>pp2a_4_Y.lipolytica
HVAQTILPLVKRLSSAEWFSSKVSATGLYAAAIRHCPT
>pp2a_4_S.pombe
QLEQYFVPLVQRLSTAEWFTSRASSAGLYCAAYSQSEN
>pp2a_4_A.niger
QIEENFIPMVLRLSKADWFTSKVSATGLYCVPYRKASP
>pp2a_4_U.maydis
QIEEHYIPLLKRLSGGDWFTSRTSSTSLFSAVYPKAKP
>pp2a_4_D.discoideum
SFEESFLPLLFSLSKADWFTSRTSACGLFTVSYPRANA
>pp2a_4_A.thaliana
DLVESFVPLVKRLAGGEWFAARVSACGIFHVAYQGCTD
>pp2a_4_O.sativa
DIVDWFVPVVKRLAAGEWFTARVSSCGLFHIAYPSAPD
>pp2a_4_C.elegans
SLEEHFVPMLRRLATGDWFTSRTSACGLFSVVYPRVSP
>pp2a_4_D.melanogaster
DLEIHVVPTLQRLVSGDWFTSRTSACGLFSVCYPRVTQ
>pp2a_4_X.tropicalis
DLEAHFVPLVKRLASGDWFTSRTSACGLFSVCYPRVSS
>pp2a_4_D.rerio
DLEVHFVPLVKRLASGDWFTSRTSACGLFSVCYPRVSS
>pp2a_4_A.mellifera
DLEEQFVPLIHRLATGDWFTSRTSACGLFSVCYPRVSP
>pp2a_5_M.musculus                              3.9E-02
AVKAELRQYFQNLCSDDTPMARRAAASKLGEFAKVLEL
>pp2a_5_S.cerevisiae
SLRKNILALYLQLAQDDTPMVKRAVGKNLPILIDLLTQ
>pp2a_5_O.luminarius
ELRNTLTGMFRDLCADETPMVRRAAAQNLGKIAIVSSG
>pp2a_5_Y.lipolytica
EAVPELLKQYGELTRDDTPMVRRAAATHLPAVIEALPA
>pp2a_5_S.pombe
AVKVSLRQSFSHLCHDEAPMVRRPAATNCAKFVFLVTK
>pp2a_5_A.niger
SSQQTLRQYYGGLVHDDTPMVRRQSGNNLAKFIKELNT
>pp2a_5_U.maydis
TTQEELRKMFTALCNDDTPMVRRAAARDLGPFAKNLSK
>pp2a_5_D.discoideum
EMKKSLRKTFGGLCHDDTPMVKRAAATNLGSFAKQIEK
>pp2a_5_A.thaliana
VLKTELRATYSQLCKDDMPMVRRAAASNLGKFATTVES
```

>pp2a_5_O.sativa
QLKAELRTIYGQLCQDDMPMVRRAAASNLGKFAATVEQ
>pp2a_5_C.elegans
AIKSELKSMFRTLCRDDTPMVRRAAAAKLGEFAKVFEK
>pp2a_5_D.melanogaster
PVKAELRANFRKLCQDETPMVRRAAANKLGEFAKVVET
>pp2a_5_X.tropicalis
TVKAELRQHFRNLCSDDTPMVRRAAASKLGEFAKVLEL
>pp2a_5_D.rerio
TVKAEIRQHFRTLCSDDTPMVRRAAASKLGEFAKVLEL
>pp2a_5_A.mellifera
TVKACLRNHFRNLCQDDTPMARRSAASHLGEFAKVMEI
>pp2a_6_M.musculus 6.4E-02
NVKSEIIPMFSNLASDEQDSVRLLAVEACVNIAQLLPQ
>pp2a_6_S.cerevisiae
EDWDYISNIFQKIINDNQDSVKFLAVDCLISILKFFNA
>pp2a_6_O.luminarius
FIVNELLTMFAALTSDDQDSVRLLVVEDCVVLGKLLSA
>pp2a_6_Y.lipolytica
EADDEIYSMFKAQVGDDQDSVRLLSVNVLIAKAEKLKR
>pp2a_6_S.pombe
EAIDEFIPLFNSLSNDDQDSVRLLSFDIMVSLAEVLKS
>pp2a_6_A.niger
IVIDEMIPLFQHLATDEQDSVRLLTVDVLISIAEEIPK
>pp2a_6_U.maydis
LVVSDIIPLYRKLSSDDQDSVRLLTVQDLIAIAESLNH
>pp2a_6_D.discoideum
SVKSEILPLFQSLSTDEQDSVRLLGVENCALLGSMLTN
>pp2a_6_A.thaliana
FLIAEIMTMFDDLTKDDQDSVRLLAVEGCAALGKLLEP
>pp2a_6_O.sativa
YLKTEVMSIFDDLTQDDQDSVRLLAVEGCAALGKLLEP
>pp2a_6_C.elegans
AVIEGLHSSLTDLHVDEQDSVRLLTVESAIAFGTLLDK
>pp2a_6_D.melanogaster
YLKSDLIPNFVQLAQDDQDSVRLLAVEACVSIAQLLPQ
>pp2a_6_X.tropicalis
NVKSEIIPMFSNLASDEQDSVRLLAVEACVNIAQLLPQ
>pp2a_6_D.rerio
YVKSDIIPLFTALASDEQDSVRLLAVEAGVSIATLLPQ
>pp2a_6_A.mellifera
YVKADLIPMFVILAQDEQDSVRLLAIEACVSIAALLPQ
>pp2a_7_M.musculus 2.2E-04
DLEALVMPTLRQAAEDKSWRVRYMVADKFTELQKAVGP
>pp2a_7_S.cerevisiae
SHTQDLLNSAVKLIGDEAWRVRYMAADRFSDLASQFSS
>pp2a_7_O.luminarius
DCANKIVPIVLKLAADKSWRVRYAVAQQIYEMCDVVGA
>pp2a_7_Y.lipolytica
QHTSSLIEFALALLHDPSWRVRYMCADRFEKLAESLTS
>pp2a_7_S.pombe
EIRHYLLQPLRSFVSDSSWRTRYMVAANFVKLAKVVGP
>pp2a_7_A.niger
PSHGVLLTSLRNLFEDKSWRVRYMVADRYEKIAKAVHE
>pp2a_7_U.maydis
ESKNYLLPSIRSAVQDKSWRVRYMVADHFVKLASAVGE
>pp2a_7_D.discoideum
ENIQFILPTIKASSLDKSWRVRYMVARLLKELCESMGT
>pp2a_7_A.thaliana
DCVARILPVIVNFSQDKSWRVRYMVANQLYELCEAVGP
>pp2a_7_O.sativa
DCVAHILPVIVNFSQDKSWRVRYMVANQLYELCEAVGP

>pp2a_7_C.elegans
NKKKLIEPILIELFDDKSWRVRYMVAEKLIEIQNVLGE
>pp2a_7_D.melanogaster
DVEHLVLPTLRQCASDSSWRVRYMVAEKFVDLQKAVGP
>pp2a_7_X.tropicalis
ELEPLVMPTLRQAAEDKSWRVRYMVADKFTELQKAVGP
>pp2a_7_D.rerio
DLEALVMPTLRQAAEDKSWRVRYMVADKFSELQKAVGP
>pp2a_7_A.mellifera
DVEQLVMPTLRQCASDQSWRVRYMVADKFTDLQKAVGP
>pp2a_8_M.musculus 7.6E-03
ITKTDLVPAFQNLMKDCEAEVRAAASHKVKEFCENLSA
>pp2a_8_S.cerevisiae
AYIDELVQPFLNLCEDNEGDVREAVAKQVSGFAKFLND
>pp2a_8_O.luminarius
VAAKGLFDAYVALLGDSEGEVRISAAGKISEFCSLAGA
>pp2a_8_Y.lipolytica
EMERAFVPEFIKFMQDGEAEVRTAVAKQVPGFCRLVTP
>pp2a_8_S.pombe
LIKDELIKPFVLLMKDTEQEVRRAIATQIPGFCELLDK
>pp2a_8_A.niger
VVTRDMVPSFVKLLKDTEAEVRTAIAGQIPGFCNLIDR
>pp2a_8_U.maydis
VVRDELVMAFVHLLKDNEAEVRTAGAGQIPGFAKLVDQ
>pp2a_8_D.discoideum
ITKTELIGAFVKLLKDTEAEVRTEASLRIADVCSLLTK
>pp2a_8_A.thaliana
CTRTDLVPAYVRLLRDNEAEVRIAAAGKVTKFCRLLNP
>pp2a_8_O.sativa
HSREQLVPAYVRLLRDNEAEVRIAAAGKVTKFCRILSP
>pp2a_8_C.elegans
MDTTHLVNMYTNLLKDPEGEVRCAATQRLQEFALNLPE
>pp2a_8_D.melanogaster
ITRVDLVPAFQYLLKDAEAEVRAAVATKVKDFCANLDK
>pp2a_8_X.tropicalis
ITKTDLVPAFQNLMKDCEAEVRAAASHKVKEFCENLSA
>pp2a_8_D.rerio
ITKNDLVPAFQNLLKDCEAEVRAAAANKVKVFCENLPE
>pp2a_8_A.mellifera
ITKTDLVPAFQVLLKDIEAEVRAAAADKVRDFCQNLDK
>pp2a_9_M.musculus 0.014
VIMTQILPCIKELVSDANQHVKSALASVIMGLSPILGK
>pp2a_9_S.cerevisiae
IILNKILPAVQNLSMDESETVRSALASKITNIVLLLNK
>pp2a_9_O.luminarius
YSAEKILPKVHELANDSSQHVRAALAEAVLGLAPTMGK
>pp2a_9_Y.lipolytica
ANLDKIVANVEELSQDSSQHVRAALGSEISALAPLLGK
>pp2a_9_S.pombe
IVLEEIIPVIQELINDPAQHVRAALGMNIGALAPQLGK
>pp2a_9_A.niger
TLLNEIMTSVEDLVSDPSQHVRAALGTQISGLAPILGK
>pp2a_9_U.maydis
IILARLMPCVRDLAGDNSQHVRAALGMQISGMAPLLGK
>pp2a_9_D.discoideum
MNIKTILPCVKDLVSDSSQHVRAALAQVIMSLAPIYGK
>pp2a_9_A.thaliana
LAIQHILPCVKELSSDSSQHVRSALASVIMGMAPILGK
>pp2a_9_O.sativa
LAIQHILPCVKELSSDSSQHVRSALASVIMGMAPVLGK
>pp2a_9_C.elegans
IICNSLLNVAKELVTDGNQLVKSELAGVIMGLAPLIGK

>pp2a_9_D.melanogaster
IILSSILPYVRDLVSDPNPHVKSALASVIMGLSPMLGA
>pp2a_9_X.tropicalis
VIMTQILPCVKELVSDANQHVKSALASVIMGLSPILGK
>pp2a_9_D.rerio
IIMTHILPCVKELVSDTNQHVKSALASVIMGLSTILGK
>pp2a_9_A.mellifera
IIMTQILPIVKELVSDPNQHVKSALASVIMGLSPILGK
>pp2a_10_M.musculus 0.081
NTIEHLLPLFLAQLKDECPEVRLNIISNLDCVNEVIGI
>pp2a_10_S.cerevisiae
QVINNFLPILLNMLRDEFPDVRLNIIASLKVVNDVIGI
>pp2a_10_O.luminarius
TTVEKLLPVFFILLKDEFPDVRLNIISKLEQVNTVIGV
>pp2a_10_Y.lipolytica
KTIETLLPTFLQMLKDDFPDVRLNIISKLHLVNKVIGI
>pp2a_10_S.pombe
KTTEYLLPMFLELLKDENPEVRLNIISKLEVVNKVVGI
>pp2a_10_A.niger
ETIAHLLPMFLQMLKDEFPDVRLHIISKLELVNKVIGI
>pp2a_10_U.maydis
ATIEHLLPLFLQLLKDEFPDVRLNIISKLEQVNEVIGI
>pp2a_10_D.discoideum
DTLTHLLELFLHLLKDDFPDVRLNIISKLDQVSKVIGI
>pp2a_10_A.thaliana
STIEHLLPIFLSLLKDEFPDVRLNIISKLDQVNQVIGI
>pp2a_10_O.sativa
ATIEQLLPIFLSLLKDEFPDVRLNIISKLDQVNQVIGI
>pp2a_10_C.elegans
QTVSELLPIYMQLLNDQTPEVRLNIISSLDKVNEVIGA
>pp2a_10_D.melanogaster
QTVEQLLPLFLIQLKDECPEVRLNIISNLDCVNDVIGI
>pp2a_10_X.tropicalis
NTIEHLLPLFLAQLKDECPEVRLNIISNLDCVNEVIGI
>pp2a_10_D.rerio
NTVEHLLPLFLAQLKDECPEVRLNIISNLDCVNEVIGI
>pp2a_10_A.mellifera
NTIEHLLPLFLSQLRDECSEVRLNIISNLECVNEVIGI
>pp2a_11_M.musculus 0.011
QLSQSLLPAIVELAEDAKWRVRLAIIEYMPLLAGQLGV
>pp2a_11_S.cerevisiae
LLSDSLLPAITELAKDVNWRVRMAIIEYIPILAEQLGM
>pp2a_11_O.luminarius
MLSTELLPAIKELAEDKHWRVRLAIIEYIPVLAQQIGT
>pp2a_11_Y.lipolytica
LLSQSLLPAVSDLAQDKQWRVRLAIIEYIPLLATQLGV
>pp2a_11_S.pombe
LLSQSLLPAIVTLAEDKQWRVRLAIIDYIPLLAQQLGV
>pp2a_11_A.niger
LLSQSLLPAIVQLAEDKQWRVRLAIIEYIPLLASQLGV
>pp2a_11_U.maydis
LLSQSLLPAIVELAEDKQWRVRQAIIEYIPLLANQLGV
>pp2a_11_D.discoideum
MLSQSLLPAIVELAEDHQWRVRLAIIDYIPLLASQLGV
>pp2a_11_A.thaliana
LLSQSLLPAIVELAEDRHWRVRLAIIEYVPLLASQLGI
>pp2a_11_O.sativa
LLSQSLLPAIVELAEDRHWRVRLAIIEYIPLLASQLGV
>pp2a_11_C.elegans
QLSTSLLPAIVGLAEDGKWRVRLAIVQFMPLLASQLGQ
>pp2a_11_D.melanogaster
QLSQSLLPAIVELAEDSKWRVRLAIIEYMPALAGQLGQ

```
>pp2a_11_X.tropicalis
QLSQSLLPAIVELAEDAKWRVRLAIIEYMPLLAGQLGV
>pp2a_11_D.rerio
QLSQSLLPAIVELAEDAKWRVRLAIIEYMPLLAGQLGV
>pp2a_11_A.mellifera
QLSQSLLPAIVELAEDSKWRVRYAIIEYMPLLAGQLGV
>pp2a_12_M.musculus                              0.095
FFDEKLNSLCMAWLVDHVYAIREAATSNLKKLVEKFGK
>pp2a_12_S.cerevisiae
FFDQQLSDLCLSWLWDTVYSIREAAVNNLKRLTEIFGS
>pp2a_12_O.luminarius
SGDDLLNSLCLQWLQDSVYSIREAAANNLFRLTEIFGA
>pp2a_12_Y.lipolytica
FFDKELGPLCMTWLWDSVYSIREAATQNLKKLTKVFGV
>pp2a_12_S.pombe
FFNEKMGNLCMSWLEDHVYSIREAAIKNLRKLTEIFGL
>pp2a_12_A.niger
FFDEQLSELCMGWLGDTVFSIREAATQNLRKLTEVFGV
>pp2a_12_U.maydis
FFDEQLSNLCMSWLGDTVFSIREAATVNLKKLTDVFGV
>pp2a_12_D.discoideum
FFDEKLGNLCMTWLGDPVFSIREAATNNLKKLTEVFGV
>pp2a_12_A.thaliana
FFDDKLGALCMQWLQDKVYSIREAAANNLKRLAEEFGP
>pp2a_12_O.sativa
FFDDKLGALCMQWLEDKVFSIRDAAANNLKRLAEEFGP
>pp2a_12_C.elegans
FFDEKLLPLCLNWLTDHVFSIREASTLIMKELTQKFGG
>pp2a_12_D.melanogaster
FFDQKLRGLCMGWLNDHVYAIREAATLNMKKLVEQFGA
>pp2a_12_X.tropicalis
FFDEKLNSLCMAWLVDHVYAIREAATSNLKKLVEKFGK
>pp2a_12_D.rerio
FFDEKLNSLCMAWLIDHVYAIREAATCNLMKLVEKFGA
>pp2a_12_A.mellifera
FFDEKLNSLCMTWLVDHVYAIREAATLNLKKLVEKFGP
>pp2a_13_M.musculus                              0.051
WAHATIIPKVLAMSGDPNYLHRMTTLFCINVLSEVCGQ
>pp2a_13_S.cerevisiae
WCRDEIISRLLKFDLQLLEVSRFTILSALTTLVPVVSL
>pp2a_13_O.luminarius
WALEYIFPRIKELMSSHHYLYRLTVLRAVSLLAPAVGE
>pp2a_13_Y.lipolytica
WAKDEILPHIIVVAADSNYLYRLTALCAVTTLIPVVDE
>pp2a_13_S.pombe
WATETIIPKFLAMRSHPNYLYRMTTIFAISEIAPALNA
>pp2a_13_A.niger
WAKGSIIPKVMGMGQHTNYLYRMTTCFAISTLAPVVSL
>pp2a_13_U.maydis
WARQTIIPKVLQMGTHPNYLYRMTTIFAITTMAPSLDT
>pp2a_13_D.discoideum
WAKNNIIPKVLSLHSHPNYLYRMTTLFSISTLSTVVGG
>pp2a_13_A.thaliana
WAMQHLVPQVLDMVNNPHYLHRMMVLRAISLMAPVMGS
>pp2a_13_O.sativa
WAMQHIIPQVLEKINNPHYLYRMTILQAISLLAPVMGA
>pp2a_13_C.elegans
WASTNIVPKMQKLQKDTNYLQRMTCLFCLNTLSEAMTQ
>pp2a_13_D.melanogaster
WAEQAIIPMILVMSRNKNYLHRMTCLFCLNVLAEVCGT
>pp2a_13_X.tropicalis
WAQATIIPKVLAMSNDPNYLHRMTTLFCINVLSEVCGQ
```

```
>pp2a_13_D.rerio
WAQNTIVPKVLGMANDSNYLHRMTTLFCINALSEACGQ
>pp2a_13_A.mellifera
WAQNTVIPKVLAMSRDQNYLHRMTCLFCINVLAEVCGP
>pp2a_14_M.musculus                              4.5E-03
ITTKHMLPTVLRMAGDPVANARFNVARSLQKIGPILDN
>pp2a_14_S.cerevisiae
VVTEQLLPFISHLADDGVPNIRFNVAKSYAVIVKVLIK
>pp2a_14_O.luminarius
VILGEILPIIKHATTDTVPNVRFNAAKALSPLIKAIDV
>pp2a_14_Y.lipolytica
MIKTSILPFIAELINDPIPNIRFNVAKTYTELVRALHE
>pp2a_14_S.pombe
VIEKQILPTLEQLVNDPIPNIRFNVAKAFEVLKPVLAA
>pp2a_14_A.niger
IIENSILPILDRLVSDEIPNIRFNVAKSYAVLIDTLRR
>pp2a_14_U.maydis
AITGDVLETVLPMVSDPIPNIRFNVAKAFEVLSSVLVK
>pp2a_14_D.discoideum
VISSSMVPLLAKMVSDKVPNIRFNVAKTFQTIIPLLDS
>pp2a_14_A.thaliana
ITCSKFLPVVVEASKDRVPNIKFNVAKLLQSLIPIVDQ
>pp2a_14_O.sativa
ITCQQLLPVVINSSKDRVPNIKFNVAKVLQALIPILDQ
>pp2a_14_C.elegans
QILKEIMPIVKDLVEDDVPNVRFNAAKSLKRIGKNLTP
>pp2a_14_D.melanogaster
ITTKLLLPTVLLLAADPVANVRFNVAKTLQKISPFLEA
>pp2a_14_X.tropicalis
ITTKHMLPTVVRMAGDAVANVRFNVAKSLQKIGPTLDN
>pp2a_14_D.rerio
ITTKHMLPVVLKMSTDQVANVRFNVAKSLQKIGPVLES
>pp2a_14_A.mellifera
ITTRVMLPTVLGMATDNVANVRFNVAKTLQKIGPYLEP
>ada2_M.musculus                                 no match
PAHFGQLECLKLIASQKF-DKRIGYLGAMLLLDERQDV
>ada2_X.laevis
PAHFGQLECLKLIASQKF-DKRIGYLGAMLLLDERQDV
>ada2_D.rerio
PAHFGQLECLKLIASQKF-DKRIGYLGAMLLLDERQDV
>ada2_D.melanogaster
PAHFGQLECLKLTASTRF-DKRIGYLGAMLLLDERQDV
>ada2_C.elegans
PAHFGQMECMKLVAHPRF-DKRIGYLGAMLLLDERSEV
>ada2_A.aegypti
PAHFGQMETLKLAASSKF-DKRIGYLGAMLLLDERQDV
>ada2_D.discoideum
PTQFGQMECLKLIVSPSY-DKRIGYLGLMLLLDEKQEV
>ada2_U.maydis
PAHFGQIECLKLVATPRF-DKRLGYLGIMLLLDENTEV
>ada2_A.thaliana
PTHFGQMECLKLIASPGF-EKRIGYLGLMLLLDERQEV
>ada2_N.crassa
RTHFGQIECLKLLASPRF-DKRLGHLATSLLLDENQEV
>ada2_O.sativa
PTHFGQMECLKLIAAAGF-EKRIGYLGLMLLLDERQEV
>ada2_Y.lypolytica
RTHFGQVECLKLIASPRF-EKRLGYLGTMLLLDENQET
>ada2_O.luminarius
ATHFGQMECVRLTARNGF-EKRIGYLGLMLLLDEDQEV
>ada2_S.pombe
PTHFGQIECLKLLSSSRF-DKRLGYLAAMLLLDENQEV
```

>ada2_S.cerevisisae
KTHFGQVESINLIASDDF-DKRLGYLAATLLLDESEDL
>ada3_M.musculus 90
DVHLLMTNCIKNDLNHSTQFVQGLALCTLGCMGSSEMC
>ada3_X.laevis
DVHLLMTNCIKNDLNHSTQYVQGLALCTLGCMGSAEMC
>ada3_D.rerio
DVHLLMTNCIKNDLNHSTQYVQGLALCTLGCMGSSEMC
>ada3_D.melanogaster
DVHLLITNCLKNDLNSSTQFVVGLALCTLGAIASPEMA
>ada3_C.elegans
EVHMLVTNSLKNDLTCSTQFVSGLALCTLGSICSAEMC
>ada3_A.aegypti
DVHVLLTNCLKNDLNNSTQFIVGTALCTLAAIASPEMA
>ada3_D.discoideum
EVLLLATNCIRGDIMNSNQFIVGVSLCAFGNICSTAMA
>ada3_U.maydis
EVLTLVTNGLKNDMEHSNMYVCGLALCTFANIASEEMS
>ada3_A.thaliana
EVLMLVTNSLKQDLNHTNQYIVGLALCALGNICSAEMA
>ada3_N.crassa
EVLTLVTNSLQNDLNHSNQYVVGLALCTLGNIASVEMS
>ada3_O.sativa
EVLMLVTNSLKQDLNHSNQFIVGLALCALGNICSAEMA
>ada3_Y.lypolytica
ETLTLVTNSLSNDLNHPNQYVVALALTTLANIASTEMG
>ada3_O.luminarius
EVTMLVTNSVKNDLSHKNHYVVGLGLCMLGSICSAEMA
>ada3_S.pombe
EVLTLLTNSLQNDLKSRDKFIVGLALSAFGNVAGPELA
>ada3_S.cerevisisae
DLLTLLTNMLNNDLHHPNKYAVSLALTSLGFLSSPELA
>ada4_M.musculus 1.4
EMCRDLAGEVEKLLKTSNSYLRKKAALCAVHVIRKVPE
>ada4_X.laevis
EMCRDLAGEVEKLLKTSNSYLRKKAALCAVHVIRKVPE
>ada4_D.rerio
EMCRDLAGEVEKLLKTSNSYLRKKAALCAVHVIRKVPE
>ada4_D.melanogaster
EMARDLASEVERLMKSPNTYIRKKATLCAFRVIRRVPE
>ada4_C.elegans
EMCRDLANEVEKIIKQNNAYLKKKAALCAFRIVRKVPE
>ada4_A.aegypti
EMARDLSHEIERLIASSNAFLRKKAILCAFRMVRRVPE
>ada4_D.discoideum
AMARDISPEIEKVISHSNPYIRKKAALCAIRVLRKVPD
>ada4_U.maydis
EMSRDLCNEIEKLMGSSNTYIRRKAAICAMRIVRKVPD
>ada4_A.thaliana
EMARDLAPEVERLLQFRDPNIRKKAALCAIRIIRKVPD
>ada4_N.crassa
EMSRDLFPQIETLISTTNPYIRRKAALCAMRICKKVPD
>ada4_O.sativa
EMARDLSPEVERLLQSREPNTKKKAALCSIRIVRKVPD
>ada4_Y.lypolytica
EMGRDLFQTVDKIMSSSNPYLKKKAAVCAARISSRVPE
>ada4_O.luminarius
EMARDVAGEVEQLMGHGNSYVRKKAALTATRVIKKVPE
>ada4_S.pombe
ELARDLSNDIAELCSNHHNYISKKAVLCALRVIQKEPD
>ada4_S.cerevisisae
ELARDLYPDVENIIKNSRDFLLKKALQCAAKLIFKDVS

>ada5_M.musculus 22
ELMEMFLPATKNLLNEKN-GVLHTSVVLLTEMCERSPD
>ada5_X.laevis
ELMEMFLPATKNLLNEKN-GVLHTSVVLLTEMCERSPD
>ada5_D.rerio
ELMEMFLPATKNLLSEKN-GVLHTSVVLLTEMCERSPD
>ada5_D.melanogaster
ELMEIFLPATRSLLSEKN-GILITGVTLITEMCENSSD
>ada5_C.elegans
ELMEVFIPCTRSLLGEKN-GVLMGATTLVTEMCEKSPD
>ada5_A.aegypti
ELMEEYIPKCSHFLNDKN-GILISTITLVTEMCEQSPV
>ada5_D.discoideum
DLTENYIPKIKALLSERN-AVILTALTLIIEICEMDST
>ada5_U.maydis
DLIDHFVDRTQQLLSDKN-GVLLCAVTLAIEICRQDDE
>ada5_A.thaliana
DLSENFINPGAALLKEKH-GVLITGVHLCTEICKVSSE
>ada5_N.crassa
DLQEHFVEKAAQLLSDRN-GVLLCGLTLVTSLCEADEA
>ada5_O.sativa
DLAENFMGSAVSLLKEKH-GVLISAVQLCAELCKASKE
>ada5_Y.lypolytica
ELAEIFVEKAKILLTDKN-GVLLCGLTLATDICVQDDE
>ada5_O.luminarius
ELTEGFVPAAEKLLSDRH-GVLLAACTLATEMCEDDDD
>ada5_S.pombe
DLESLYIEKTDELLHSKS-GVLMAALAFAISACKINPS
>ada5_S.cerevisisae
LLEIFNIEDITKILSSHSIGVLLGVTKIIQSILLIGLN
>ada6_M.musculus no match
KLVPQLVRILKNLIMSGYSFLQVRILRLLRILGRNDDD
>ada6_X.laevis
KLVPQLVRILKNLIMSGYSFLQVRILRLLRILGRNDDD
>ada6_D.rerio
KLVPQLVRILKNLIMSGYSFLQVRILRLLRILGKGDDD
>ada6_D.melanogaster
KIVPNLVRILKNLILGGYSFLQVKILRLLRILGHNDPD
>ada6_C.elegans
KLVPNLVRILKNLLMSGYSFLQVKILRLLRVLGKDDVR
>ada6_A.aegypti
SSIPTLVRTLKTLIVSGYSFLQVKILRLLRILGHGDTA
>ada6_D.discoideum
KMVPQLVRILKSLTSSGYLFLQVKILRLLRILGQNDPE
>ada6_U.maydis
RAVPLLVQHLKSLVTTGYSFLQVKILRLLRILGKENAQ
>ada6_A.thaliana
KCTEGLVKTLRDIANSPYSFLHIRLLKLLRVLGQGDAD
>ada6_N.crassa
QFVPVLVRTLKGLASSGYAFLQVKILRLLRVLARGDAQ
>ada6_O.sativa
NCLDGLVRILRDVSNSSYAFLHIRVLKLMRILGQGDAD
>ada6_Y.lypolytica
PVVPTLVKLLRQLCTSAYAFLQVKILGLLRVLGAGDAS
>ada6_O.luminarius
AQVPQLCKVLKSLIYAGKSFLQVAILRLLRVLGRGDAD
>ada6_S.pombe
SQADDLIYRIRQLSTSTYSFLQVKILQFLSILGQNNPK
>ada6_S.cerevisisae
LLLRDFFIRLENMNSKNIEFLQCEIIYTLKLYFQVGEL
>ada7_M.musculus no match
EAMNDILAQVATNTETSKNVGNAILYETVLTIMDIKSE

>ada7_X.laevis
EAMNDILAQVATNTETSKNVGNAILYETVLTIMDIKSE
>ada7_D.rerio
EAMNDILAQVATNTETSKNVGNAILYETVLTIMDIKSE
>ada7_D.melanogaster
EAMNDILAQVATNTETSKNVGNTILYETVLSIMDIRSE
>ada7_C.elegans
EEMNDILAQVATNTETAKNVGNAILYETVLTIMEIKSE
>ada7_A.aegypti
EIMNDVLAQVATNTETNKNAGNAILYETVLTIMNVESE
>ada7_D.discoideum
DAMNDILAQVSTNTDSTKNVGNAILYECVQTIMTIESE
>ada7_U.maydis
ETMNDILAQVATNTEASKNVGNSILYETVLTILEIDAD
>ada7_A.thaliana
DCMNDILAQVASKTESNKNAGNAILYECVQTIMSIEEN
>ada7_N.crassa
EQINDILAQVATNTDATKNVGNSILYEAVLTILDIEAD
>ada7_O.sativa
EFVNDILAQVATKTESNKNAGNAILYECVETIMGIEAT
>ada7_Y.lypolytica
DAMNDVLAQVASNTDSAKNVGSSVLYECVRTIFAVEAD
>ada7_O.luminarius
DAMSDILAQIASNTDGAKNAGNAILYEAVETIIAIEAV
>ada7_S.pombe
DKMSDLLAQVCTNTDSSRNAGNAILYQAVRTILDLNSD
>ada7_S.cerevisisae
DNFCDLLTRIATNTDSTKNSGQAILYETVKTIFSLDLN
>ada8_M.musculus 43
GLRVLAINILGRFLLNNDKNIRYVALTSLLKTVQTDHN
>ada8_X.laevis
GLRVLAINILGRFLLNNDKNIRYVALTSLLKTVQTDHN
>ada8_D.rerio
GLRVLAVNILGRFLLNNDKNIRYVALTSLLKTVQADHN
>ada8_D.melanogaster
GLRVLAVNILGRFLLNSDKNIRYVALNTLLRTVHADTS
>ada8_C.elegans
GLRILAVNILGRFLLNTDKNIRYVALNTLLKTVHVDYQ
>ada8_A.aegypti
SLRVLAVNILGRFLLNSDKNIRFVGLLTLVRTVQRDMT
>ada8_D.discoideum
GLKVMAINILGRFLLNRDNNIRYVALNTLSRVVNTDIQ
>ada8_U.maydis
GLRVMAINILGKFLSNRDNNIRYVALNTLSKVVSMDTN
>ada8_A.thaliana
GLRVLAINILGKFLSNRDNNIRYVALNMLMRSLTVDSQ
>ada8_N.crassa
GLRVLGVNILGKFLANKDNNIRYVALNTLVKVVAIDTN
>ada8_O.sativa
GLRVLAINILGRFLSNRDNNIRYVALNMLMKAMEVDTQ
>ada8_Y.lypolytica
GLRVLGVNILGKFLATTDNNTRYVALNTLLTVIDIEPA
>ada8_O.luminarius
GLRVLAVNILGRFLQNKDNNIRYVALNTLAKVVEVDMQ
>ada8_S.pombe
SLRVLGVNILAKFLGNRDNNTRYVALNMLKLVVNSEEN
>ada8_S.cerevisisae
PLRVLGINILAKFLAGKDNNTKYVSLNTLLKVVPQEPT
>ada9_M.musculus 3.8
NAVQRHRSTIVDCLKDLDVSIKRRAMELSFALVNGNNI
>ada9_X.laevis
NAVQRHRSTIVDCLKDLDVSIKRRAMELSFALVNGNNI

>ada9_D.rerio
NAVQRHRSTIVDCLKDLDVSIKRRAMELSFALVNGNNI
>ada9_D.melanogaster
SAVQRHRTTILECLKDPDVSIRRRAMELSFALINAQNI
>ada9_C.elegans
QAVQRHRNVVVECLKDPDISIRKRAMELCFALMNRTNI
>ada9_A.aegypti
TAVQRHRITILECLTDSDSSIQKCAMELSFTLVNSQNI
>ada9_D.discoideum
QAVQRHRNTIVECLKDPDVSIRCRALDLIYSLVTESNI
>ada9_U.maydis
NAVQRHRNIILDCLRDGDISIRRRALELSYALINESNV
>ada9_A.thaliana
QAVQRHRATILECVKDSDASIQKRALELIYLLVNENNV
>ada9_N.crassa
NAVQRHRNTILECLRDPDISIRRRALDLSFTLINESNV
>ada9_O.sativa
QAVQRHRATILECVKDADVSIRKRALELVYLLVNDANA
>ada9_Y.lypolytica
AAVQRHRNTIVECLRDADVSIRRRALAVAYALINESNV
>ada9_O.luminarius
QAIQRHRAIIVNCVKDADITIRRSALQLVYGLVNAKNV
>ada9_S.pombe
NAVQRHRSTILACLNDVDSSIQSRALELSTFLVNEANV
>ada9_S.cerevisisae
TAVQRHRKFISHCLQDTDVSIRMRALELSFAILDDSNL
>ada10_M.musculus 110
NNIRGMMKELLYFLDSCEPEFKADCASGIFLAAEKYAP
>ada10_X.laevis
NNIRGMMKELLYFLDSCEPEFKADCASGIFLAAEKYAP
>ada10_D.rerio
NNIRGMMKELLYFLDSCDPEFKADCASGIFLAAEKYAP
>ada10_D.melanogaster
QNIRTMTKELLLFLEKADAEFKAQCSSGMILAAERYSP
>ada10_C.elegans
TNIAIMTKEVLIFLETADAEFKSECASRMYIATERYSP
>ada10_A.aegypti
QNIETIVRELLKYLETAEAEMKGTCSSRIVLAAEMYSP
>ada10_D.discoideum
SNIRVLVRELLNFLLIADAQFKSELVAKLCIVTEKYAP
>ada10_U.maydis
SNVRVLTRELLSFLEVADNEFKLGMTTQICLAAEKFAP
>ada10_A.thaliana
NNVKPLAKELIEYLEVSEQDFKGDLTAKICSIVEKFAP
>ada10_N.crassa
SNVRVLIRELLAFLEVADNEFKPNMTSQIGIAADRYAP
>ada10_O.sativa
ANAKSLTKELVDYLEVSDQDFKDDLTAKICSIVEKFSQ
>ada10_Y.lypolytica
SNVRVIVRELLTFLESADAEFKPSVTAQIAIAAEKYAP
>ada10_O.luminarius
KNVTTLSHELLEYLEVCDEEFKCELAKKISSLALKFSP
>ada10_S.pombe
ANVRFMVRELLSFLDNVSDELRGSTAQYITEVTNAFAP
>ada10_S.cerevisisae
SNLVELVNELMKFLAKQDEDSKDLIIYTIDHLIDTFDT
>ada_11_M.musculus no match
PSKRWHIDTIMRVLTTAGSYVRDDAVPNLIQLITNSVE
>ada_11_X.laevis
PSKRWHIDTIMRVLTTAGGYVRDDAVPNLIQLITNSTE
>ada_11_D.rerio
PSKRWHIDTIMRVLTTAGSYVRDDSVPNLIQLITNSVE

>ada_11_D.melanogaster
PTTRWHLDTQLSVLIAAGNYVRDDVVSSTIQLVSSSPV
>ada_11_C.elegans
PNHEWHLDTMITVLRLAGKYVPDEVVSCMIQMISANEQ
>ada_11_A.aegypti
PSIHWHLDVLLKVLTISGNNIRDDVIASTIQLISNSPP
>ada_11_D.discoideum
PNKRWQIDTILRVMSIAGNFIPDEVPSNLIQLISSTPE
>ada_11_U.maydis
PNKRWHIDTVLRVLKLAGNYVREEILSAFIRLVCHTPE
>ada_11_A.thaliana
PEKIWYIDQMLKVLSEAGTYVKEDVWHALIVVITNAPD
>ada_11_N.crassa
PNKRWHVDTMLRVLTLAGNYVKEPILSSFIRLIATTPE
>ada_11_O.sativa
QDKLWYLDQMFKVLSLAGNYVKDDVWHALIVLISNASE
>ada_11_Y.lypolytica
PNKRWHIDTLVRALALAGSHVPENVVSSFIALVVTCDE
>ada_11_O.luminarius
PSKQWYIDTFIALLIRAGQYIDELECNDFMGLVARTPQ
>ada_11_S.pombe
PNKRWHFDTLLRVFKSAGNFVSESTLSTFLRLIASAP-
>ada_11_S.cerevisisae
KDESWKLDVFFNILKLVGSFINYEKINDILIIINNTSQ
>ada_12_M.musculus 280
EMHAYTVQRLYKAI-LGDYPLVQVAAWCIGEYGDLLVS
>ada_12_X.laevis
EMHEYTVQKLYKAILDDISPLVQVSSWCIGEYGDLLVS
>ada_12_D.rerio
EMHAYTVQRLYKALLDDISPLVQVASWCIGEYGDLLVS
>ada_12_D.melanogaster
PEQTYITNRFWESLQVANHPLLQVAVWAIGEYGDLFMY
>ada_12_C.elegans
QLQSYAVSQLYHAAQKDAIPLLQVAFWTIGEFGDLLLQ
>ada_12_A.aegypti
KEQSYISGKMWKSITNMNQPLVQVAVWTLGEYGE----
>ada_12_D.discoideum
ELSSYAVQKLYLALKQDITPLTQVGLWCIGEYGDLLVA
>ada_12_U.maydis
ELQAYTVQKLFSGLHQDFSSLTLAAVWVIGEFGDVLIQ
>ada_12_A.thaliana
DLHGYTVRALYRALHTSFETLVRVAIWCIGEYADLLVN
>ada_12_N.crassa
ELQTYAVQKLYTNLKKDITSLTQAGAWCIGEYGDALLR
>ada_12_O.sativa
ELQGYSVRSLYKALLACGESLVRVAVWCIGEYGEMLVN
>ada_12_Y.lypolytica
ELQLYTVQKLYSALRADFTGLSLASLWLLGEFGHILIR
>ada_12_O.luminarius
QLHGYAARSLYRAACDDYARLCAVAVWVCGEYSDAMVH
>ada_12_S.pombe
ELHEYAVVKLYAALKEDVSALTLSAFWVIGEYGQMLLS
>ada_12_S.cerevisisae
LRKMLTISLNGTSAEISEEGWQLVLIWCIGEYGDLVLN
>ada_13_M.musculus 73
DEVLDILESVLISNMSTSV-TRGYALTAIMKLSTRFTC
>ada_13_X.laevis
DEVLDILESILISNMSASV-TRGFALTAIMKNSTRFNS
>ada_13_D.rerio
DEVLDVLEGLLVSNLSAPV-TRGYSLTAIMKLSTRFSS
>ada_13_D.melanogaster
SDLIAVYYKFLTSAQVSTT-SKQYALVSLAKLSTRLQQ

>ada_13_C.elegans
NDVVGVFESVLPSALTSLW-TKCYGVTALAKLGTRFQS
>ada_13_A.aegypti
NELIDHYRQLLWAPQLSIT-TKQYILVSLAKISVRMEH
>ada_13_D.discoideum
QAVIDIIDLIFRHATTTQA-TRQYSLTSLAKLSSRFSQ
>ada_13_U.maydis
KDVVDLLSSVLDSPYVNGL-IRQFVLTSLAKLHTRLSD
>ada_13_A.thaliana
SDAVDVVENAIKHHLSDVT-TKAMALIALLKISSRFPS
>ada_13_N.crassa
HELIDLFTTILNSNYATQV-TTEYIITALIKLTTRLQD
>ada_13_O.sativa
SDAVDAVEVSLKRYSADVT-TRAMCLVSLLKLSSRFPP
>ada_13_Y.lypolytica
ESVVTMIENLLKSAYASDV-VQEYGVNALVKLSTRINN
>ada_13_O.luminarius
NDVTKLMIAILSEEKYLM--LRPLVMTALAKIAVREPS
>ada_13_S.pombe
SDIVDIIEEVFNSVEASRYIIVQYGLFALTKLSARLGS
>ada_13_S.cerevisisae
SSITDYLLTLQELYTATNLKIINYILTAALKLSVRFHD
>eef_2_S.cerevisiae 0.84
DVPEHFFGELAKGIKDKK--TAANAMQAVAHIANQSNL
>eef_2_C.albicans
DVPVEFFEDLKKQIQSKDAKVSLAALDAYKHIASTNGL
>eef_2_Y.lypolytica
DVPNQLLAATKAKLNQKKDAAVLNALQLLNSLATSSSV
>eef_2_N.crassa
DTPVKTVEALKKQLANKKDTAREKALSAIQAIAQHSEV
>eef_2_S.pombe
DAPSQVFSAISKQLNDKNATARERVLKGLEAVANHGSV
>eef_2_U.maydis
LANLGIEAVILKGLGDKKNTAREGACTLLANLCEQGV-
>eef_2_O.tauris
KISMADVAAMREAQGMKKDDARKAAGEKIAELANAASY
>eef_2_C.neoformans
FTQIGFNDAIVKALNDKKSSAREGACEVISTLCENGA-
>eef_2a_S.pombe
LKTTGFLDGLERAARNKKSFHREAAMIGFATVIKNLGT
>eef_2_P.guilliermondii
VNEWKLSEVLKSFAKPKNSAVREAALILVQQMAIRFGG
>eef_2_K.lactis
IEDWKLAETITKFMKPKNPLVREAGMLLISSLASSFTG
>eef_2_L.elongisporus
INEWKLNEILKSLLKPKNTLVKEGALLIIQQLAQQIAG
>eef_2_C.cinerea
-ADANVISTLHSFATNKKSYERESAAMAFHSLATVIGA
>eef_2_D.hansenii
INEWKLNDIIKSLSKPKNSLVRESAMIIIQQLVMKFGG
>eef_2_A.niger
SQYDFILPEIKKAALDKKNARRESAMLILGALVERFPP
>eef_3_S.cerevisiae 8.7
-VEPYIVQLVPAICTNAGNEIQSVASETLISIVNAVNP
>eef_3_C.albicans
-VEPYVVDLVSEVAVKAGDDVQTAASDALLAIASAITP
>eef_3_Y.lypolytica
-VEPYLVEAITDVLAKVGDQIQDAAAKTAKDIAQCITP
>eef_3_N.crassa
-VEPYLVALLPSVFTAAGDAVKNAAIAAALAIAEAINP
>eef_3_S.pombe
-VEPYLVELLPAVIAKVADAVRDAAIAASKAIVRCTTP

>eef_3_U.maydis
-VEPFIFEVLNSLVEAMGDAVQKASLETLKAFVRVMSP
>eef_3_O.tauris
--EPYLIELLDVAFMLAGDDVRAAGDKAVDAIAPKLSE
>eef_3a_S.pombe
-SEVVFLPYLPTILDSFSDVVRQAAKMAAQALLDCLPA
>eef_3_P.guilliermondii
-QEAYLVQFFKTCFDSFADGCTRAAKQAADALYGDFPV
>eef_3_K.lactis
-QEIFFLPLLPVALDATIDTVKRAVQHAIDSIFVAFPI
>eef_3_L.elongisporus
-KEFFLLQFFATAYDMFTDNVVKAAKSATDALYSAYPV
>eef_3_C.cinerea
---PLLLPSLPVLYDLLMDVVRSAASAAIKSILKLCPP
>eef_3_D.hansenii
-SEAYLLQFFSICYDMCADNCVKAAKTAADSLYGIFPV
>eef_4_S.cerevisiae 3.7
VAIKALLPHLTNAIVETNKQEKIAILAAFSAMVDAAKD
>eef_4_C.albicans
TAVKAILPKLIDNLTNTNKTEKVAILRAVSQLVDTAKA
>eef_4_Y.lypolytica
RSVKFILPLIIESLVNTNKPEKVAALECISILVTVARD
>eef_4_N.crassa
NAVKATLNPLIDTIRNAQKPEKMTALDFIDTLIRTAPV
>eef_4_S.pombe
YAVKAIVPSVLESIHTTGKNEKMNSLQLLDVLVEVAPS
>eef_4_U.maydis
WAAQQVLKVVLHQARTAGKQVKTGCVALLEEMVTACPE
>eef_4_O.tauris
FAVRPALKPIFAGFQ-SQFQATMAALRVLDGFVARNRK
>eef_4_C.neoformans
WASFVLLPALLNLIRTSGKQIKAGSLEILQQLITSAPY
>eef_4a_S.pombe
AVETRLIPSLISYLDDSSIPSKVAALQLLGSLASSSPK
>eef_4_P.guilliermondii
ALGTVVLDELLNYLSSSAKNSKLGALGYFEKLIDDVPA
>eef_4_K.lactis
AQTSVLLPELLKYLTGSAKQCKLEALKLVDRIREESPN
>eef_4_L.elongisporus
ALGSVVLDEFLTFFKSGAKNSKAAALVNFDRIVEDVPA
>eef_4_C.cinerea
ESVPVVFRQLETILDTAK-RSKVGVLDGLKSFVASSRD
>eef_4_D.hansenii
AAGSIVINTLLSYLSSSAKNSKMAALDTFDKLIDDVPA
>eef_4_A.niger
AKVNALLPAISSYLSRGTGQGFVGAYCLIEKMALDAQM
>eef_5_S.cerevisiae 0.12
LRMPELIPVLSETMWDTKKEVKAAATAAMTKATETVDN
>eef_5_C.albicans
LRMPELIPVLSESMWDTKKEVKEAATATMTKSTETIDN
>eef_5_Y.lypolytica
LRMPELIPVLSEAMWDTKKEVKEQATATITKSTDTIDN
>eef_5_N.crassa
LLVPDLIPVISEAMWDTKKEVKERAYQTMEKLCQLIVN
>eef_5_S.pombe
YSLPQIIPVVSESMWDTKAEVKKQSKETMTKVCTLIAN
>eef_5_U.maydis
ALMPEIIPVMTEVIWDTKTDVQKASRAALTKLCALISN
>eef_5_O.tauris
ANLPEIIPELAQVMVHMRSEVKEASTASMAKVADCVGN
>eef_5_C.neoformans
EAMPDLVPVLAGAVWDTKSDVKKAAKATLEKAVSLVEN

>eef_5a_S.pombe
DYMAALIPCIKERMHDTKPEISRAAITCMLNLCSVVEN
>eef_5_P.guilliermondii
MTFIRSVPILTDLSTDFKPELSKRGLVVLKKFVKVLDN
>eef_5_K.lactis
LSFKSTVPILTDMATDFKPELAKQGYKTLLDYVKILDN
>eef_5_L.elongisporus
LKFVDTVPVLTDLSTDFKPELAKAGLQSLKKFVKVLDN
>eef_5_C.cinerea
SELGKVLPHVEKAMHDTKSEVSSAATKCATALCTTLAN
>eef_5_D.hansenii
LKFVNTVPVLTDLSTDFKPELAKHGLKVMMKFVKVLDS
>eef_5_A.niger
KTLKDLIPLVESGMHDLKNDVVKCACKAMNALTTLLSN
>eef_6_S.cerevisiae 64
KDIERFIPSLIQCIADPT--EVPETVHLLGATTFVAEV
>eef_6_C.albicans
KDIEKFIPQLISCIAKPT--EVPETVHLLGATTFVSEV
>eef_6_Y.lypolytica
KDIIKFIPALIACISKPT--EVPETVHILGATTFVSEV
>eef_6_N.crassa
RDIERFIPELIKCIAKPE--NVPETVHLLGATTFVTEV
>eef_6_S.pombe
ADIDRFIPELINCIAHPE--EVPETIHSLGATTFVTEV
>eef_6_U.maydis
KDIERFIPALINSLIHPV--EVPKTIQLLSATTFVQEV
>eef_6_O.tauris
LDIEPFIPTLIECINNVD--EVPECVHKLAATTFVQQV
>eef_6_C.neoformans
KDIEKFVPALVKSLLNPI--EVPKTISLLSATTFVSEV
>eef_6a_S.pombe
NDIIPHIPKLVDCMAHPE--TLEACIKDLSATTFVATV
>eef_6_P.guilliermondii
LDLQNKYDLIVDTLANPS--KVPECIKNLSSVTFVAEV
>eef_6_K.lactis
LDLQPRYDLIVDTLQDPQ--KVPDCVKSLSSVTFVAEV
>eef_6_L.elongisporus
LDLQNKYDLIVDTLADPQ--KVTDCIKNLSSVTFVAEV
>eef_6_C.cinerea
PDLTAHIPALVKCMAEPG--SVPACIKSLSNTTFVAEV
>eef_6_D.hansenii
LDLQNKFELIVETLSDPK--KVPECIKNLSSVTFVAEV
>eef_6_A.niger
DDVEPRIPLLIKTMEQPSE-TLQKAIHALSQTTFVAIV
>eef_7_S.cerevisiae 38
ATLSIMVPLLSRGLNERET-IKRKSAVIIDNMCKLVED
>eef_7_C.albicans
ATLSIMAPLLSRGLAERDT-IKRKAAVIVDNMCKLVED
>eef_7_Y.lypolytica
ATLSIMAPLLSRGLAERDT-IKRKAAVIVDNMCKLVDD
>eef_7_N.crassa
PTLALMVPLLDRGLNERDT-IKRKAAVIVDNMCKLVDD
>eef_7_S.pombe
PTLSIMVPLLARGLNERST-IKRKTAVIIDNMSKLVED
>eef_7_U.maydis
ATLALMVPLLSRGLNERPT-TKRKVAVIIDNMTKLVDN
>eef_7_O.tauris
PTLSIMGPLLQRGLFFQQS-IKRKSAVIIDNMCKLVED
>eef_7_C.neoformans
PTISLIAPLLIRGLDERPT-TKRKVCVIADNMSKLVDS
>eef_7a_S.pombe
VALAVLVPILKRALAQRSQ-MLRLTVIITDNLCKLVPD

>eef_7_P.guilliermondii
PALSLLVPILDKSLKMSSSDQLRQTVTVTENLTRLVNN
>eef_7_K.lactis
PALSLLCPILTRSLNLSSSEQLRQTVIVIENLTRLVNN
>eef_7_L.elongisporus
PALSLLVPILDKSLKMSSSEQLRQTVMVTENLTRLVNN
>eef_7_C.cinerea
PALAVLVPLLQRALNDRSM-VQRRTVIVIDNLVKLVRD
>eef_7_D.hansenii
PVLSLLVPILDKSLNMSSTDQLRQTVTVTENLTRLVNN
>eef_7_A.niger
PVLALLTPLLERSLNAPTTETLRQTVVVVENLTKLVHD
>tip2_H.sapiens 0.38
DSERKVVKMILKLLEDKNG-VQNLAVKCLGPLVSKVKE
>tip2_X.laevis
DSERKVVKMILKLLEDKNG-VQNLAVKCLGPLVSKVKE
>tip2_D.rerio
DSERKVVKMILKLLEDKNG-VQNLAVKCLGPLVSKVKE
>tip2_A.mellifera
DSERKVVKMLLKLLEDKNG-VQNLAVKCLGPLVNKVKE
>tip2_D.melanogaster
ESEKKVVRMVLKLLEDKNG-VQNLAVKCLGPLVNKVKE
>tip2_D.discoideum
TYENKIVTKLLALTADSAN-VQENVVKCLGLLIKRVKD
>tip2_C.elegans
DSTAKVIRALIKLLSDSNG-VQNLAIKCIGLLAQPSKI
>tip2_U.maydis
NTENNTLRQVLALMKDSNT-VKNMVVRCLAILVPRLRE
>tip2_C.neoformans
YTESQLVEMVLKLLADTNG-VKSAAVACISLMVKKPRP
>tip2_N.crassa
NTSNRTLDEVIKALSDQNG-VQNQAIKVLGPLVKKLPS
>tip2_Y.lipolytica
RLVDRMLTAVIEKLNDPIS-VQNQSVRCLEVLIRQVSE
>tip2_S.pombe
ESFPDTLDVLLQALSDASP-VQQEAVRCVAIISSKIPQ
>tip2_O.tauri
EQEREAMVAVTRCVFDASA-VAGLAMKCCASIAKRAAG
>tip2_A.thaliana
DLEVRLSSIILQQLDDVAG-VSGLAVKCLAPLVKKVGE
>tip2_O.sativa
DIEPKLTTTVLQQLEDASG-VSGLAVKCLAPLVKKVGE
>tip3_H.sapiens 13
YQVETIVDTLCTNMLSDKEQLRDISSIGLKTVIGELPP
>tip3_X.laevis
YQVETIVDTLCTNMLSDKEQLRDISSIGLKTVIGELPP
>tip3_D.rerio
YQVETIVDTLCTNMLSDKEQLRDISSIGLKTVIGELPP
>tip3_A.mellifera
YQVETIVDALCINMVSDKEQLRDISSIGLKTVISELPL
>tip3_D.melanogaster
IQVETIVDSLCANMMSNTEQLRDISSIGLKTVIAELPQ
>tip3_D.discoideum
SQATEIIDTLSKNILEESNELVEISGIGLKTIITNLPS
>tip3_C.elegans
HHLEYLVEELTPHVFSKAEQSRDIHSLTLKAMILNLAP
>tip3_U.maydis
KNMQHVQDTLIEYISVNNDELRDIAALALKTVTANMPA
>tip3_C.neoformans
SSLSKVINSLLEDVSSDNDERRDTSCLALKNVVLEMPL
>tip3_N.crassa
PLYSTAMQKLIELQSHNSD-VNSVPAIALKAVVEALPR

>tip3_Y.lipolytica
TQINFVIQTLIDSDSHSVGIESNITCTALKAIVGKLEA
>tip3_S.pombe
DKLKSTVENLLSGVAGKKS-KNYLSALSLLLSNSNVQP
>tip3_O.tauri
ETCEELCEELCKALSGKDG-RRDAASMCLKTIVMDIGT
>tip3_A.thaliana
ERIVEMTNKLCDKLLHGKDQHRDTASIALRTVVAQIAP
>tip3_O.sativa
DRVVEMTNILCDKLLNGKDQHRDTASIALKTIIVEVTT
>tip4_H.sapiens 8.1
NVCKKITGRLTSAIAKQEDSVQLEALDIMADMLSRQGG
>tip4_X.laevis
NVCKKITGRLTSAIAKQEDSVQLEALDIMADMLSRQGG
>tip4_D.rerio
SVCKKITGRLTSAIAKQEDSVQLEALDIMADMLCRQGG
>tip4_A.mellifera
NVCKRITGRLSSAIEKQEDSVQLEALDIMADLLSRFGA
>tip4_D.melanogaster
NVCQRITGKLSTAIEKEDVSVKLESLDILADLLSRFGE
>tip4_D.discoideum
LVIKNLVPKLLIGIDSEKLEIKMSCLDILNDLLQKYGS
>tip4_C.elegans
TVVKRMLPKFVDSLSLCAPAARVDVLDLIGEVLLRFGD
>tip4_U.maydis
IALNKLTPKLLNHVADASAELLIDSLDILAELITKFAA
>tip4_C.neoformans
SDIERIVTRVFKLFTNEIHQIASELLQILTDLFVRFSP
>tip4_N.crassa
LLIPRFLGRTAGKQVPGLLQVTSDSVDVLIEVVRCFGP
>tip4_Y.lipolytica
---ARLLARLLIPWMTKPVASSVDIVDIGIDFVRRFGS
>tip4_S.pombe
-TVFPSFLQILKQYNVAQ-EEFFAILCVVCDSLEIYHS
>tip4_O.tauri
-ACAPALAALVEKGGRDGANVAAEAVDVVHAIATALST
>tip4_A.thaliana
SILVTLTPQMIGGISGQGMGIKCECLEIMCDVVQKYGS
>tip4_O.sativa
KILVSLAPQLIKGATAGKSEVKCECLDILGDVLHRFGN
>tip5_H.sapiens 0.16
NFHPSILTCLLPQLTSPRLAVRKRTIIALGHLVMSCGN
>tip5_X.laevis
NFHPSILSCLLPQLTSPRLAVRKRTIIALGHLVMSCGN
>tip5_D.rerio
NFHPSILSCLLPQLTSPRLAVRKRTIIALGHLVMSCGN
>tip5_A.mellifera
TFHSTILAALLPQLSSPRQAVRKRTIIALSHLLTSSNN
>tip5_D.melanogaster
PFHSTILKALMPQLASSRQAVRKRTIVALSFLLIQANS
>tip5_D.discoideum
GDLENIQKVVLPKLNATRPAIRKRAILCLANIAFPSPD
>tip5_C.elegans
EMHKGSLKVMVDHLYSFRSAIRKKAITGIGHLASVING
>tip5_U.maydis
SLQNALLKSVVPAMRHSRPAVRKRSLTVLGALGPCATS
>tip5_C.neoformans
AIQSTALSSLIQILDNARPAIRKRAVPTLSSLIATSPQ
>tip5_N.crassa
VEIEALHDAVLNLLAQEKCVVKKRAVAAVSMLAHYLSD
>tip5_Y.lipolytica
EEIATLKTHFVGLLKSPEPMIQKRAVLGLSALARYLST

```
>tip5_S.pombe
LNNFELCIDVFQKCTTQCQLIIKKACYLLSDVSLYGPR
>tip5_O.tauri
ETSDELQRTLLGHVERGKTGTRKRAAQCVALLSTYAKD
>tip5_A.thaliana
DDHEKLLNTLLLQLGCNQATVRKKTVTCIASLASSLSD
>tip5_O.sativa
KDHDSMLTALLSQLSSNQASVRKKSISCIASLAACLSD
>tip6_H.sapiens                                  2.7
IVFVDLIEHLLSELSKNDSSTTRTYIQCIAAISRQAGH
>tip6_X.laevis
IVFIDLIEHLLTELSKNDSSTTRTYIQCIAAISRQAGH
>tip6_D.rerio
LVFVDLIEHLLSELSRNESSTTRTYIQCIAAISRQAGH
>tip6_A.mellifera
YLYNKLVDHLLEGLYTQTANVIRTYIQCIASICRQAGH
>tip6_D.melanogaster
NAYNGVIDHLLDGLENPPNAAIRTYIQCLASICRQAGH
>tip6_D.discoideum
NLFNSLLDYIIKSIEEAKKDHISTLIQAIGAICKSSGY
>tip6_C.elegans
ELYDELVQDLLKELAQRSPVQLRTLVIALSTVARASGS
>tip6_U.maydis
DIFTQLSVQLSSDLAPKNVDTRKTAVQLIAIFARTCPR
>tip6_C.neoformans
LFNEDLEKEIVNGVGQGGE--SRIWMGTVASLARGKSA
>tip6_N.crassa
DLLAASVKRTVALLRKSSMATRRLYITILGSMARSIPH
>tip6_Y.lipolytica
PEFDSLVQHCIGMLPKQP----KIVVTLIGSLVSGEPA
>tip6_S.pombe
FAYKYIIEVLDRGLGPSTQNISIKLLNEILLSSKKEKD
>tip6_O.tauri
DALDRTVETVSTSLEESIAGKSDLYAFTLGAVARAVGY
>tip6_A.thaliana
DLLAKATVEVVKNLSNRNAEITRTNIQMIGALCRAVGY
>tip6_O.sativa
DLLAKATFEVVQLLKNRSAEIARTNIQMIGALSRSVGY
>tip7_H.sapiens                                  0.03
EYLEKIIPLVVKFCNVDDDELREYCIQAFESFVRRCPK
>tip7_X.laevis
EYLEKIIPLVVKFCNIDDDELREYCIQAFESFVRRCPK
>tip7_D.rerio
EYLEKIIPLVVKFCNVDDDELREYCIQAFESFVRRCPK
>tip7_A.mellifera
EQIEKVMPLIVQYSNEDDDELREYCLQAFESFVYRCPK
>tip7_D.melanogaster
NHIDRSMLLLSQYSQRDDDELREFCLQACEAFVMRCPD
>tip7_D.discoideum
KYLPKVMPHVLNYCDNNKFELRENCLLCFEAIIEKCQK
>tip7_C.elegans
KHTPKVVPFLLQYLQIDPGDLREASIQGLEVFLYRNPQ
>tip7_U.maydis
RRLPEFMPTIIETAKYDDDELRETCLQSIELILLRCPA
>tip7_C.neoformans
VNEGKLAELILSQTKNVEDETVEAALTALEALVLRCPS
>tip7_N.crassa
RYLPEVVSFVLDALNEEELDVREAALVALDAFLSSCPN
>tip7_Y.lipolytica
PYAPATFPLVFDCLQLDLEELREVTLYSLEGFLKLPQA
>tip7_S.pombe
TAVADYTNKILSLLKKEEAELTQKLLEVLGLLLEYQQV
```

>tip7_O.tauri
EHAERVTLILLRVCKSATDVNIESALRAIESIVSSCSS
>tip7_A.thaliana
THLGNTVPVLINYCTSASEELREYSLQALESFLLRCPR
>tip7_O.sativa
PHLAEAVPLLINYCTSASEELREYSLQALESFMLRCPR
>tip_8_H.sapiens 4.3
KEVYPHVSTIINICLKYLTKVRRAAAKCLDAVVSTRHE
>tip_8_X.laevis
KEVYPHVSTIINICLKYLTKVRRAAAKCLDAVVSTRHE
>tip_8_D.rerio
KEVYPHVPTVISICLKYLTKVRRAAAKCLDAVVSTRHE
>tip_8_A.mellifera
KEITPHINKIIEICLVYITKVRRAAAKCLEAVVSSRRE
>tip_8_D.melanogaster
DAINPHIPMILELCLNYITKVRRAAAKCLEVLISTRQE
>tip_8_D.discoideum
KDVTPYIGEIITLCTKYIKKIRRSSCKTLCAIISTRPE
>tip_8_C.elegans
QEVVAFEKEVIQQLTDALAKVRRAAAKAIEAMISSHRE
>tip_8_U.maydis
AEVTPFVNASIDLAITLIKKVRRASAKVLNAALTSRPE
>tip_8_C.neoformans
SEMFPYISAITQRSLVLVKKIRRSSAKLLRALISTRPD
>tip_8_N.crassa
NQMRPYTNDAIEACLRYLKKVRRCAAKALHTIISTRSS
>tip_8_Y.lipolytica
AIVTTFVEKLVEASRKFIVKLRRYAAKLADSIVKNCAQ
>tip_8_S.pombe
VNILKIWPELHGLLISKISIVRRESLKVVLSVILSRLE
>tip_8_O.tauri
TQGSEGTAARIAVALKYVSKVRRAAAKVLSSVMSTAPE
>tip_8_A.thaliana
RDISPYCDEILNLTLEYISKVRRAAAKCLAGLIVSRSE
>tip_8_O.sativa
RDISPYCEGILNLALEYISKVRRASAKCLSAIIVSRPE
>tip_9_H.sapiens 0.36
EFYKTVSPALISRFKEREENVKADVFHAYLSLLKQTRP
>tip_9_X.laevis
EFYKTVSPALISRFKEREENVKADVFHAYLSLLKQTRP
>tip_9_D.rerio
EFYRTVSPALIARFKEREENVKADVFHAYLSLLKQTRP
>tip_9_A.mellifera
ELYKIVSPALILRFKEREENVKSDIFHAYIALLRQTRP
>tip_9_D.melanogaster
DFYRSLSPALIARFKEREENVKSDIFHAYVALLKNTRL
>tip_9_D.discoideum
ELYQKVAPVLYNRFKEREENVRLDIFTTFVLLLKQLNK
>tip_9_C.elegans
-LSQKIGPVVIGRFKEREETVRTEIISVYIALLNQISI
>tip_9_U.maydis
QNVGNVAPILVTRFSEREESVRLEILDTFLALLKQMQL
>tip_9_C.neoformans
ELYNSATPVLISRFAEREESVRLEVLAAFEMLLKQTAT
>tip_9_N.crassa
LLYQKVAPALVKRFDEREENVRLEVLSAVSLLIRKTGE
>tip_9_Y.lipolytica
LVYASLYPCILKRLQERETTVVVALLDTLGSIVVYSGV
>tip_9_S.pombe
IVLQALGTSVVSKLNDREESVCLISIEVLKQAFLHVPR
>tip_9_O.tauri
EHYDDVMSKLLSRSRDREPSVQLDIFSVIGDVIHVTRR

>tip_9_A.thaliana
KVYQEACPKLIDRFKEREENVKMDVFNTFIDLLRQTGN
>tip_9_O.sativa
KMYLEACPKLIERFREREENVKMDIFNTFIELLRQTGN
>tip_10_H.sapiens 18
SQVPNIVKALHKQMKEKSVKTRQCCFNMLTELVNVLPG
>tip_10_X.laevis
SQVPNIVKALHKQMKEKSIKTRQCCFNMLTELVYVLPG
>tip_10_D.rerio
SQVSMIVKALHKQMKEKSVKTRQCCFNMLTELVNVLPG
>tip_10_A.mellifera
QQVPLIVKAVHRQMKEKSIKTRQDCFSLLKELVLVLPG
>tip_10_D.melanogaster
EQLPLIVKAIQPLMREKSMKTRQDCFLLLRELLNSLPG
>tip_10_D.discoideum
QQVPKLVQSISKSLIDKSIRTRVGAIALLKELVMIIPG
>tip_10_C.elegans
DQKDVLLRTITKSMK-KHPKTGPKCIELLSALIRTYPS
>tip_10_U.maydis
VLVPAIAKALNREIISKSIPTRHKSFVVLRELVIVLHG
>tip_10_C.neoformans
SYLPQLSKAILTQLSSKSVPTRQQSFSLLRQAATALGG
>tip_10_N.crassa
ALTPAIVKSATRLLKGKLIPTKQATISILDDLISVQKG
>tip_10_Y.lipolytica
ATRTKLIQVTSKWLTQQA--TAPLAAFVYRSLAQTLEA
>tip_10_S.pombe
DTSIYLVSVIGKHVSKLSDKTPLSIVSELLNLVTVIFS
>tip_10_O.tauri
ASATDVVRVIVRESTSKNQKTQIAAYTLLRSLGDVFPG
>tip_10_A.thaliana
QEVSKIVKSINRQLREKSVKTKVGAFSVLRELVVVLPD
>tip_10_O.sativa
QEVPKVVKSINRQLREKSIKTKVGAFSVLKELVVVLPD
>tip_11_H.sapiens 2.9
QHIPVLVPGIIFSLNDKSSNLKIDALSCLYVILCNHSP
>tip_11_X.laevis
QHIPVLVPGIIFSLNDKSSNLKIDALSCLYVILCNHSP
>tip_11_D.rerio
QHIPVLIPGIIFSLNDKSSNLKIDALSCLYVILCNHQP
>tip_11_A.mellifera
NHIPALIPGIQYSLGDKNSNMKIDTLAFVHTLLITHQP
>tip_11_D.melanogaster
PYLDSIVPGISYSLNDKSSNMKIESLGFLYSLLQGHPP
>tip_11_D.discoideum
GQVSQIVNGINLSLSEKNTNLKIEALVLLKLLLINEPA
>tip_11_C.elegans
DSLDDIIPAVSNILTDKNAQGKMTVLSFISNALTLNNP
>tip_11_U.maydis
AHIGTILAHTEKALKGAESNLKADVLGFFRVLILTHAP
>tip_11_C.neoformans
DSADPICTAAASALRTIDSSLAIATLSFLTVFFDTHSA
>tip_11_N.crassa
PYLDQITDLILDTIKVTGSTLRIAALRLISSFARNHSS
>tip_11_Y.lipolytica
AHVLEVIPVLHNAVIDGKHTLLADALEISQIAVSKTDW
>tip_11_S.pombe
LGVQSEFSNLSSIIYRFPDKIKLNLVRLISAIISCGCE
>tip_11_O.tauri
EVRDTEARDSVVRAVERCIAARIEALAFICSICKPEGF
>tip_11_A.thaliana
DHIGSLVPGIERALNDKSSNLKIEALVFTKLVLASHAP

```
>tip_11_O.sativa
DHIGSLVPGIEKALNDKSSNLKIEALVFTRLVMASHSP
>tip_12_H.sapiens                                1.9
PHVQALVPPVVACVGDPFYKITSEALLVTQQLVKVIRP
>tip_12_X.laevis
PHVQALVSPVVICVSDPFYKITSEALLVMQQLVKVIRP
>tip_12_D.rerio
PHVQAIVPPVVACVGDPFYKITSEALLVTQQLVKVIRP
>tip_12_A.mellifera
AHMAVLAPPIITAVGDPFYKITAEALLVLQQLVQVIRP
>tip_12_D.melanogaster
PHIPLLVPLVVTSVFDPFYKIATEALLVLQQLVKVIRP
>tip_12_D.discoideum
SHITSLSTHIVKCINDSYYRIASEALRVCQEFVIVFNK
>tip_12_C.elegans
NLLSPLTTIMTHSISEPFYKVSAEGLAVCCKYIDVLRE
>tip_12_U.maydis
DQLPGLVPILAASIEDKLHRSCVEAFLTCSQLVSVLRP
>tip_12_C.neoformans
SHLGNLVPAIVRCMKDKLQRISFEAFDTASALTKSLRP
>tip_12_N.crassa
PYLPKIVDRVVSVVHDRVYKIAAEAVQTAEEVSKAITP
>tip_12_Y.lipolytica
QYLAQIQDLIQAGLKDTFFKVPVAAIDLVMCLMGVNPE
>tip_12_S.pombe
NMESKMSTILSLAVQNNYPQLSYEALITELSFCKYIHK
>tip_12_O.tauri
PYVHGLLPHIYTACADKYYKIVAESLRSCAALVFVLRR
>tip_12_A.thaliana
PYIKALSSPVLAAVGERYYKVTAEALRVCGELVRVVRP
>tip_12_O.sativa
PYIQALSGPILSAIGDRYYKVTAEALRVCGELVRVLRP
>tip_13_H.sapiens                                1.9
PYIKDLFTCTIKRLKAADIEVKERAISCMGQIICNLGD
>tip_13_X.laevis
PYIKDLFTCTIKRLKAADIEVKERAISCMGQIICSLGD
>tip_13_D.rerio
PYITDLFACTIKRLKAADIEVKERAISCMGQIICNLGD
>tip_13_A.mellifera
SLSGEIYRCTLMRLRTADIEVKERAIACMGQILAHFGD
>tip_13_D.melanogaster
SFVGQVYSCTLQKLKVTDVEVKERAIACMGQIIANMGD
>tip_13_D.discoideum
PIISNLFAANFVQLKAQDIEVKEAAISSIGTIITLFGN
>tip_13_C.elegans
EEAKKLLVVVEKKFMANDTEVRERAISAISMLLAAFKD
>tip_13_U.maydis
TYLLQIYAATVARLNRLDSEIKERGIACLGVLLAHAGD
>tip_13_C.neoformans
TPIQEVFVATTEVLGDNSVDVREKALATLGSILVQSGD
>tip_13_N.crassa
GELQKLYSVIVDRTTDNDAEVRQKAIHALGTLLSRTSG
>tip_13_Y.lipolytica
PR-DAIEALILDFASSTAIEIRVRAISCLGKLNDGQES
>tip_13_S.pombe
TDFSTMIDSSLQLLESKISKVRLALIDLVSQYVILFYE
>tip_13_O.tauri
TEIKSLLDAVLSKLDSSDEDVKEAAIHVCAVILAKLND
>tip_13_A.thaliana
PFVHPIYNAIMSRLTNQDQEVKECAITCMGLVISTFGD
>tip_13_O.sativa
PYIGPIYKAILARLANQDQEVKECAISCMSLVVFTFGD
```

>tip_14_H.sapiens 23
SDLPNTLQIFLERLKNEI--TRLTTVKALTLIAGSPLK
>tip_14_X.laevis
SDLPSTLQIFLERLKNEI--TRLTTVKALSLIAGSPLK
>tip_14_D.rerio
ADLPGTLHIFLERLKNEI--TRLTTVKALTLIAGSPLK
>tip_14_A.mellifera
DELHICLPIFLDRLRNEI--TRLTTVKALTCIAASPLR
>tip_14_D.melanogaster
NELAVCLPIFMERLKNEV--TRLSSVKALTLIAASSLR
>tip_14_D.discoideum
SELQPCLSILLERLDNEL--TRVVTVKVLSRIINSSIN
>tip_14_C.elegans
NETPAILEKMTERIGRDM--TCLVAFRASTHIVEAGII
>tip_14_U.maydis
EKHRECFDLLTARLTNEV--TRFITVKVITQIAASPIC
>tip_14_C.neoformans
SSFSTSLPLITNRLGSES--TASTAIVVIGQLAASSQC
>tip_14_N.crassa
VDRKTALGHLKERLFNET--TRLAAVRAIDTVAAFSSS
>tip_14_Y.lipolytica
NDGQESHAILLKSLENET--FRLAAIGAVRDLVERDRV
>tip_14_S.pombe
IFLRRVLIILCKKLQEEP--TRSAAARALCDIFMSVTD
>tip_14_O.tauri
QDQSRVLGLLLERSRNET--TRLAAVRAFAMIAGSSSA
>tip_14_A.thaliana
AELPSCLPVLVDRMGNEI--TRLTAVKAFSVIATSPLH
>tip_14_O.sativa
RELPACLPILVDRMGNEI--TRLTAVKAFAVIAKSPLR
>tip_15_H.sapiens 1.7
PVLGEGVPILASFLRKNQRALKLGTLSALDILIKNYSD
>tip_15_X.laevis
PILGEGVPILASFLRKNQRALKLGTLSALDILIKNYSD
>tip_15_D.rerio
PILGEAVPILASFLRKNQRALKLSTLAALDILVKNYSD
>tip_15_A.mellifera
QIMEEAIPILGSFLRKNQRALKLCSLPLLDTLVRNYSS
>tip_15_D.melanogaster
PILHDVLPALGTFLRKNHRALKLHSLDLINKIVINYSS
>tip_15_D.discoideum
SILPSAIKLLSTFLRKNNRVLKQSSLIALNDIVKVCPN
>tip_15_C.elegans
AQLQSILRHVVDYVKKIARSLRMTCLNFVEKLMKHSPA
>tip_15_U.maydis
AFLRDSISEVATLLRKSNRQLRLAAFDCLTAALSRSST
>tip_15_C.neoformans
GWLLQILPEVVVALRRTKRTSKNAEFTCLLNIIERVGK
>tip_15_N.crassa
PWTQEVVVELAAQLRKANRSLRGSSVMALKHLVLSPAT
>tip_15_Y.lipolytica
EFASHVVSQLVTLLRQSQRLLRLSALETLKEMSASSVV
>tip_15_S.pombe
KIYEEILQDCCRHIDKSGNEFTTAYLELLEVLLKVGQK
>tip_15_O.tauri
AVAASVTGEFTTFLRKSNKALRESSLAALTALVSCHSA
>tip_15_A.thaliana
CVLDHLIAELTGFLRKANRVLRQATLITMNTLVTAYGD
>tip_15_O.sativa
CVLDHVISELTAFLRKANRALRQATLGTLNSLVVAYGG
>tip_16_H.sapiens 27
AMIDAVLDELPPLISESDMHVSQMAISFLTTLAKVYPS

>tip_16_X.laevis
AMIDAVLDELPPLISESDMHVSQMAISFLTTLAKVYPS
>tip_16_D.rerio
AMIDAVLAELPPLINESDMHVSQMAISFLTTLARVHPD
>tip_16_A.mellifera
DLLDKVTTELPALLNETDLHIAQLTLNLLTTIAKLHPV
>tip_16_D.melanogaster
NLLQTAIVEIPPLISDSDLHVAQYSLTLLSTVARRQPQ
>tip_16_D.discoideum
SLLTGILTEMATLINESDLQITHLAFVFIQNLLKNYSE
>tip_16_C.elegans
EELTCVLGEMSNLISETDLQITNQAFCCLTYAFLNFPT
>tip_16_U.maydis
ASSNLILAEVQPLINTDMDNLLPHILRSVNLILLNDPA
>tip_16_C.neoformans
DLAEGLIIELTPVIDTPMAQTIALALTHQPSARPTVDA
>tip_16_N.crassa
VTVQKVVTALVTVITHYDAQLLGPGLLVLARLAQEKPQ
>tip_16_Y.lipolytica
---HVISSFLNNGSRDIDDQLVALEIDVIANLFSLVSS
>tip_16_S.pombe
SLLEHILGLLIETLKRNTETVAILKCLLIIPLSILLKS
>tip_16_O.tauri
PDVLPVVTESSSLLNEEDLHLATMSAGLLSAIAAAASS
>tip_16_A.thaliana
EAYEVILVELSSLISVSDLHMTALALELCCTLMTGKSC
>tip_16_O.sativa
S-YETIIAELSTLISDMDLHMTALALELCCTIMVDRKS
>tip_17_H.sapiens 120
KISGSILNELIGLVRSPL--LQGGALSAMLDFFQALVV
>tip_17_X.laevis
KISGSILKELIGLVRSPL--LQGGALSAMLEFFQALVV
>tip_17_D.rerio
KISGSILAELIALVRSPL--LQGGALSAMLEFFQALVA
>tip_17_A.mellifera
RVSDNILPEILVLVKSPL--LQGVALNSMLEFFQALVQ
>tip_17_D.melanogaster
GIHEQFLRSVLILVRSPL--LQGSALNCTLELFQALVQ
>tip_17_D.discoideum
LVNEKCIPPTLALLKSSL--LQGVALESLLSLFATIVQ
>tip_17_C.elegans
LHMQPILDSIIRLLTSPL--IQGLALNSLLNLFTAIVK
>tip_17_U.maydis
PVRVSVLPHIYSVLSLPI--AQGLAMESLLEFLRLYAG
>tip_17_C.neoformans
TVDAQLYPKILTTLKTS---LNPHLVDALAEFFTAYAA
>tip_17_N.crassa
-ITEELMTALCKLLMEST--VTGTVLDSLLVLVNSIGQ
>tip_17_Y.lipolytica
PVIELPLNHIHVTSELL---LADSFVNFYAQITSLRTD
>tip_17_S.pombe
SHLQSSTIHLNEESVCLL--SRIIAVISKEEDLELIIN
>tip_17_O.tauri
SMATTTLPLALALTRSPL--VQRQTLKSLQELYKSLVL
>tip_17_A.thaliana
AVRNKVLPQALTLVKSPL--LQGQALLDLQKFFEALVY
>tip_17_O.sativa
AVRYKVLPQALILIRSAL--LQGQALQALQRFFASLVQ
>tip_18_H.sapiens no match
TNNLGYMDLLRMLTGPVYSQSYYSIAKCVAALTRACPK
>tip_18_X.laevis
TATLGYMDLLRMLTGPVYAQSYYSIAKCVAALTRACPK

>tip_18_D.rerio
TASLGYMDLLRMLTGPVYAQSYYSIAKCVAALTRACPK
>tip_18_A.mellifera
IPGLGYRELLSMLLAPVSQQAYHSLAKCAAALTITWHQ
>tip_18_D.melanogaster
LSGLDYHSLVSKLMAPVLGQAYHSSAKCIAALTQQCPQ
>tip_18_D.discoideum
EPGMKYEQLLTLLFNTAADQSFHSISQCIAVITVNTTP
>tip_18_C.elegans
PEKPTFESLLDSVTSPVYDHAHMAIASCAAVITESTQN
>tip_18_U.maydis
ASAPETVEELLAALEAAKGHVYSTVSRCIGAVCTVSDA
>tip_18_C.neoformans
NSPERAVQLVQELSNNLGSAAWGTTAKCVGGVVKGEIA
>tip_18_N.crassa
QGEVLMQRLLNEVALSGD---PSVVGKVIGTLLVASGN
>tip_18_Y.lipolytica
DLRHVIYEQLLRACDSGHK-HKGISTQIIGFVCAGDDQ
>tip_18_S.pombe
-EDLELIINSFTCAQKPV-EEMVTLALIAAQLICIFQS
>tip_18_O.tauri
VVTFKPLLDALSDTSGIQSFVAHSLAKCVASACVASGE
>tip_18_A.thaliana
NTSFYTLLESLLSCAKPSPQALYSIAQCVAVLCLAAGD
>tip_18_O.sativa
NTSFDTLLDSLISTAKPSQQALSSIAQCVAVLCLAAGD
>tip_19_H.sapiens 38
KEGPAVVGQFIQDVKNSRSSIRLLALLSLGEVGHHIDL
>tip_19_X.laevis
KEGPAVVGQFIQDVKNSRSSIRLLALLSLGEVGHHIDL
>tip_19_D.rerio
KEGPAVVGQFIQDVKNSRSSIRLLALLSLGEVGHHVDL
>tip_19_A.mellifera
QEAQAVVEQFLKDVQNPQSAQHIFALLVIGEIGRHVDL
>tip_19_D.melanogaster
QVATPLATKLITDLQKRNDTEIIFCLLTIGEIGRHFDL
>tip_19_D.discoideum
NTTPALRKQTIHNLICNLSELVLLSLSCLGEIGRRIDI
>tip_19_C.elegans
EKSRSLAKKLAQQLQTANMSIRLFAMITLGELGRRVPD
>tip_19_U.maydis
AASVNVAAQATATLESSSNTDVYFALLLLGELGRSNDF
>tip_19_C.neoformans
ASAGESLALFEDVVKGKNVTDAYLALLCIGEIGRIVNL
>tip_19_N.crassa
MKGTYTAELFVQEIQKQKG-ERASLALTILGEAGLRLG
>tip_19_Y.lipolytica
GDDQLTNTYISRASEAGGASSRASDLLILGYIGAAKAL
>tip_19_S.pombe
FQSKAIVTSLNKSFMSPKSRIKVFTTLIFGQLDYGKLT
>tip_19_O.tauri
ASGEAVTKETTDTLLAKLKGDAVYTLLCIGEIGRLTDV
>tip_19_A.thaliana
DKNCSSTVKMLMEILKDDSSKQHLALLSLGEIGRRKDL
>tip_19_O.sativa
DQKCASTIEMLKGILKDDSSKQHMALLCLGEIGRRKDL
>tip_20_H.sapiens 0.091
SGQLELKSVILEAFSSPSEEVKSAASYALGSISVGNLP
>tip_20_X.laevis
SGQLELKSVILEAFSSPSEEVKSAASYALGSISVGNLP
>tip_20_D.rerio
SSQPELKTVILDAFSSASEEVKSAASYALGSISVGNLP

>tip_20_A.mellifera
SGISSLKHIILNSFSSHSEEVKSAASYTLGNIAVGNLP
>tip_20_D.melanogaster
SSIQVLPQTIIECFGATSEDVKAAASHALGAVSVGSLQ
>tip_20_D.discoideum
HENENLQESVYKTFEANNEEIKQVAALCLGDIAVCSLQ
>tip_20_C.elegans
DFPVKPEDLAIKAFNHHHEDLKSAAAQALGALAVGNLN
>tip_20_U.maydis
STHAGLLDRVLRFYTADSEEVKMAAAFAVGNMAVGALA
>tip_20_C.neoformans
STKTDLFEIILSFFKHDSEEVRSAAAFAAGNLAVGVPD
>tip_20_N.crassa
DKFPYSPSLFLEQFHSEYDKTSLSAAVALGRAGAGNVA
>tip_20_Y.lipolytica
---PISPELFAPHLTGSDEVIRYASAVALGNCARANP-
>tip_20_S.pombe
LPANEYFDTIASNLNSPNADVMKAAAIALGSLTSQSE-
>tip_20_O.tauri
SLNKELETILFSAFDSYGDDVKGAAALTLGRVAVGNRE
>tip_20_A.thaliana
SAHAGIETIVIESFQSPFEEIKSAASYALGNIAVGNLS
>tip_20_O.sativa
SNHAQIENIVIESFQSPFEEIKSAASYALGNIAVGNLS
>tip_21_H.sapiens 290
GNLPEYLPFVLQEITSQPK-RQYLLLHSLKEIISSASV
>tip_21_X.laevis
GNLPEYLPFVLQEITSQPK-RQYLLLHSLKEIISSASV
>tip_21_D.rerio
GNLPEYLPFVLQEISGQPK-RQYLLLHSLKEIISSASV
>tip_21_A.mellifera
GNLPKYLPFILKEIEAQPK-RQYLLLHSLKEIITCQSA
>tip_21_D.melanogaster
GSLQTYLPLILHEIEVQPK-RQYLLLHSLKEVISSLSV
>tip_21_D.discoideum
CSLQSYLPFILEQIKNQPK-KQYLLLHTLRETIVKLSH
>tip_21_C.elegans
GNLNVYLPFILEQIRTQPK-KQYLLLHALKEVIVWESS
>tip_21_U.maydis
GALAVFLPAIEQHVRARSD-HRFLSLHALKELITHGSG
>tip_21_C.neoformans
GVPDVYVPAIITRISAAKD-ERLLLLHAIKEVILHSPS
>tip_21_N.crassa
GNVAAYVPVILQSMHQGGN-TQYLLLQSIKEVLQQVAM
>tip_21_Y.lipolytica
-ANPNFLAYTLSLLNQ----VTYLALVALREYIKSSTS
>tip_21_S.pombe
-QSEKFIKELCALYVSDAY-DKELLLISFLTFLKKSKI
>tip_21_O.tauri
GNREKYLPLITSKLANENI-HQYSLLQALREVIVVGNL
>tip_21_A.thaliana
GNLSNYLPFILDQIDNQQK-KQYILLHSLKEVIVRQSV
>tip_21_O.sativa
GNLSKYLPFILNQIDNQQK-KQYLLLHSLKEVIARQSV
>tip_22_H.sapiens 0.34
PYVENIWALLLKHCECAEEGTRNVVAECLGKLTLIDPE
>tip_22_X.laevis
PYVENIWALLLKHSECAEEGTRNVVAECLGKLTLIDPE
>tip_22_D.rerio
PYVENVWALLLKHCECTEEGTRNVVAECLGKLTLIDPE
>tip_22_A.mellifera
NFVPSIWMLLYRHCECTEEGTRNVVAECLGKLTLIDPA

>tip_22_D.melanogaster
PSVPSIWDQLFKHCECSEEGSRNVVAECLGKLVLVNPD
>tip_22_D.discoideum
PFLQSILPLLFDNCVNEEEGTRNIVAECLGKLSMIEPN
>tip_22_C.elegans
SAIVDIWGMLMANAGGNEDGTRSVVAECLGRLCSFDPE
>tip_22_U.maydis
VVADQVWPVLFEACETKEEGVRSIGAECLARLTLSEPI
>tip_22_C.neoformans
SLADTLWAPLFSATDATPAGIRNVIAACIGKLTTTVPA
>tip_22_N.crassa
ELSTPIWNQILSASGSE--DNKAVCAECIGRLVIIDPK
>tip_22_Y.lipolytica
SDPEPLFSRLFDISEMDD-NIQSVVSECVGRICVRHE-
>tip_22_S.pombe
ETADKIWDILSKDIENIKDPFRTLLSECLGLLICNESS
>tip_22_O.tauri
QEANEVMAILDRTASSEEEGVRNVVSECLGRLTASNPK
>tip_22_A.thaliana
SSVEKILALLFNHCESEEEGVRNVVAECLGKMALIEPE
>tip_22_O.sativa
SNIEKILALLFNHCESEEEGVRNVVAECLGKIALIEPR
>tip_23_H.sapiens 13
IDPETLLPRLKGYLISGSSYARSSVVTAVKFTISDHPQ
>tip_23_X.laevis
IDPETLLPRLKGYLAAGSSYARSSVVTAVKFTISDHPQ
>tip_23_D.rerio
IDPETLLPRLKGYLLSGSSYARSSVVTAVKFTISDHPQ
>tip_23_A.mellifera
IDPATLLPRLQESLKSTSALLRTTTVTAVKFTISDQPQ
>tip_23_D.melanogaster
VNPDELLPQLQQALRSESATMRTVVVSSVKFTISDQPQ
>tip_23_D.discoideum
IEPNEIIPKLVEKIKSPSPLERSTIVTSIKFSIMENKE
>tip_23_C.elegans
FDPESLLPKLKESMR-SSDAIRSSAVSAIKYMINDEKR
>tip_23_U.maydis
SEPIKFLALLQERLRSPSASVRATVLAAIRFTLSTESS
>tip_23_C.neoformans
TVPAKFLPQLQQLLYSSP-SNRAIVAAAVRYTFIDTSN
>tip_23_N.crassa
IDPKTYMSKLVFLLN-DPSLLRAIAIQALRYTLADENE
>tip_23_Y.lipolytica
VRHEAYVGDLKKRLGDPNSVTQAIVLSSLRYIFGQVSS
>tip_23_S.pombe
NESSSLYYKLELLSSSEASNHMLLSLSVFRFSLTLDCP
>tip_23_O.tauri
SNPKVLMPEIANRFAASASLEKATHISAVKFAVLASAK
>tip_23_A.thaliana
IEPEKLVPALQVRTTSPAAFTRATVVTAVKYSVVERPE
>tip_23_O.sativa
IEPRKLIPALKERTSSPAANTRATVAIAIKYSIVERPG
>tip_24_H.sapiens 0.039
PLLKNCIGDFLKTLEDPDLNVRRVALVTFNSAAHNKPS
>tip_24_X.laevis
PLLKNCIGDFLKTLEDPDLNVRRVTLVTFNSAAHNKPS
>tip_24_D.rerio
PLLKNCIGDFLKTLEDPDLNVRRVALVTFNSAAHNKPS
>tip_24_A.mellifera
VMLKQCMDNFLVALEDPDLNVRRVALVAFNSAAHNKPM
>tip_24_D.melanogaster
VLLKQNIGEFLFALRDPEPQVRRVALVAFNSAVHNKPS

```
>tip_24_D.discoideum
QYLAPNISQFLSLLHDGDLIVRRSALLSLNYIAHNKPN
>tip_24_C.elegans
ITLQKQIGDFLAAVRDEDLKVRRVALVVLNSAAHNKPA
>tip_24_U.maydis
ELLAPALVDFLALLSDPELEVRRNATFALNSAAHNKPY
>tip_24_C.neoformans
ELIAPIIVEFLSLMKDENLIVRRLSLASLNAALQNKPY
>tip_24_N.crassa
SMLKSHLVDMLKTMLEDPEENRRHAMSTLNSAAHNKAD
>tip_24_Y.lipolytica
EDLHSVPRDLVTLVDNVKQNTVELSLTCLNAILHNAPT
>tip_24_S.pombe
AYEKQFFEKAYKLFQNPDLEVSQETLQVIISVIKNRRS
>tip_24_O.tauri
IRGDLRLPEFMSAISDEDVNVRTAVIKMISAVIHRESA
>tip_24_A.thaliana
EIIFPQISSFLMLIKDGDRHVRRAAVSALSTFAHYKPN
>tip_24_O.sativa
EIMYSEISTFLMLIKDSDRHVRRAAVLALSTAAHNKPN
>tip_25_H.sapiens                                   0.17
SLIRDLLDTVLPHLYNETKDIRKAAFECMYTLLDSCLD
>tip_25_X.laevis
SLIRDLLDSVLPHLYNETKDIRKAAFECMYTLLDSCLD
>tip_25_D.rerio
SLIRDLLDTVLPHLYNETKDIRKAAFECMYTLLDSCLD
>tip_25_A.mellifera
MLIRDLLDSVLPHLYAETKDLRKAAFECMYTLLDSCLD
>tip_25_D.melanogaster
SLVRDLLPTLLPWLYSETKDIRKAAFECMYTLLEQGLD
>tip_25_D.discoideum
NLIRNDLSVYLPILYNNAKEIRKTAFECMYTLLDTSID
>tip_25_C.elegans
ALVRDLLPDLLPAVYEETKDLRKCAFECMFTLLESCVD
>tip_25_U.maydis
YLIRDHLVTLLPLLYAETHDLRKNAYETMYQLLDSLWS
>tip_25_C.neoformans
YLIVDKLDILQPLLYQETYENRKTAYETMYTLLGACFS
>tip_25_N.crassa
DFILGHLNKLMPYVMKETVELRKAAYETLYALMETAFS
>tip_25_Y.lipolytica
TLALPILPALVSTLITHTYSLRKTTYDSVLSLLSNIDA
>tip_25_S.pombe
SCIADVYNELLQGLISKSSNQRQLVFETLYSLLDIPES
>tip_25_O.tauri
ALIVPILPDILPKLLAQTAECRKSAFECVNTILDSCAG
>tip_25_A.thaliana
NLIKGLLPELLPLLYDQTVELRKAAFECVFTLVDSCLD
>tip_25_O.sativa
NLIKGLLPELLPLLYDQTVELRKAAFECVDTLLDSCLD
>tip_26_H.sapiens                                   6.1
LDIFEFLNHVEDGLKDHY-DIKMLTFLMLVRLSTLCPS
>tip_26_X.laevis
LDIFEFLNHVEDGLKDHY-DIKMLTFLMLVRLSTLCPS
>tip_26_D.rerio
LDIFEFLNHVEDGLKDHY-DIKMLTFLMLARLSSLCPS
>tip_26_A.mellifera
LDVFEFLNHVENGLRDHY-DIKMLTYLMTARLAQLCPT
>tip_26_D.melanogaster
VDVMQFLDHVQAGLCDHY-DIKMLTYLMTARLAILCPD
>tip_26_D.discoideum
IDVAPFIVSLCDGLKDTQYDIKLLCHLMIIRLANSNGA
```

```
>tip_26_C.elegans
IDITQFSSVMEVGLSDQNHDVKLLNYLTLQRVANLAPG
>tip_26_U.maydis
LHLPDYLDRVIAGLSDSDDGIKTLCYLMIIKLVELRSS
>tip_26_C.neoformans
IDLPTFTARVLVSLSDVN-EVKILGLMLLLRLGQVSPE
>tip_26_N.crassa
ISIIDLYDRIVAGLSDDN-DIKALCNLMVSKLAYIDPE
>tip_26_Y.lipolytica
QQLLALFERLLAGLSDEH-NIQILSLVCIPKAVAVDIS
>tip_26_S.pombe
NHLTHFLQVSVMGLEDEH-YIKLVSLSILEKLVDCSPS
>tip_26_O.tauri
VNARDVVSAITSGLGDHY-DIKMLAHATMLKLSEGIAS
>tip_26_A.thaliana
NPSSFIVPFLKSGLEDHY-DLKMLCHLILSLLADKCPS
>tip_26_O.sativa
NPSSFIVPFLLSGLGDHY-DVKMPCHLILSKLADKCPS
>impb_2_H.sapiens                                   85
ENLPTFLVELSRVLANPGNVARVAAGLQIKNSLTSKDP
>impb_2_O.sativa
QNLPGFLFSLSNELANEEKESRRLAGLILKNALDAKEQ
>impb_2_A.thaliana
QNLAGFLLSLAGELANDEKDSRKLAGLVLKNALDAKEQ
>impb_2_O.lucimarinus
SDAGAYAKALVDELACASAATRQLAGVILKNTLDAKDE
>impb_2_Y.lipolytica
NYYGDVMMALANCISTQDTEIRVLAGIAIKNNLTSKDQ
>impb_2_S.cerevisiae
DNFLQFAGLSSQVLIDENTEGRILAALTLKNELVSKDS
>impb_2_N.crassa
TNFSQYLVTLVQELANESAHIRAAAGIALKNAFSAREF
>impb_2_S.pombe
TDFAQYMVLLAQELANDNSYIRMAAGLALKNAITAREE
>impb_2_U.maydis
DSYPVYMSTLAAELANESSHIRTAAGLAVKNALTARDQ
>impb_2_C.elegans
QDFPVFVQCLSMILRTQQCFVRQAAGLQLKNVLCAKET
>impb_2_A.californica
QNLPELLKQLSDILKHGGNVARMQAGLQLKNTLYSKDQ
>impb_2_M.musculus
ENLPTFLVELSRVLANPGNVARVAAGLQIKNSLTSKDP
>impb_2_D.melanogaster
SNLPEFLKALSEILVNTANVARMAAGLQLKNHLTSKDE
>impb_2_T.castaneum
ANILEFIKTLSDILRHGGNVARMAAGLQLKNQLTSKDP
>impb_2_A.mellifera
TNLHEFLQRLSSVLVTAAAVARMAAGLQLKNQLTSKDP
>impb_3_H.sapiens                                   2.2
NARREVKNYVLHTLGTETY-RPSSASQCVAGIACAEIP
>impb_3_O.sativa
GVKAQIKGFLLQTLSSPVASARSTSSQVIAKVAGIEIP
>impb_3_A.thaliana
STKSQIRAFLLKTLSAPVPDVRSTASQVIAKVAGIELP
>impb_3_O.lucimarinus
ATREEIKRAAWGCLACGEAPVRSVAAQVVAKIAGAEVP
>impb_3_Y.lipolytica
SVTDQIKSILLEVLKSTNNQVASAAAQAVAAIAEIDLP
>impb_3_S.cerevisiae
EAKNQIKTNALTALVSIEPRIANAAAQLIAAIADIELP
>impb_3_N.crassa
DTKTRVKQLTLETLASSSTQASQASAQVIAAIATIELP
```

```
>impb_3_S.pombe
EIKQQVKSLALQTLGSSEHQAGQSAAQLVAAIAAYELA
>impb_3_U.maydis
GSRDDIKQKVLSTLGSQEHRAGTAAAQVVAAIAAIELP
>impb_3_C.elegans
EVREQVKQNVTGTLGTEPS-RPSIAAQCVAAIACAELP
>impb_3_A.californica
DVRNHVKQNVLATLGTETV-RPSSAAQCVAYIACAELP
>impb_3_M.musculus
NARREVKNYVLQTLGTETY-RPSSASQCVAGIACAEIP
>impb_3_D.melanogaster
EIRELIKNNILAALGTENT-RPSCAAQCVAYVAVIELP
>impb_3_T.castaneum
EIRNYVKKNVVGALGTETN-RPSSAAQCVAYIAVTELP
>impb_3_A.mellifera
ETREYIKKNIAGALGTENN-RPGSAPQCVAYVAVAELP
>impb_4_H.sapiens                              0.19
NQWPELIPQLVANVTNPNSHMKESTLEAIGYICQDIDP
>impb_4_O.sativa
KQWPELIASLLSNIHQVQPNVKQATLETLGYLCEEVSP
>impb_4_A.thaliana
KQWPELIVSLLSNIHQLPAHVKQATLETLGYLCEEVSP
>impb_4_O.lucimarinus
KAWPDLIPSLQRGAQGGGDGAKQASLEALGYVCEEVDA
>impb_4_Y.lipolytica
GRWSSLMTTLVENTKDEQPHIKMAALQSIGFICERADR
>impb_4_S.cerevisiae
GAWPELMKIMVDNTGAEQPNVKRASLLALGYMCESADP
>impb_4_N.crassa
NEWPDLMHALVKNVSEGSEHQKQASLTTIGFICESQDV
>impb_4_S.pombe
NQWPDLMVTLVANVGEGQPALKQHSLQTIGYICESVSP
>impb_4_U.maydis
GLWNELISQLLSAMGDANNRLRQAALQAIGFTCEGISS
>impb_4_C.elegans
NLWPNVINLLKSNVTESQSMLKESSLETLGYICQDIDP
>impb_4_A.californica
KLWPDLIVNCTRNVINPASMMKESTLDAVGYICQDIDP
>impb_4_M.musculus
SQWPELIPQLVANVTNPNSHMKESTLEAIGYICQDIDP
>impb_4_D.melanogaster
NRWPMLIQTLVNKVVSEGSMHRESALEAIGYICQDIRF
>impb_4_T.castaneum
HQWPDLIVTLVNNVVQPNSMQKEATLETIGYICQEIDS
>impb_4_A.mellifera
REWTNVIQLLVNNVVNPNSMLKEATLEAIGYICQDIES
>impb_5_H.sapiens                              0.22
DKSNEILTAIIQGMRKEEPNVKLAATNALLNSLEFTKA
>impb_5_O.sativa
DQVNKILTAVVQGMNASEGDVRLAATRALYNALGFAQV
>impb_5_A.thaliana
EHVNKILTAVVQGMNAAEGDVRLAATRALYMALGFAQA
>impb_5_O.lucimarinus
ADVNGVLTAVVSAMGRGETGVRLAATQALNNALYFAHE
>impb_5_Y.lipolytica
SQASGILTAIVQAAQSKESNVRLKAIEALGDSLDFIRD
>impb_5_S.cerevisiae
SSSNNILIAIVQGAQSTETAVRLAALNALADSLIFIKN
>impb_5_N.crassa
QHSNAILTAVVQGARKEEPEVRLAAITALGDSLEFVGN
>impb_5_S.pombe
AQSNAILTAVVAGARKEEPAVRLAALGALYDSLEFVRE
```

```
>impb_5_U.maydis
AQSNEILTAVIQGARKEEPEVQLAALQALFNSLEFVRA
>impb_5_C.elegans
TKANDVLTAIIHGMRPEESNVRFAATNALLNSLEFTNT
>impb_5_A.californica
PQSNDILTAIVHAMKKEEPHVRLAATNALLNSLEFTKD
>impb_5_M.musculus
DKSNEILTAIIQGMRKEEPNVKLAATNALLNSLEFTKA
>impb_5_D.melanogaster
NQSNDVLTAIIHGMRKVEPHVRLAATTALHNSLEFTKS
>impb_5_T.castaneum
TQSNDILTAIIHGMRSTEPHVRFAATQALLNSLEFTKA
>impb_5_A.mellifera
PQSNEILTAIIHGMKGSSTYVRLAATSALYNSLEFTKG
>impb_6_H.sapiens                              0.071
SERHFIMQVVCEATQCPDTRVRVAALQNLVKIMSLYYQ
>impb_6_O.sativa
MERDYIMRVVCEATQSTDVKIRQAAFECLVAISSTYYD
>impb_6_A.thaliana
MERDYIMRVVCEATLSPEVKIRQAAFECLVSIASTYYE
>impb_6_O.lucimarinus
QERDFIMQCVCEATTCEDARVRVAAFEVLVGIAENYYE
>impb_6_Y.lipolytica
GERNCIMVVVCEATQSDSAKLREVSYGTMSRIMTKYYQ
>impb_6_S.cerevisiae
GERNYLMQVVCEATQAEDIEVQAAAFGCLCKIMSKYYT
>impb_6_N.crassa
GERNYIMQVICEATQAEDSRIQQGAYGCLNRIMALYYE
>impb_6_S.pombe
YERNYIMQVVCEATQSPEASIQTAAFGCLVKIMHLYYD
>impb_6_U.maydis
GERNYIMQVVCEATQSPNMPVKVAAYECLVRIMQLYYD
>impb_6_C.elegans
AERNIIMQVVCESTSSSDQRVKVAALQCLVRIMQLYYE
>impb_6_A.californica
TERHLIMQVVCEATQSTDVRVRVAAMQCLVKIMSLFYI
>impb_6_M.musculus
SERHFIMQVVCEATQCPDTRVRVAALQNLVKIMSLYYQ
>impb_6_D.melanogaster
MERNFIMEVVCEATQCQDSQICVAALQCLVKIMTLYYQ
>impb_6_T.castaneum
TERNFIMEVVCEATQSPDTQIKVAALQCLVKIMSLYYQ
>impb_6_A.mellifera
TERNFIMEVVCEATQSLNTQVKVAALQCLVKIMSLYYQ
>impb_7_H.sapiens                              510
TYMGPALFAICIEAMKSDIEVALQGIEFWSNVCDEEMD
>impb_7_O.sativa
TYM-QDIFNITAKAVRGDESVALQAIEFWSSICDEEID
>impb_7_A.thaliana
HYM-QDIFNITAKAVREDDSVALQAIEFWSSICDEEID
>impb_7_O.lucimarinus
AYI-EAVYELTVKAAKQDQEVGLQAIEFWSTICEEEIG
>impb_7_Y.lipolytica
LYMKQALFGVTVKGMQDSDSVACMAVEFWSSVCE----
>impb_7_S.cerevisiae
PYMEQALYALTIATMKSPNKVASMTVEFWSTICEEEID
>impb_7_N.crassa
FYMEKALFGLTILGMKSDDDVAKLAVEFWSTVCEEEIA
>impb_7_S.pombe
FYMEKALFALTTQGMYNTNQVALQAVEFWSTVCEEEIE
>impb_7_U.maydis
FYMEQALFGLTVLGMRDSEKVALQAVEFWSTVCDEEIE
```

>impb_7_C.elegans
SYMGSALFQITLSAMKSQEEVAMQGMEFWSTVAEEEFD
>impb_7_A.californica
HYMGPALFAITIDAMEHENEIALQGIEFWSTVCDEEVD
>impb_7_M.musculus
TYMGPALFAITIEAMKSDIEVALQGIEFWSNVCDEEMD
>impb_7_D.melanogaster
PYMAQALFPITLAAMKSDNAVALQGIEFWSNVCDEEID
>impb_7_T.castaneum
PYMGQALFPITLEAMKSDNAVALQGIEFWSNVSDEEVD
>impb_7_A.mellifera
PYMAPALFPITLEAMKSDIEVALQGIEFWSNVSDEEVD
>impb_8_H.sapiens 0.32
GALQYLVPILTQTLTKQDENPCKAAGVCLMLLATCCED
>impb_8_O.sativa
QALPALVPMLLETLLKQEENLAMAGGTCLGLVARTVGD
>impb_8_A.thaliana
QALPGLVPLLLETLLKQEENIAMAGGTCLGLVARAVGD
>impb_8_O.lucimarinus
TALGALVPMLLEQLTKQEDNLAMAGGICLGLVAQLVRD
>impb_8_Y.lipolytica
VAAPKVLPILLELLNRQNESVSMAAAACLQLFAQTIGN
>impb_8_S.cerevisiae
SSIKDVVPNLLNLLTRQNENVSMSAGACLQLFAQNCGN
>impb_8_N.crassa
VATLEVVPVLLQLLTKQDENISRAAYQCLQLYSQAVGA
>impb_8_S.pombe
AAAADILPVLLKLLCNQDENISMAAATCLQLFAQVVGD
>impb_8_U.maydis
IALPDIVPVLLELLKTQDEDVSKAAGTCVGLLAQVVGD
>impb_8_C.elegans
QAASHVCPVLLEAMAHHDDTPAKAAGVCLMLAAQCVRD
>impb_8_A.californica
GALQYLVPILLVSLTKQEENPCKAAGVCLMLLATCCED
>impb_8_M.musculus
GALQYLVPILTQTLTKQDENPCKAAGVCLMLLSTCCED
>impb_8_D.melanogaster
GALQFLTPVLVEKLTKQDESPAKAASVCLMVLATCCED
>impb_8_T.castaneum
GALQFIVPILLQKLTKQEENPSKAAGVCLMLLATCCEN
>impb_8_A.mellifera
GALQYLVPVLMKKLTKQEENPSKAAGVCLMLLSSCCED
>impb_9_H.sapiens 9.1E-04
DIVPHVLPFIKEHIKNPDWRYRDAAVMAFGCILEGPEP
>impb_9_O.sativa
DIVPLVMPFVEENITKPDWRHREAATYAFGSILEGPSA
>impb_9_A.thaliana
DIVPHVMPFIEEKISKPDWREREAATYAFGSILEGPSA
>impb_9_O.lucimarinus
PVVEQVMAYIQANIRSSEWRQREAATFAFGAILEGPNP
>impb_9_Y.lipolytica
DVVPLTLQFVEQNIGNTTSRNREAAVMAFGSILDGPDN
>impb_9_S.cerevisiae
HILEPVLEFVEQNITADNWRNREAAVMAFGSIMDGPDK
>impb_9_N.crassa
AIIQPVIQFVEANLRADDWHLRDAAVSAFGAMMDGPEE
>impb_9_S.pombe
LIVNPVLAFVEQNIQNPDWHQREAAVMAFGSVLEGPNV
>impb_9_U.maydis
DIVRLAVPFVEGNIKNPDWHAREAAVMCFGSIMEGPDR
>impb_9_C.elegans
DIVNHVIPFFK-HFQNPDWKYKEAAIMAFGSILDGPDP

>impb_9_A.californica
DVVQHVLPFVRDNIRHEDWRYRDAAVMAFGSVLEGPDP
>impb_9_M.musculus
DIVPHVLPFIKEHIKNPDWRYRDAAVMAFGSILEGPEP
>impb_9_D.melanogaster
EIVPHVLPFIKENIESPNWRFRDAAVMTFGSVLNGLET
>impb_9_T.castaneum
EVVPHVLPFIKENIKSENWRFRDASLMAFGSILGGLDN
>impb_9_A.mellifera
AIVPFVLPFIKDSIKSPDWRYRDAALMAFGSILGGVDH
>impb_10_H.sapiens 1.2E-03
PLVIQAMPTLIELMKDPSVVVRDTAAWTVGRICELLPE
>impb_10_O.sativa
PLVNVALNFMISALVNDPSHVKDTTAWTLGRIFEFLHG
>impb_10_A.thaliana
AIVNAALTFMINALTNDPSHVKDTTAWTLGRIFEFLHG
>impb_10_O.lucimarinus
GIAKEALPVLVMALKDDSTHVKDTTAWTIGRVFEFVHT
>impb_10_Y.lipolytica
DLIKQALEPILNLMNDDSLQVKDTVAWCLGRISDLVIN
>impb_10_S.cerevisiae
YYVHQALPSILNLMNDQSLQVKETTAWCIGRIADSVAE
>impb_10_N.crassa
PIVKSGMQPLIGMMEDPSLHVRDSTAYALGRITETCSE
>impb_10_S.pombe
PLVNQALPVLINMMVDPVIFVKDTTAWALGQISSFVAD
>impb_10_U.maydis
PLVESALPIIIEMLRDQSIAVKDTAAWTLGRISDLCCD
>impb_10_C.elegans
PMAQEALPAIVAAMCDKNVNVRDTAAWSLGRVIDTCSE
>impb_10_A.californica
PIVEQAMPMLIELLKDPSVVVRDTAAWTVGRVCEILPN
>impb_10_M.musculus
PLVIQAMPTLIELMKDPSVVVRDTTAWTVGRICELLPE
>impb_10_D.melanogaster
PLVEQAMPTLIRLMYDSSVIVRDTIAWTFGRICDIIPE
>impb_10_T.castaneum
PLVEQAMPTLIELMYDSSVIVRDTAAWTFGRICEIIPE
>impb_10_A.mellifera
PLVEQAMPTLIELMYDSSVAVRDTAAWTFGRICEIIPE
>impb_11_H.sapiens 0.17
VYLAPLLQCLIEGLSAEP-RVASNVCWAFSSLAEAAYE
>impb_11_O.sativa
ENCQQILTVLIQSMKDVP-NVAEKACGALYFLAQGYVD
>impb_11_A.thaliana
ANCQQIITVLIQSMNDAP-NVAEKACGALYFLAQGYED
>impb_11_O.lucimarinus
QTFPQVLQAMMESLKDVP-HVAGKVCWSVQNLVSAISQ
>impb_11_Y.lipolytica
VHLPVIMNTLLKGLQDEP-KVITNCCWTIMNIFEQLGH
>impb_11_S.cerevisiae
QHLPGVVQACLIGLQDHP-KVATNCSWTIINLVEQLAE
>impb_11_N.crassa
VHLDPLITSLFNGLMSSP-RMAASCCWALMNLAERFGG
>impb_11_S.pombe
IHLSPMVSALLQGLTDNP-RIVANCCWAFMNLVCHFAP
>impb_11_U.maydis
VHLPALVQALVLGLQDEP-RIVTNCCWAIMNLSEQLGT
>impb_11_C.elegans
ELLQSVLPVLSNGLHQEP-RVANNVCWALVSLVKACYE
>impb_11_A.californica
ACLHPLLNALVEGLVAEP-RVASNVCWAFSSLAEAAYD

>impb_11_M.musculus
VYLAPLLQCLIEGLSAEP-RVASNVCWAFSSLAEAAYE
>impb_11_D.melanogaster
TYLQTLLECFVKSLKSEP-RVAANVCWAFIGLSDAAWE
>impb_11_T.castaneum
NYLKPLLESLINGLKAEP-RVAANVCWAFSGLAEAAYD
>impb_11_A.mellifera
TYLKPLLEALINGLKAEP-RVAANVCWAFTGLAEASYE
>impb_12_H.sapiens 0.28
SSFELIVQKLIETTDRPDGNLRSSAYESLMEIVKNSAK
>impb_12_O.sativa
PFFQDIIQSLIFVTHREDARLRTAAYETLNEVVRCSIE
>impb_12_A.thaliana
PFFQEIIKSLLAVAHREDARLRTAAYEALNEVVRCSTD
>impb_12_O.lucimarinus
PYFQSIIQTLLITSERPDAGLKMECYESMNEILRSSTE
>impb_12_Y.lipolytica
PYYPQVLPALLNAASRNDNNARTAAYEALSTLVVVCAN
>impb_12_S.cerevisiae
NFYPALVDGLIGAANRIDNNARASAFSALTTMVEYATD
>impb_12_N.crassa
PHFNQCVTNLIAVTAKLDGTVRTAAYEVLNVFVQNAAN
>impb_12_S.pombe
PFYEAIIGSLLHVTDQKGNNSRTSGYETLGTLITFSSD
>impb_12_U.maydis
PFFEGIVSSLLQATGRSSNNSRTSAYEALASSITHCAA
>impb_12_C.elegans
SVFDPMVGELLKITDRVDGNLRITAYEALMELIKHSPK
>impb_12_A.californica
NYFETIVERLIQTAERTDGNLRNAAYEALMELVKNSPK
>impb_12_M.musculus
SSFELIVQKLLETTDRPDGNLRSSAYESLMEIVKNSAK
>impb_12_D.melanogaster
PYFEYIITQLLETTDRSDGNLRCAAYQALMDMIKNSPL
>impb_12_T.castaneum
QYFEYIIQRLLETTDRPDGNLRPAAYEALMEMVKNSPK
>impb_12_A.mellifera
QYFDFIIQRLLETTDRPDGNLRSAAYEALMDMVKNSPR
>impb_13_H.sapiens 130
KTTLVIMERLQQVLQMESHDLQSLLCATLQNVLRKVQH
>impb_13_O.sativa
QLVPVIMMELHQTLEAGKLELQGLLCGCLQVIIQKLGA
>impb_13_A.thaliana
QLVPVIMMELHNTLEGEKLELQGLLCGCLQVIIQKLGS
>impb_13_O.lucimarinus
QLIPHVLQKLSATLVGQEQDAQALLCGTLQVIIQVLGA
>impb_13_Y.lipolytica
ELSGEVVTRLETTLEMQQQELQINLLGLLTNIIRRTD-
>impb_13_S.cerevisiae
SISTFVMDKLGQTMSVDENELQSNILTVLAAVIRKSPS
>impb_13_N.crassa
SLSDVILQRLEETLPLQSQDMQTSLCTVLQAIIQRLD-
>impb_13_S.pombe
NVLSIILTRLETSIQMQSQELQSNLCNVLTSIIRRFGP
>impb_13_U.maydis
GVLVQILDRQQQLNEVAGQELQGNLCSVLMACVRRLGR
>impb_13_C.elegans
NTTVVILKKLESLLQMESQDLQAMLCATLQSVTRKMQP
>impb_13_A.californica
KTTMTILERLERVLSMESADLQSLLCATLQSVLRKVTP
>impb_13_M.musculus
KTTLVIMERLQQVLQMESHDLQSLLCATLQNVLRKVQH

```
>impb_13_D.melanogaster
RTTLVILERLNQVMQMETQDLQSLLCATLQSVLRKVHE
>impb_13_T.castaneum
KTTMVILERLQQVLQMETHDLQSLLCGTLQSVLRKVTP
>impb_13_A.mellifera
KTTMVILERLQQVLQMETHDLQSLLCATLQSVLRKVTP
>impb_14_H.sapiens                                0.7
QISDVVMASLLRMFQSTAGGVQEDALMAVSTLVEVLGG
>impb_14_O.sativa
QYADQMMELFLRVFACRN-TVHEEAMLAIGALAYAAGP
>impb_14_A.thaliana
EYADQMMGLFLRVFGCRS-TAHEEAMLAIGALAYAAGP
>impb_14_O.lucimarinus
AHADSLMHAFLSVFSCRS-TVHEEAMLAVGALAYAVGE
>impb_14_Y.lipolytica
PASDRLMTLFLNLLQNKLPLIEEDVFIAIGAVADANGE
>impb_14_S.cerevisiae
PVADMLMGLFFRLLEKKD-FIEDDVFYAISALAASLGK
>impb_14_N.crassa
PQGDRIMQVLLQLLNTINGAVPEGVFAAISGLANAMEE
>impb_14_S.pombe
S-SDQIMNLLLQTMQTAPKVVHEDVLLAIGAMMNSLEE
>impb_14_U.maydis
L-GDRIMTNLLTLIQNGSKTVLEDAFFTVGAVIAAFEA
>impb_14_C.elegans
AVGEHIMNGLYQIMNRAAAAVMEEALLAVACLAEHLGK
>impb_14_A.californica
KISDQIMTALLRMFQSTTGGVQEDALLAMSTLIEVLGD
>impb_14_M.musculus
QISDVVMASLLRMFQSTAGGVQEDALMAVSTLVEVLGG
>impb_14_D.melanogaster
QISDAIMTALLTMFNSSAGVVQEEAFLAVSTLVELLGA
>impb_14_T.castaneum
QISDAIMTAMLTMFNSNSCGVQEDALMAVSTLVEVLGE
>impb_14_A.mellifera
HISDVIMTALLSMFNSNSCGVQEDALMAVSTLVEVLGE
>impb_15_H.sapiens                                0.5
KYMEAFKPFLGIGLKNYAEQVCLAAVGLVGDLCRALQS
>impb_15_O.sativa
KYMPQFYQYLEMGLQNFEEQVCAITVGVVGDLCRALED
>impb_15_A.thaliana
KYMPEFYKYLEMGLQNFEEQVCAVTVGVVGDVCRALED
>impb_15_O.lucimarinus
KYMDAFIPYVKLGLENHEEQVCAVTVGVVGDICRALDD
>impb_15_Y.lipolytica
KFMESLNPFLLRALEDPSL-VCNTAVGLVADVSNALGP
>impb_15_S.cerevisiae
KYLETFSPYLLKALNQVDS-VSITAVGFIADISNSLEE
>impb_15_N.crassa
KYMDAFAPFLYNALANQEESLCSMAIGLVSDITRSMGE
>impb_15_S.pombe
VYVPSFVPFLSSALSNEQEQLCSVAVGLVGDLARALNA
>impb_15_U.maydis
KYLGAFLPFMVEGLRNHEEQLCSISVGLIGDICRALGE
>impb_15_C.elegans
SYMNVLKPYLLEGLSNTDEQVCAAAVGLVTDLSRALEA
>impb_15_A.californica
KYMDAFKPYLLLGLKNFAEQVCLAAVGIVGDLCRALGQ
>impb_15_M.musculus
KYMEAFKPFLGIGLKNYAEQVCLAAVGLVGDLCRALQS
>impb_15_D.melanogaster
KYMPAFKDFLVMGLKNFQEQVCCAAVGLTGDIFRALKD
```

>impb_15_T.castaneum
KYMDAFKPFLYIGLKNHQEQVCGTAVGLTGDIFRALKL
>impb_15_A.mellifera
KYMDAFKPYLCLGLKNYAEQVCCAAVGLTGDICRALKS
>impb_16_H.sapiens 0.41
PFCDEVMQLLLENLGNENVSVKPQILSVFGDIALAIGG
>impb_16_O.sativa
PYCDGIMTQLLKDLSSNQLSVKPPIFSCFGDIALAIGE
>impb_16_A.thaliana
PYCDGIMTQLLKDLSSNQLSVKPPIFSCFGDIALAIGE
>impb_16_O.lucimarinus
PYCEPIVYLLLRDLGSEKLSVKPPILSCFGDLALATGA
>impb_16_Y.lipolytica
QFSDQYMQLFVTDLQNPKAVIKPSILSCFGDIASSIGP
>impb_16_S.cerevisiae
RYSDAMMNVLAQMISNPNAELKPAVLSVFGDIASNIGA
>impb_16_N.crassa
PYCDQFMNYLLNNLRSTALQFKPAILQCFGDIAGAIGG
>impb_16_S.pombe
PYCDDFMTRLVQDLQSSVLNVKPAILSCFSDIALAIGA
>impb_16_U.maydis
KYCDDFMNALFANLQSPQLSVKPPILSCFGDIAMAIGA
>impb_16_C.elegans
PFMDELIQKLILCLQSPRLNVKVVIIGTFADIAMAIEA
>impb_16_A.californica
PHCDELMMMLLENLGNDSVSVKPQILSVFGDIALAIGM
>impb_16_M.musculus
PFCDEVMQLLLENLGNENVSVKPQILSVFGDIALAIGG
>impb_16_D.melanogaster
PYSNEIMTVLINNLTEPTITVKPQVLSAFGDIALSIGN
>impb_16_T.castaneum
PYCDEIMTLLLENLGDQSVSVKPQILSVFGDIVLSIGP
>impb_16_A.mellifera
PYCDEIMTLLLENLSNDSVSVKPQIFSVFGDVALSIGP
>impb_17_H.sapiens 3.4
KYLEVVLNTLQQASQAQVDELRESCLEAYTGIVQGLKG
>impb_17_O.sativa
KYLIYAMPMLQSAADLSAHQLRNGILEAYSGILQGFKS
>impb_17_A.thaliana
KYWRYSMPMLQSAAELSAHSLRNGILEAYSGIFQGFKN
>impb_17_O.lucimarinus
KFLSYVVGMLQSAMQLSANELRNGIFEAYAGILQGLKL
>impb_17_Y.lipolytica
RYFSFVFPVIEQACQLDVPNLRERIIDAFVGIVTGLRD
>impb_17_S.cerevisiae
PYLNDIMALCVAAQNTKPEKVLEAVLDAYVGIVAGLHD
>impb_17_N.crassa
AYLSVVAVVLQQAATVTASSLREGIMDAWGGIIGAMKG
>impb_17_S.pombe
TYLEAVMVLLQQASSVQAPALRLGIVEAYVGITQAVRT
>impb_17_U.maydis
KYLQMGMSVLQQASLIQT-SLREGICEAYVGTVSGMRA
>impb_17_C.elegans
RYVGSVVPILNNAQNAAVVRLREACLNSYTGIIQGLKA
>impb_17_A.californica
HYLDVVMTTLQQASQAQV-ELRRGCLEAYTGIVQGFEG
>impb_17_M.musculus
KYLEVVLNTLQQASQAQV-ELRESCLEAYTGIVQGLKG
>impb_17_D.melanogaster
PYLSMVLDMLRVASNLQT-ELRESILEAYTGIIQGLKG
>impb_17_T.castaneum
KYLDVVLTTLAQASQAQV-ELREGVLDAYTGIVQGLKG

```
>impb_17_A.mellifera
KYLDVVLQTLAQASQANV-ELREGVLEAYTGIVQGLRG
>impb_18_H.sapiens                              1.1
PRVEFILSFIDHIAGDEDHGVVACAAGLIGDLCTAFGK
>impb_18_O.sativa
QYAPNILNFLDALYNGKDMTVMKTAIGVLGDLADTLGV
>impb_18_A.thaliana
PFAPHILQFLDSIYMEKDMVVMKTAIGVLGDLADTLGS
>impb_18_O.lucimarinus
EHVPFVVDFIEAVSKDPNCTVTRSMVGVLGDIANCFRG
>impb_18_Y.lipolytica
PYVPTLFNFLSKVAVDPLLSVARSVVGIIGDVATMYPG
>impb_18_S.cerevisiae
PYVGTIFQFIAQVAEDPQLATSRAAVGLIGDIAAMFPD
>impb_18_N.crassa
PYVQSIFELLNTIAQDPNRALMRAAMGVIGDLADAYPN
>impb_18_S.pombe
PYVHSMFTLLNMITADPECSLTRAALGLLGDLAESFPK
>impb_18_U.maydis
PYVEGMYAFIGLVAHAQTQPLIRGALGLLGDIASAYPN
>impb_18_C.elegans
VFVEPIVQLITRISSMEPVALIATTAGIIGDLVQLYEG
>impb_18_A.californica
PHVNHIVSFIEHISVDDDKSNVAACCGLIGDLCSAFSH
>impb_18_M.musculus
PRVEFILSFIDHIAGDEDHGVVACAAGLIGDLCTAFGK
>impb_18_D.melanogaster
PHLMHIISFIKRIAQEGDVSMLASAAGFIGDLCTSFGP
>impb_18_T.castaneum
PHIPFIVQFITVVAQDTFNATVAVAAGLVGDLCTAFGA
>impb_18_A.mellifera
PHVPFIIQFITSIAQDREHGNISASVGLLGDLVTVFGV
>MSPS_2_H.sapiens                               1.5
GDYADLVKALKKVVGKDTNMLVALAAKCLTGLAVGLRK
>MSPS_2_G.gallus
GDYADLVKVLKKVVGKDTNMLVALAAKCLAGLASGLRK
>MSPS_2_X.laevis
GDYADLVKALKKVVGKDNNMLVALAAKCIAGLAAGLRK
>MSPS_2_A.mellifera
GDYGDLVRALKKIISKDTNLVVALAGKCLAGLAAGLKK
>MSPS_2_D.melanogaster
GEYGALVSALKKVITKDSNVLVAMAGKCLALLAKGLAK
>MSPS_2_O.sativa
GDFHEICRTLKKLITDVNLAVSVEATQAIGNLAKGLRT
>MSPS_2_P.patens
GDFSEVSRILKKLVSDVNIAVAVEAVQAIGNLASGLRK
>MSPS_2_A.thaliana
GDFSEICRTLKKLITDVNLAVAVEAIQAIGNLACGLRT
>MSPS_2_D.discoideum
ADFSELCKALKKILADVNVMIVQKAVVSIGLLADSLRG
>MSPS_2_C.elegans
ANYGALVERLQKVLEKDANNVAALAANCITGIANGLRT
>MSPS_2_C.cinerea
PELSELAKALATCISKDANNCVMVAANCLEGLAKAMMD
>MSPS_2_S.cerevisiae
QNYSNLLGIYGHIIQKDANQAVALAAQSVELICDKLKT
>MSPS_2_Y.lypolytica
GDYGELLRAMAKVVHKDANQCVQVAAQCIETVANGLPG
>MSPS_2_S.pombe
NDFFNLVAILTKSVSKDANMVVINAAHCIQAMAKGLRS
>MSPS_2_M.grisea
GDFNEINRAFAKSMKDANIAVVIQAAQCLEALAKGLRK
```

```
>MSPS_3_H.sapiens                                 6.2
QYAGHVVPTILEKFKEKKP-QVVQALQEAIDAIFLTTT
>MSPS_3_G.gallus
QYAGHVVPTILEKFKEKKP-QVVQALQEAIDAIFLTTT
>MSPS_3_X.laevis
SYAGHVVPTILEKFKEKKP-QVVQALQEAIDAVFLTTT
>MSPS_3_A.mellifera
PYAIACLSSILEKFREKKQ-NVVQALREAADAIFLSVS
>MSPS_3_D.melanogaster
NYASACVPSLLEKFKEKKP-NVVTALREAIDAIYASTS
>MSPS_3_O.sativa
GNSRVLLPVLLEKLKEKKP-TMTEALSQTLQAMHKSGC
>MSPS_3_P.patens
SGSKLLLPVLLDKLKEKKQ-VMVDALTTTLNAMHKGGC
>MSPS_3_A.thaliana
ASSRFMLPVLLEKLKEKKQ-SVTDPLTQTLQTMYKAGC
>MSPS_3_D.discoideum
SYVKPFITPILEKFKEKKT-SVLQSVHTTMDSLVGKSI
>MSPS_3_C.elegans
PFAVSVTPIIFEKFKEKKP-TLRDPLVACIDAVVATTN
>MSPS_3_C.cinerea
KYRESTVPLMLVRLKERKA-TVTDAIGAALDAIFSTVT
>MSPS_3_S.cerevisiae
DYVSLVFTPLLDRTKEKKP-SVIEAIRKALLTICKYYD
>MSPS_3_Y.lypolytica
ARYRFTLQAVLERTKEKKA-SVAEALGKALDAIACKLP
>MSPS_3_S.pombe
KYASTSINALLERSKEKKA-NVIESLSSAMDAVLATSS
>MSPS_3_M.grisea
KYRSVVMQPILERLKEKKA-TVTDALGAALDAVFMSTS
>MSPS_4_H.sapiens                                 1.3
TTLQNISEDVLAVMDNKNPTIKQQTSLFIARSFRHCTA
>MSPS_4_G.gallus
TTLQNISEDVLAVMDNKNPTIKQQTSLFIARSFRHCTP
>MSPS_4_X.laevis
TTLQNVSEDILAVMDNKNPAIKQQASLFLARSFRQCTA
>MSPS_4_A.mellifera
VSIDVILEDTLAALENKNPAVKAETAIYLARCFSRTPP
>MSPS_4_D.melanogaster
TSLEAQQESIVESLSNKNPSVKSETALFIARALTRTQP
>MSPS_4_O.sativa
ITLLDVIEDVRVAVKNKVPLVRSLTLNWVAFCIETSNK
>MSPS_4_P.patens
IQLQDVIEDVKLATKNKVPLVRSSCLTWVATCIETSNK
>MSPS_4_A.thaliana
LNLVDVIEDVKTAVKNKVPLVRSSTLTWLTFCLETSNK
>MSPS_4_D.discoideum
ISLSDIIDELTATMQSKVPQIKQEVLVFICNSITNTKK
>MSPS_4_C.elegans
TNLEAVGEIVLAALGKPNPSIKTQTDLFLQRCFMKLNS
>MSPS_4_C.cinerea
VTLNDILPDLEPALKDKNPQVKEGTLKFLGRALSAATS
>MSPS_4_S.cerevisiae
GRNEDMLKDILEHMKHKTPQIRMECTQLFNASMKEEKD
>MSPS_4_Y.lypolytica
LPFGEVVEDTLPFVSHKTPQVRIESLNFILRLLSTIKE
>MSPS_4_S.pombe
SSLDDLAELIASFAGNKNPQIKSSCFSLFSRSFSNMTS
>MSPS_4_M.grisea
TSLSECLEDITAYMIHKNPQVKEGTAKFLIRCLRTTRD
>MSPS_5_H.sapiens                                 2.9E-04
SLLKPFCAALLKHINDSAPEVRDAAFEALGTALKVVGE
```

>MSPS_5_G.gallus
SLLKPFCAALLKHINDSAPEVRDAGFEALGTALKVAGE
>MSPS_5_X.laevis
SLLKPFCAALLKQINDSAPEVRDAAFEALGTALKVVGE
>MSPS_5_A.mellifera
KLLKAYTAVLLKTLNEPDPTVRDSSAEALGTAMKLIGE
>MSPS_5_D.melanogaster
KLLKLLTTSLVKTLNEPDPTVRDSSAEALGTLIKLMGD
>MSPS_5_O.sativa
KLHKEYVPICMECLNDGTPEVRDASFSVLTAIAKMVGM
>MSPS_5_P.patens
KLHKEYIPILMECLNDSSPEVRDAAFLALAAFAKVVGM
>MSPS_5_A.thaliana
KAHKEYVPLCMECLNDGTPDVRDAAFSALAAIAKSVGM
>MSPS_5_D.discoideum
KVTKQLTKIFMEALNDTDSNIRDNASKAFAALGGIIGE
>MSPS_5_C.elegans
KTLKTLIPSLIKHSGDSDSEVREASYAAMGAMMRAIGE
>MSPS_5_C.cinerea
NQIKPLSESLAVLLEDGFEGARNEAANCFGTLMKMVGE
>MSPS_5_S.cerevisiae
YLKDEVVPIVIQIVNDTQPAIRTIGFESFAILIKIFGM
>MSPS_5_Y.lypolytica
QDLDHVIKAVLKTLSDTQAPVREISMQVLGTARKLIGE
>MSPS_5_S.pombe
FTVDTCAKACVPGVSDTFEPVRSAAAEALGVLMKLVGE
>MSPS_5_M.grisea
PEINTMVESAKKLLAESTETLRASGAEILGTIMKIIGE
>zyg_2_C.elegans no match
-KVSLMSQLFHKDFK-----QHLAALDSLVRLADTSPR
>zyg_2_O.sativa
-REDVSLRLWNSDFK-----RQIDGIELLQKALPSSRK
>zyg_2_N.tabacum
-REDLHRRLLSTDFK-----KQVDGIEMLQKALPSIAK
>zyg_2_A.thaliana
-REDLQKRLLSPDFK-----KQVDGLEILQKALPSVSK
>zyg_2_O.tauris
-RADVHKLMFTNDMK-----AHLAALDALEEAIKSDEA
>zyg_2_X.laevis
-AKWLQDELFHADFQ-----HHIKGLAVMTERLESEKE
>zyg_2_G.gallus
-AKWLQDEMFHADFQ-----HHNKALAVMIEHLENEKD
>zyg_2_M.musculus
-AKWLQDEMFHSDFQ-----HHNKALAVMVDHLESEKD
>zyg_2_H.sapiens
-AKWLQDEMFHSDFQ-----HHNKALAVMVDHLESEKE
>zyg_2_C.familiaris
-AKWLQDEMFHSDFQ-----HHNKALAVMVDHLESEKE
>zyg_2_T.castaneum
-NKTLISNMFHNDFG-----YHIKALDSLMEDLNDNSA
>zyg_2_A.mellifera
-NKTLLANMFHSDFR-----YHLKAIEALTEDLPDNSK
>zyg_2_D.melanogaster
-NKALIANMFHDDFR-----YHLKVIEQLSEDLAGNSK
>zyg_2_A.aegypti
-NKGLIANMFHEDFR-----YHLKVIDALVEDLPKNDK
>zyg_2_A.gambiae
-NKALMVNMFHDDFR-----YHLKVIDALMEDLATNEE
>zyg_3_C.elegans 43
SNSDLLLKWCTLRFFETNPAALIKVLELCKVIVELIRD
>zyg_3_O.sativa
ELLDILLRWFVLRFCESNTTCLLKVLDFLPELFDVLKD

>zyg_3_N.tabacum
EVLDIVLRWFVLRFCESNTSCLLKVLEFLPELFEMLRN
>zyg_3_A.thaliana
EVLDVLLRWFVLQFCKSNTTCLLKVLEFLPELFNTLRD
>zyg_3_O.tauris
NSLDLLIRWLVLRISESSPQVLTRVLEVVLAALHAVCD
>zyg_3_X.laevis
SCLDLILKWFTLRFFDTNTSVLMKCLEYLKLLFIMLSQ
>zyg_3_G.gallus
SCLDLILKWLTLRFFDTNTSVLMKTLEYLKLLFNMLSQ
>zyg_3_M.musculus
SCLDLILKWLTLRFFDTNTSVLMKALEYLKLLFTLLSE
>zyg_3_H.sapiens
GCLDLILKWLTLRFFDTNTSVLMKALEYLKLLFTLLSE
>zyg_3_C.familiaris
GCLDLILKWLTLRFFDTNTSVLMKALEYLKLLFTLLSE
>zyg_3_T.castaneum
ANLDLILKWLTLRFFDTNPSVVFKGLEYLHSVFNVLIE
>zyg_3_A.mellifera
SNLDLILKWLTLRFFDTNPSVLLKGLEYLRMVFNLLIE
>zyg_3_D.melanogaster
CNLDLILKWLTLRFYDTNPSVLIKGLEYLVQVFQVLID
>zyg_3_A.aegypti
CNLDLIMKWLSLRFYDTNPSVLLKGLDYLNLVFAMLIE
>zyg_3_A.gambiae
CNLDLVMKWLSLRFYDTNPSVLLKGLEYLNQVFQRLVD
>zyg_4_C.elegans 24
EEVSAFVPYLLLKTGEAKDNMRTSVRDIVNVLSDVVGP
>zyg_4_O.sativa
AEAAIFLPCLMEKSGHNIEKVREKMGELIKQMVNIYSL
>zyg_4_N.tabacum
AEAAIFLPCLVEKSGHNIEKVREKMRELTKQIIHAYSA
>zyg_4_A.thaliana
AEAAIFLPCLAEKLGHNIEKVREKMRELMKQIIQAYSV
>zyg_4_O.tauris
QEAAILLPVFVEKSGHNIETIRDKFRKISRAIPTVYLA
>zyg_4_X.laevis
MEGTSFLPYLMLKVGEPKDIVRKDVRAILTKMCQVYPA
>zyg_4_G.gallus
NEASSFIPYLIIKVGEPKDVIRKDVRAILNRMCLIYPA
>zyg_4_M.musculus
NEASSFIPYLILKVGEPKDVIRKDVRAILNRMCLVYPA
>zyg_4_H.sapiens
NEASSFIPYLVVKVGEPKDVIRKDVRAILNRMCLVYPA
>zyg_4_C.familiaris
NEASSFIPYLILKVGEPKDVIRKDVRAILNRMCLVYPA
>zyg_4_T.castaneum
NEASAFLPYLVIKIGDAK--FCSGVRSLLKQVCHVYPV
>zyg_4_A.mellifera
NEAASFIPYLIIKIGDPKDAVRNGVRALFKQIALVYPV
>zyg_4_D.melanogaster
NEGSSFVPHLLLK-ANPKDAVRNGVRRVLRQVILVFPF
>zyg_4_A.aegypti
NEGSSFVPHLLTKIGDPKDVVRNGVRTLLRQICLVYPF
>zyg_4_A.gambiae
IEGSSFVPHLLIKIGDPKDVVRNGVRSLLRQICLLYPF
>zyg_5_C.elegans 0.77
VGPLKMTPMLLDALKSKNARQRSECLLVIEYYITNAGI
>zyg_5_O.sativa
YSLPKLLPYILEGLRSKNNRTRIECVDIIGYFMDHHGT
>zyg_5_N.tabacum
YSAAKTFPYILEGLRSRSNRTRIECADLVGYLLDNHEA

>zyg_5_A.thaliana
YSVGKTYPYILEGLRSKNNRTRIECTDLIGYLLETCGT
>zyg_5_O.tauris
YLASKFVGYLTTGMVETKSRTRAECLDEVSRLIERYGM
>zyg_5_X.laevis
YPASKMFNFVMEGTKSKNSKQRAECLEELGCLVESYGM
>zyg_5_G.gallus
YPASKMFTFIMEGTKSKNSKQRAECLEELGCLVESYGM
>zyg_5_M.musculus
YPASKMFPFIMEGTKSKNSKQRAECLEELGCLIESYGM
>zyg_5_H.sapiens
YPASKMFPFIMEGTKSKNSKQRAECLEELGCLVESYGM
>zyg_5_C.familiaris
YPASKMFPFIMEGTKSKNSKQRAECLEELGCLVESYGM
>zyg_5_T.castaneum
YPVARLFTYIMEGVKSKNARQRAECLEAMGSIIQDHGI
>zyg_5_A.mellifera
YPVSKLFSYVMEGLKSKNARQRTECLDQLGSLIENYGL
>zyg_5_D.melanogaster
FPFVKVFGYVMEGLKSKNARQRTECLDELTFLIESYGM
>zyg_5_A.aegypti
YPFAKVFVFVMDALKSKNARQRAECLDELGYLIETYGL
>zyg_5_A.gambiae
YPFAKVFVFIMDALKSKNARQRAECLDELGYLIETYGL

ARM repeat reference alignments	current HHpred E-values
>cat_2_M.musculus	4.3E-03
REGLLAIFKSGGIPALVKMLGSPVDSVLFYAITTLHNLLL	
>cat_2_P.dumerilii	
RQGLLAIFKSGGIPALVKLLGSPVESVLFYAITTLHNLLL	
>cat_2_A.californica	
RQGLLAIFKSGGIPALVKLLSSPLESVLFYAITTLHNLLL	
>cat_2_S.purpuratus	
RAGLLQIFKSGGIPALIKLLSSPVESVLFYAITTLHNLLL	
>cat_2_D.melanogaster	
RQGLLAIFKSGGIPALVKLLSSPVESVLFYAITTLHNLLL	
>cat_2_C.intestinalis	
KQGLLSIFKSGGIPALVKMLSSPIESVVFYAITTLHNLLL	
>cat_2_C.elegans	
RGGPLLIFRSGGLAEIIRMLYDSLESVVHYAVTTLRNLLM	
>cat_3_M.musculus	0.33
EGAKMAVRLAGGLQKMVALLNKTNVKFLAITTDCLQILAY	
>cat_3_P.dumerilii	
EGSKMAVRLAGGLQKMVALLQRNNVKFLAITTDCLQVLAY	
>cat_3_A.californica	
EGSKMAVRMAGGLQKMVSLLQRNNVKFLAITTDCLQILAY	
>cat_3_S.purpuratus	
EGSKMAVRLAGGLQKMVALLSRNNPKFLAITTDCLQILAY	
>cat_3_D.melanogaster	
DGSKMAVRLAGGLQKMVTLLQRNNVKFLAIVTDCLQILAY	
>cat_3_C.intestinalis	
EGAKEAVRLAGGLQKMVYLLSRDNVKFLAIDTDCLQILAY	
>cat_3_C.elegans	
SDSRAQARALNAVEALTPHLHKTNPKLLAQVADGLYFLLI	
>cat_4_M.musculus	8.8
QESKLIILASGGPQALVNIMRTYTEKLLWTTSRVLKVLSV	
>cat_4_P.dumerilii	
QESKLIILASGGPGELVRIMRSYTEKLLWTTSRVLKVLSV	
>cat_4_A.californica	
QESKLIILASGGPGELVRIMKSYTEKLLWTTSRVLKVLSV	
>cat_4_S.purpuratus	
QESKLIILASGGPAALVHIMRTYEEKLLWTTSRVLKVLSV	
>cat_4_D.melanogaster	
QESKLIILASGGPNELVRIMRSYDEKLLWTTSRVLKVLSV	
>cat_4_C.intestinalis	
QESKLIILASNGPQELVRIMRTYDEKLLWTTSRVIKVLSV	
>cat_4_C.elegans	
APSKITFLSLLGPQILVSILREYSRKLIYTVVRCIRSLSV	
>cat_5_M.musculus	4E-03
SSNKPAIVEAGGMQALGLHLTDPSQRLVQNCLWTLRNLSD	
>cat_5_P.dumerilii	
SSNKPAIVEAAGMQALAMHLGHQSQRLVQNCLWTLRNLSD	
>cat_5_A.californica	
SSNKPAIVEAGGMQALAMHLGHQSQRLVQNCLWSLRNLSD	
>cat_5_S.purpuratus	
HNNKPGIVEAGGMSALGLHLGHQSNRLVQNCLWTLRNLSD	
>cat_5_D.melanogaster	
SSNKPAIVDAGGMQALAMHLGNMSPRLVQNCLWTLRNLSD	
>cat_5_C.intestinalis	
SSNKPAIVEAGGMQALGMHLGSPSQRLLQNCLWSLRNLSD	
>cat_5_C.elegans	
PSNKPALISLGCLPALYVELCTAKERSQTAILVAMRNLSD	
>cat_6_M.musculus	0.59
DAATKQEGMEGLLGTLVQLLGSDDINVVTCAAGILSNLTC	
>cat_6_P.dumerilii	
DAATRCDNMEGLLQMLVQVLSSNDINMVTCSAGILSNLTC	

```
>cat_6_A.californica
DAATKMDQLDHLLQMLVQLLSSNDINVVTCAAGILSNLTC
>cat_6_S.purpuratus
DCHRGTDDIEPLLQMLVQLLASNDINVVTCACGILSNLTC
>cat_6_D.melanogaster
DAATKVEGLEALLQSLVQVLGSTDVNVVTCAAGILSNLTC
>cat_6_C.intestinalis
DAGTKQDHVENLLQMLVQLLSSNDINVVTCAAGILSNLTC
>cat_6_C.elegans
DSATNEENLTQLIIKLLEIIRVANDGMTACACGTLSNLTC
>cat_7_M.musculus                              0.028
YKNKMMVCQVGGIEALVRTVLRAGEDITEPAICALRHLTS
>cat_7_P.dumerilii
QKNKVIVCQVGRIEALVRTILQGGEDITEPAVCALRHLTS
>cat_7_A.californica
HRNKVMVCQVNGIEALVRTIMQAGEDITEPAVCALRHLTS
>cat_7_S.purpuratus
TRNKMIVSQMAGVEALVQTLMKAGEEITEPAVCALRHVTS
>cat_7_D.melanogaster
QRNKATVCQVGGVDALVRTIINAGEEITEPAVCALRHLTS
>cat_7_C.intestinalis
MSNKTRVCQVGGIEALVRTVLQAGEDITEPTVCALRHLTS
>cat_7_C.elegans
TRNKQTVCSHGGIDALVTAIRRLPEEVTEPALCALRHCTA
>cat_8_M.musculus                              0.4
EMAQNAVRLHYGLPVVVKLLHPPSWPLIKATVGLIRNLAL
>cat_8_P.dumerilii
EMAQNAVRLHYGLPVLVKLLHPPSWPLIKAVIGLIRNLAL
>cat_8_A.californica
EMAQNAVRLHYGLPVLVKLLHPPSWPLIKAVVGLIRNLAL
>cat_8_S.purpuratus
EMCQNTVRLNYGIPVIVKLLHPPSWPLIKATVGLIRNLAL
>cat_8_D.melanogaster
ELAQNAVRLNYGLSVIVKLLHPPSWPLIKAVIGLIRNLAL
>cat_8_C.intestinalis
EMAQNAVRLHYGLPVLVKLLHPPSWPLIKAVVGLIRNLAL
>cat_8_C.elegans
EEAQSELRFCQAFPVILDQLETL-TPVIKAALGVIRNSAL
>cat_9_M.musculus                              0.022
PANHAPLREQGAIPRLVQLLVRAHEEIVEGCTGALHILAR
>cat_9_P.dumerilii
PANHAPLREHGAIPRIVQLLIRAHEEIVEGTVGALHILAR
>cat_9_A.californica
PANHAPLREHGAMPRIVQLLIRAHEEIVEGTVGALHILAR
>cat_9_S.purpuratus
SANHAPLREQGALHRLVQLLMRAHEDIVEGTTGALHILAR
>cat_9_D.melanogaster
PANHAPLREHGAIHHLVRLLMRAFEEIVEGTVGALHILAR
>cat_9_C.intestinalis
AANHAALREHGAIPRLVQLLMRAHEEIVEGTTGTLHILAR
>cat_9_C.elegans
ELTQEQTANGHAVSLTMDILRRAIWGVIEGAVSALHQLAN
>cat_10_M.musculus                             0.021
-DVHNRIVIRGTIPLFVQLLYSPIENIQRVAAGVLCELAQ
>cat_10_P.dumerilii
-EAHNRAVIRGCIPLFVQLLYSPIENIQRVAAGVLCELAA
>cat_10_A.californica
-EAHNRAVIRGCIPLFVQLLYSPIENIQRVAAGVLCELAA
>cat_10_S.purpuratus
-DSHNRALIQGCIPLFVQLLYSNIENIQRVAAGVLSELSL
>cat_10_D.melanogaster
-ESHNRALIRQVIPIFVRLLFNEIENIQRVAAGVLCELAA
```

>cat_10_C.intestinalis
-EPHSRSVIRGTIPLFVQLLYSQVENIQRVAAGVLCELAQ
>cat_10_C.elegans
AACCDDIGQVGPFLDLLHRLLAHPEVLEREILGLLYQLSK
>cat_11_M.musculus 0.025
KEAAEAIEAEGATAPLTELLHSRNEGVATYAAAVLFRMSE
>cat_11_P.dumerilii
KEGAEIIEQEGATAPLTELLHSRNEGVATYAAAVLFRMSE
>cat_11_A.californica
KEGAEMIEQEGATAPLTELLHSRNEGVATYAAAVLFRMSE
>cat_11_S.purpuratus
KQGAEMIEQEGATAPLTELLHSRNEGVATYAAAVLYRMSD
>cat_11_D.melanogaster
KEGAEIIEQEGATGPLTDLLHSRNEGVATYAAAVLFRMSE
>cat_11_C.intestinalis
KESADLIENEGASAPLTELLHSKNEGVATYAAAALFRMSE
>cat_11_C.elegans
PDGARAVESTGVSALLMESRGSQYKSVVTYANGVLSNLKR
>hspb_1_H.sapiens 0.097
MDNAADFCQLSGMHLLVGRYLEAGAGLRWRAAQLIGTCSQ
>hspb_1_X.laevis
LDNASDFCKLGGMNLLLSRYVNCPAELRWRSADLIGICSQ
>hspb_1_D.rerio
LDNARDLMKLGGLDLCLSRCLCHTAGIRWRAAQLIASSAQ
>hspb_1_A.mellifera
IDIANDFYKIGGFSIFGPCLNSPHSSIRWRAADVIAELAQ
>hspb_1_T.castaneum
IDTANDFHKIGGFVIVSPCLKCKSPKVRAQVCNLLAELCQ
>hspb_1_A.aegypti
IDAANDFYKVGGFVIIQPGLTSSNTDVRSGTLRLIAELSQ
>hspb_1_O.sativa
IDMANDLHSIGGLDPLLGYLKNSHAGIRAKAAEVVSTIVQ
>hspb_1_A.thaliana
IDMANDLHSIGGLVPLLSFLKNSHANIRAKAADVVSTIVQ
>hspb_1_U.maydis
IDNANNMTSMKMWSPIISLLSAPEAEIQTAAAWIIGTAVQ
>hspb_1_N.crassa.
LDNANLLEELSLWSPLISLLDHEDEDMRYHAAWCLGTAVQ
>hspb_1_S.cerevisiae
LDNANNIENLKLWEPLLDVLVQTKEELRAAALSIIGTAVQ
>hspb_1_S.pombe
IDNANNLVPLQLWPRLLKQLESPESTLRRLAAWTIATAVQ
>hspb_1_D.discoideum
IDNAGDFIKIGGIPVLIKLLTPLSDKVRADAATCLSTITQ
>hspb_1_D.melanogaster
IDNALMFIDNGGLDDVLLPIVVNDTSLRVSAMRVLGSLAS
>hspb_1_Y.lipolytica
LDNANMMKNLKLWEPLLAQLSSPHPSLQKLAAWVVATATQ
>hspb_2_H.sapiens 2.0E-03
AAIQEQVLGLGALRKLLRLLDRDADTVRVKALFAISCLVR
>hspb_2_X.laevis
PFVQETALRLGAVKILLQLLDLDSDQVRIKALFAISCLVR
>hspb_2_D.rerio
PEVQFYLLNQGALLTLLQLADNDPSTVRVKALYAVSCLVR
>hspb_2_A.mellifera
PFCQERFLETGLFPILLNMIDTDPETVRIKALYAVSCIVR
>hspb_2_T.castaneum
AYCQRVVLESGIMPILVEIVEQDPVSVVVKALYAISCIVR
>hspb_2_A.aegypti
PFCQQHLLQANTLPQIIELLSDVP-PVATQAMHAISCMVR
>hspb_2_O.sativa
PKSQQLVMESNGLEPLLTNFSSDATNSRTKALGAISSLIR

```
>hspb_2_A.thaliana
PRSQELVMETNALESLLSNFTSDTIHARTQALGAISSLIR
>hspb_2_U.maydis
DKAQMAVLDFHPVAALLDLLHSHVDEVRAKAMYALSGLLK
>hspb_2_N.crassa.
QKTQERLLAMGGVPKLVDLAMKEGEKVRRKATYALSSAVR
>hspb_2_S.cerevisiae
LDSQNNFMKYDGLRSLIEIASDKTLDVRTKAFYALSNLIR
>hspb_2_S.pombe
PKSQQALIENDGLKILFGALKKEDDETKNKVLYAITSELK
>hspb_2_D.discoideum
ETIQAYLHSLGVLDLAVKQLQKEIPLVREKFLSLISSLLS
>hspb_2_D.melanogaster
PKAQIKVFEKNFGSHLAQILTSSGVGEISAALHAFGALLR
>hspb_2_Y.lipolytica
PKSQEALVEQGGIKKLVDLTSHDDPEVVVKSLFALASAIR
>hspb_3_H.sapiens                              0.18
EAGLLQFLRLDGFSVLMRAMQQQVQKLKVKSAFLLQNLLV
>hspb_3_X.laevis
EEGLAEFLKQDGFSVLMRAMQSDAQKLKVKSAFLLQNLLI
>hspb_3_D.rerio
EAGLKDFLSHDGFSVLMRGMQSDSEKLRTKSAFLLLNLLN
>hspb_3_A.mellifera
PMSLKYMDINDGYSILLRAMQSSIKKLQIKSAFLLSSLCS
>hspb_3_T.castaneum
TGACAQFIQYKGVQVFLEALKRNEEKINTKICFLLRALCS
>hspb_3_A.aegypti
EPCLAAFIDMGGLECILGCIQTDNEKLRIKSSFLMSNLCT
>hspb_3_O.sativa
QPGVAAFRLGNGYSALRDALGSDDARLQRKALHLLQYLLH
>hspb_3_A.thaliana
KPGVTAFKLANGYAGLRDALASDSVRFQRKALNLLQYLLQ
>hspb_3_U.maydis
PAAMHQFDQLDGWNMLNMALVDPNLGLRRKTAFLINALLL
>hspb_3_S.cerevisiae
KDISEKFFKLNGLDCIAPVLSDNTPKLKMRAIALLTAYLS
>hspb_3_S.pombe
EAGIALLDKIPSWEMLIEILELKHSVMTKRVIFFFYALLI
>hspb_3_D.discoideum
-----EIRKCT-DKSILNTIVELSTTFLSPTVELTVQLNN
>hspb_3_D.melanogaster
PLAQQRVLSTSGTQALIKVLQSPDLRSKAKVVTLISDLVL
>hspb_3_Y.lipolytica
DDAYKLFESADGLKKVVGHLKP--AQVKSKTLGVLTGILE
>impa_2_S.cerevisiae                           3.0E-06
RPPIDVVIQAGVVPRLVEFMRENQEMLQLEAAWALTNIAS
>impa_2_Y.lipolytica
SPPINEVIQCDVVKYFVEFLKSPHNLLQFEAAWALTNIAS
>impa_2_N.crassa
NPPIEEVIKTGVVGRFVEFLRSPHTLVQFEAAWALTNIAS
>impa_2_S.pombe
HPPIDQVIACGVVDRFVQFLESEHHLLQFEAAWALTNIAS
>impa_2_C.neoformans
NPPIDKVIQCGVVPRFVEFLSSTNSMLQFEAAWALTNIAS
>impa_2_O.sativa
SPPIEEVINTGVVPRFIAFLQREDPQLQFEAAWALTNIAS
>impa_2_B.napus
SPPIDEVIKAGVIPRFVEFLRRQDPQLQFEAAWALTNVAS
>impa_2_M.musculus
PPIDEVINTPGVVDRFVEFLKRNECTLQFEAAWALTNIAS
>impa_2_N.vectensis
NPPIDDVIKCGVIPKFVEFLQREDSALQFEAAWALTNIAS
```

```
>impa_2_D.rerio
PPIDEVIGTPGVVNRFVEFLRRSDCTLQFEAAWALTNIAS
>impa_2_A.mellifera
NPPIDEVVKTGIVPKFVEFLENNACTLQFEAAWALTNIAS
>impa_2_O.lucimarinus
SPPIDQVIETGATPFFVEFLKRTDPKLQFEAAWALTNIAS
>impa_2_D.discoideum
SPPIEEVIKTGIVPRLVKFLYMQDPQLQFEAAWALTNIAS
>impa_2_D.melanogaster
NPPIEEVIQKGIVPQFVTFLRNSAATLQFEAAWTLTNIAS
>impa_2_A.californica
NPPIDDLITSGILPILVNCLSRDDPSLQFEAAWAITNIAS
>impa_3_S.cerevisiae                    7.2E-08
SAQTKVVVDADAVPLFIQLLYTGSVEVKEQAIWALGNVAG
>impa_3_Y.lipolytica
SEQTKVVVDSGAVPLFVHLLDSPETNVREQAVWALGNIAG
>impa_3_N.crassa
ATQTQVVIEAGAVPIFVELLGSPEPDVREQAVWALGNIAG
>impa_3_S.pombe
TDQTRIVVDSGAVPRFIQLLSSPEKDVREQVVWALGNIAG
>impa_3_C.neoformans
SEHTQVVIGAGAVPHFIALLSSSVLDVREQAVWALGNIAG
>impa_3_O.sativa
SDNTKVVVESGAVPIFVKLLSSPSEDVREQAVWALGNVAG
>impa_3_B.napus.
SDHTRVVIEHGAVPIFVELLSSASDDVREQAVWALGNVAG
>impa_3_M.musculus
SQQTKIVIEAGAVPIFIELLNSDFEDVQEQAVWALGNIAG
>impa_3_N.vectensis
SMQTRETVNAGAVPCFIKLLESPKEEVQEQAVWALGNIAG
>impa_3_D.rerio
FQHTKVVIETGAVPIFIELLNSEYEDVQEQAVWALGNIAG
>impa_3_A.mellifera
SQQTRVVVDAGAVPIFISLLGSEYEDVQEQAVWALGNIAG
>impa_3_O.lucimarinus
SEHTAIVIDHGAVPIFIALLGSDNPDVREQAVWALGNIAG
>impa_3_D.discoideum
PEQTRVVIENGAIQVFVLLLSSPHDDVREQAVWALGNIAG
>impa_3_D.melanogaster
SQQTKVVIEAGAVPIFIDLLSSPHDDVQEQAVWALGNIAG
>impa_3_A.californica
SAQTQAVVNAGAVPFFLQLLRSPHQNVCEQAVWALGNIIG
>impa_4_S.cerevisiae                    1.2E-06
TDYRDYVLQCNAMEPILGLFNSNKPSLIRTATWTLSNLCR
>impa_4_Y.lipolytica
PQCRDYVLKCDALQPLINIATNTKLSMIRNATWTLSNFCR
>impa_4_N.crassa
PQCRDYVLSCGALRPLLTLLGDSRLSMLRNATWTLSNFCR
>impa_4_S.pombe
SACRDYVLGNGVLQPLLNILQSSAVSMLRNATWTLSNLCR
>impa_4_C.neoformans
PKCRDYVLGEGALQPLLGLLNENHLSMIRNATWTLSNFCR
>impa_4_O.sativa
PKCRDLVLASGGLYPLLQQLNEHALSMLRNATWTLSNFCR
>impa_4_B.napus
PNCRNLVLSCGALAPLLSQLNENSLSMLRNATWTLSNFCR
>impa_4_M.musculus
SLCRDYVLNCSILNPLLTLLTKSTLTMTRNAVWALSNLCR
>impa_4_N.vectensis
ADCRDYVLNCGVLTPFLNLLTKSTLSMTRNLVWALSNLCR
>impa_4_D.rerio
AECRDYVLNCGILPSLQQLLAKSNLTTTRNAVWALSNLCR
```

>impa_4_A.mellifera
PECRDHVLDRGILTPLLQLLSKATLSMTRNAVWALSNLCR
>impa_4_O.lucimarinus
PRCRDLVLHANALHPLLAQLNAEAIQMLRNATWTLSNFCR
>impa_4_D.discoideum.
HYCRDLVLSHNALPPLLSLLQNPAVSMVRNATWTLSNFCR
>impa_4_D.melanogaster
PMCRDHLLGSGILEPLLHVLSNSDITMIRNAVWTLSNLCR
>impa_4_A.californica
PECRNYVISLGAVQHLLTFINPNILPFLRNVAWVIVNLCR
>impa_5_S.cerevisiae 1.6E-03
KPQPDWSVVSQALPTLAKLIYSMDETLVDACWAISYLSDG
>impa_5_Y.lipolytica
YPQPDWEVIKHALPALAKLIFSYDDEVLIDACWAISYLSD
>impa_5_N.crassa
TPQPDWNTIAPALPVLAKLVYSLDDEVLIDACWAISYLSD
>impa_5_S.pombe
NPPPNWSTISVAVPILAKLLYSEDVEIIVDACWAISYLSD
>impa_5_C.neoformans
NPQPEWELISPALTVLTKLIYSLDDEVLIDACWAISYLSD
>impa_5_O.sativa
KPQPNFEQVKPALSALQRLIHSQDEEVLTDACWALSYLSD
>impa_5_B.napus
KPPTPFEEVKPALPVLRQLIYLNDEEVLTDACWALSYLSD
>impa_5_M.musculus
NPPPEFAKVSPCLPVLSRLLFSSDSDLLADACWALSYLSD
>impa_5_N.vectensis
HPPPDFAKVSPCLPLLSRLLFSTDAEVLADTCWALSYLLD
>impa_5_D.rerio
NPPPDFAKVSPCLSVLSRLLFSSDPDVLADACWALSYLSD
>impa_5_A.mellifera
TPPPEFTKVAPCLPVLAHFLNHTDSDVLADACWALSYLSD
>impa_5_O.lucimarinus
KPQPDFSALRAALPALARLVHSNDEEVLTDACWALSYLSD
>impa_5_D.discoideum
KPQPPFEIVRASLPVLAKLIYYQDEEVLIDACWALSYLSD
>impa_5_D.melanogaster
SPPADFAKISHGLPILARLLKYTDADVQSDTCWAIGYLSD
>impa_5_A.californica
DPPPPAETIEEILPALCTLIHHSDVNILVDTVWALSYLTD
>impa_6_S.cerevisiae 9.8E-05
QEAIQAVIDVRIPKRLVELLSHESTLVQTPALRAVGNIVT
>impa_6_Y.lipolytica
TNKIQAVVDAGIPRRLIELLGHQSTSVQTPALRSVGNIVT
>impa_6_N.crassa
NDKIQAVIEAGIPRRLVELLMHASTSVQTPALRSVGNIVT
>impa_6_S.pombe
NEKIGAILDVGCAPRLVELLSSPSVNIQTPALRSVGNIVT
>impa_6_C.neoformans
NDKIQAVIESGVCRRLVDLLMHPSTAVQTPALRSVGNIVT
>impa_6_O.sativa
NDKIQAVIESGVFPRLVELLMHPSASVLIPALRTVGNIVT
>impa_6_B.napus
NDKIQAVIQAGVCPRLVELLSHPSPTVLIPALRTVGNIVT
>impa_6_M.musculus
NEKIQAVIDSGVCRRLVELLMHNDYKVASPALRAVGNIVT
>impa_6_N.vectensis
NEKIQAVIDSGVCRRLVELLDHNQNSVVSAALRAVGNIVT
>impa_6_D.rerio
NDKIQTVIDSGVCRRLVELLMHSDYKVVSPALRAVGNIVT
>impa_6_A.mellifera
NEKIQAVIDAGVCRRLVELLMHQQENVVSAALRAVGNIVT

>impa_6_O.lucimarinus
NDKIQAVIEAGVCRRLVELLASNHPSVLIPALRTVGNIVT
>impa_6_D.discoideum
NERIQEVIDAKVCRKMVELLGHPTIAVQTPALRTIGNIVT
>impa_6_D.melanogaster
NDNIQAVIDAGVCRRLVELLIHPQQNVSTAALRAVGNIVT
>impa_6_A.californica
NDQIQMVIDSGVVPFLVPLLCHQDVKLQTAALRAVGNIVT
>impa_7_S.cerevisiae 5.2E-07
DLQTQVVINAGVLPALRLLLSSPKENIKKEACWTISNITA
>impa_7_Y.lipolytica
DVQTQIVINAGALPALLQLLTAPKDSIRKEACWTISNITA
>impa_7_N.crassa
DVQTQVIINCGALPCLLSLLSSNKDGIRKEACWTISNITA
>impa_7_S.pombe
DAQTQIIIDCGALNAFPSLLSHQKENIRKEACWTISNITA
>impa_7_C.neoformans
DLQTQVVISSGALPALLSLLSSPKEGIRKEACWTISNITA
>impa_7_O.sativa
DMQTQCVIDHQALPCLLNLLTNNHKSIKKEACWTISNITA
>impa_7_B.napus
DSQTQFIIDSGVLPHLYNLLTQNHKSIKKEACWTISNITA
>impa_7_M.musculus
DIQTQVILNCSALPCLLHLLSSSKESIRKEACWTISNITA
>impa_7_N.vectensis
DIQTQVILNCHALPNLLHLLSSTKESIRKEACWTISNITA
>impa_7_D.rerio
DIQTQVILNCSALPCLLHLLSSPKESIKKEACWTVSNITA
>impa_7_A.mellifera
DVQTQIVLNCSALHCLYHLLNSSQESIRKEACWTISNITA
>impa_7_O.lucimarinus
DYQTQIIINCHALKALLGLLAGDYKSIKKEACWTISNITA
>impa_7_D.discoideum
DNQTQIVLSVQALSHLLNLLQSPKRAIRKEACWTISNITA
>impa_7_D.melanogaster
DQQTQVILGYNALTCISHLLHSTAETIKKESCWTISNIAA
>impa_7_A.californica
DEQTQAVLNCNALQHFSSLLTHPKEKINKEAVWFLSNITA
>impa_8_S.cerevisiae 1.1E-05
TEQIQAVIDANLIPPLVKLLEVAEYKTKKEACWAISNASS
>impa_8_Y.lipolytica
STQIQSVIDSNLIPPLIQLLSTGEVKTKKEACWAISNATS
>impa_8_N.crassa.
SAQIQSVIDANIIPPLIHLLSHADLKTRKEACWAISNATS
>impa_8_S.pombe
TQQIQAIIESNLIPPLVHLLSYADYKTKKEACWAISNATS
>impa_8_C.neoformans
PMQIQAIIDANIVPPLINILANADFKTKKEACWAISNATS
>impa_8_O.sativa
REQIQAVINANIIAPLVHLLQTAEFDIKKEAAWAISNATS
>impa_8_B.napus.
KVQIEAVVGAGIVLPLVHLLQNAEFDIKKEAAWAISNATS
>impa_8_M.musculus
RAQIQAVIDANIFPVLIEILQKAEFRTRKEAAWAITNATS
>impa_8_N.vectensis
RLQIQAVIDANIIPMLIDVLNKAEFKTRKEAAWAIANATS
>impa_8_D.rerio
RAQIQAVIDSNVFPVLIEILQKAEFRTRKEAAWAITNATS
>impa_8_A.mellifera
PQQIQAVIDAGIFPVLIDILAKAEFKIRKEAAWAITNATS
>impa_8_O.lucimarinus
KDQIQSIIDEQMVPPLVELLANAEFDIKKEAAWAISNATS

```
>impa_8_D.discoideum
KNQIQQVIDANIIPSLVYLLANAEFEIQKEAAWAISNATS
>impa_8_D.melanogaster
REQIQALINEH-IPQLMVIMQTAEFKTRKEAAWAITNATS
>impa_8_A.californica
QQQVQAVIDASLIPLIIHHLNRGDFQTQKEAAWAISNLTI
>impa_9_S.cerevisiae                          3.1E-03
PDIIRYLVSQGCIKPLCDLLEIADNRIIEVTLDALENILK
>impa_9_Y.lipolytica
PDQIRYLVQQGCIKPLCDLLGSMDNKIIQISLDALENILR
>impa_9_N.crassa
PDQIRYLVAQGCIKPLCDLLACPDNKIIQVALDGLENILK
>impa_9_S.pombe
PDQIRYLVSQGVIKPLCDMLNGSDNKIIQVALDAIENILK
>impa_9_C.neoformans
PNQIRYLVSQGCIKPMCDLLTSMDNKIIQVALDGLENILK
>impa_9_O.sativa
HDQIKYLVAQGCIKPLCDLLVCPDPRIVTVCLEGLENILK
>impa_9_B.napus
HEQIQYLVAQGCIKPLCDLLICPDPRIVTVCLEGLENILK
>impa_9_M.musculus
PEQIRYLVSLGCIKPLCDLLTVMDSKIVQVALNGLENILR
>impa_9_N.vectensis
AEQIRYIVSQGAIRPLCDLLTVLNPKIVQVGLNALDNILK
>impa_9_D.rerio
PEQIRYLVSLNTIKPMCDLLTVMDSKIVQVALNGLENILR
>impa_9_A.mellifera
PEQIRYIVVEGCIPPLCNLLTVMDPKIVQVALSGLENILR
>impa_9_O.lucimarinus
HQQIKYLVSQGCIKPLCDLINCSDARIVTVALEGLENILK
>impa_9_D.discoideum
PQQIHFLVSQGCVKPLCDLLKVSDPRIINVALEGIENILV
>impa_9_D.melanogaster
DEQIHYLVQVGCVPPMCDFLTVVDSDIVQVALNALENILK
>impa_9_A.californica
KEQVAYVVEMGVLPPFCNLLSVKDAQVVNVVLDGINNILK
>plak_1_H.sapiens_1                           4.8E-06
ESAKQQVYQLGGICKLVDLLRSPNQNVQQAAAGALRNLVF
>plak_1_G.gallus_1
ESAKQEVYRLGGIAKLVELLRSTNQNVQRAAAGALRNLVF
>plak_1_M.muscilus_2
SEARKRVNQLRGIPKLLQLLKLQNEDVQRAACGALRNLVF
>plak_1_X.laevis_1
SEAKKQARSVQAIPKLVKLFNCDNQEVQRHATGAMRNLIY
>plak_1_X.laevis_3
GEAKKQARSLQAIPKLVKLLNCDNQEVQRHATGAMRNLIY
>plak_1_M.musculus_4
NKVKMEVYRLGGIKHLVDLLDHRVLEVQKNACGALRNLVF
>plak_1_H.sapiens_3
AAAKKQARSLQAVPRLVKLFNHANQEVQRHATGAMRNLIY
>plak_2_H.sapiens_1                           1.0E-04
TTNKLETRRQNGIREAVSLLRRTGAEIQKQLTGLLWNLSS
>plak_2_G.gallus_1
PTNKIETRRQNGIRECVSLLRRTGTEIQKQLTGLLWNLSS
>plak_2_M.muscilus_2
NDNKLEVAELNGVPRLLQVLKQTRLETKKQITGLLWNLSS
>plak_2_X.laevis_1
PENKMALVEENGIYELLTALEEPDDELRKNVTGILWNLSS
>plak_2_X.laevis_3
PENKMALVEENGIYELMTALEEPDDELRKNVTGILWNLSS
>plak_2_M.musculus_4
DENKIAMKNVGGIPALLRLLRKSIAEVRELVTGVLWNLSS
```

>plak_2_H.sapiens_3
ADNKLALVEENGIFELLRTLREQDDELRKNVTGILWNLSS
>plak_3_H.sapiens_1 5.7
IADALPVLADRVIIPFSGWCDGNSPEVFFNATGCLRNLSS
>plak_3_G.gallus_1
IQEALPVLTDCVIIPFSGWSDGGCPEVFFNATGCLRNLSS
>plak_3_M.muscilus_2
ITEALLTLTESVIIPFSGWPEGDYFDIFYNVTGCLRNMSS
>plak_3_X.laevis_1
ARDTLNPLTQKVLSPLSGTAGSAVSEIFYNSTGFLRNLSS
>plak_3_X.laevis_3
ARDTLNQLTQRVLSPLSGTAGSAVAEIFYNATGYLRNLSS
>plak_3_M.musculus_4
IRDALSTLTNTVIVPHSGWNNSSFSLVLRNTTGCLRNLSS
>plak_3_H.sapiens_3
ARDTLEQLTDLVLSPLSGAGGPPLAEIFYNATGFLRNLSS
>plak_4_H.sapiens_1 2.8
DAGRQTMRNYSGLIDSLMAYVQNCDKSVENCMCVLHNLSY
>plak_4_G.gallus_1
DMGRQTMRNYPGLIDSVMTYAQNCDKSVENCICILHNLSY
>plak_4_M.muscilus_2
PDGRKMMRRCDGLIDSLVHYVRGTDKATENCVCILHNLSY
>plak_4_X.laevis_1
PETRQRMRECPGLLDSLVSYISNADKSVENAVCVLRNLSY
>plak_4_X.laevis_3
PETRQKMRECPGLLDSLVSYTNNADKSVENVVCVLRNLSY
>plak_4_M.musculus_4
EEARKQMRSCEGLVDSLLYVIHTCSKTVENCVCTLRNLSY
>plak_4_H.sapiens_3
QATRQKMRECHGLVDALVTSINHADKSVENAVCVLRNLSY
>plak_5_H.sapiens_1 0.27
ETNPKGSGWLYHSDAIRTYLNLMGDATLEACAGALQNLTA
>plak_5_G.gallus_1
DFKPKGPSWLYHSDAIRTYLSLMDDATLEACAGALQNLTA
>plak_5_M.muscilus_2
RSNPHGIEWLWHSIVIRMYLSLIANYTQEASLGALQNLTA
>plak_5_X.laevis_1
SRDPKGMELLWHPQIVNLYNRLLQKYTTEAAAGALQNITA
>plak_5_X.laevis_3
SRDPKGMELLWHPQIVNLYNRLLQKYTTEAAAGALQNITA
>plak_5_M.musculus_4
SKSPKGVEMLWHPSVVKPYLTLLAPATLEGSAGSLQNLSA
>plak_5_H.sapiens_3
SKDPKGLEWLWSPQIVGLYNRLLQRHTTEAAAGALQNITA
>plak_6_H.sapiens_1 6.2E-03
GMSQLIGLKEKGLPQIARLLQSGNSDVVRSGASLLSNMSR
>plak_6_G.gallus_1
AMSQLIGVKEKGLPRIARLLQSNSSEVVRSGASLLSNMSR
>plak_6_M.muscilus_2
LVARMVVQKENGLQHTRKMLHVGDPSVKKTAVSLLRNLSR
>plak_6_X.laevis_1 dir
VLSQVALDQERILNPVLDRLRTADHNQLRSLTGLIRNLSR
>plak_6_X.laevis_3
VLSQVALDQERILNPVLDRLRTADHNQLRSLTGLIRNLSR
>plak_6_M.musculus_4
YI-RAAVRKEKGLPILVELLRMDNDRVVSSVATALRNMAL
>plak_6_H.sapiens_3
VLSRLALEQERILNPLLDRVRTADHHQLRSLTGLIRNLSR
>plak_7_H.sapiens_1 0.52
HPLLHRVMGNQVFPEVTRLLTSHTEDILSSACYTVRNLMA
>plak_7_G.gallus_1
HPILHKTMAHQVLPDVSRLLSFQSGEIMTSACYTLRNLIM

>plak_7_M.muscilus_2
NLSLQNEIAKETLPDLVSIIPDTVIETTASACYTLNNLMQ
>plak_7_X.laevis_1
HAKNKDEMSTKLVSHLLEKLPADSADVLVNIIAILNNLTT
>plak_7_X.laevis_3
HAKNKDEMSTKLVSHLLEKLPADSAEVLVNIIAILNNLTV
>plak_7_M.musculus_4
DVRNKELIGKYAMRDLVNRLPGGNDETVAAICCALHEVTS
>plak_7_H.sapiens_3
NARNKDEMSTKVVSHLIEKLPGSVAEVLVNIIAVLNNLVV
>vac_1_S.cerevisiae 2
GDGLSSSNNAHTLIPLIHLLSTSDEDCKKSVQNLIAELLS
>vac_1_M.musculus
SEDKQEMLQTE---QCAKTFINLMEQTVQYILTMVDDMLQ
>vac_1_C.elegans
EAERSTVLSVFAVYAFVHLMSQISDDYVRYTLTLIDDMLR
>vac_1_N.crassa
DVRRQVIEANLGYKTLFAGNNGQRPDVCQYLLVLLSDLLD
>vac_1_D.rerio
EDKQAILTNEGCAKTFLNLMAHISEQTVQYILTLIDDTLQ
>vac_1_X.tropicalis
EDKQIILTNEGCAKTFINLMTHISEQTVQYILTMVDDMLQ
>vac_1_S.pombe
SAYVKTAEEDTYSNLFLKLLSMKDPDVVNFALVKLADTLL
>vac_1_Y.lipolytica
EARKETVKKDLYATSFVSLLRKIPEDVAKYILSFVASLVQ
>vac_1_D.melanogaster
SRASFLAQNSSVVKTLLNLVSHLSDSTIQYILVLLDDLLQ
>vac_1_T.castaneum
AKREKLLQTDRCAQTFLNLLGHVSDQTLQYILVLIDDMLQ
>vac_1_C.neoformans
AQRPTVLATQGYAKLYIDLLRKLQVDTVQAVLVSISDML-
>vac_1_O.sativa
SQRGPLLDEDGYVRVFLNILRNSSEDTVEYVLALIDEMLA
>vac_1_A.thaliana
SARAQLLDEDGYVHLFVSILRDIFEETVEYVLALIYEMLS
>vac_1_D.discoideum
EKKEKFAANSAYVNFFVNFINSTSIEIIQYLLTLINEIIE
>vac_1_T.thermophila
SAQRELIKNSAYFSTFFEILTEAYQKIMRYLLPTIDGIIM
>vac_2_S.cerevisiae 14
GDDTVKFFQEDQLEQLFDVSLKGDQTVLISGFNVVSLLVQ
>vac_2_M.musculus
NHQRVSIFFDYTAWPYFLPMLNRQPFTVHMAARIIAKLAA
>vac_2_C.elegans
DVTRTIIFEDVKRSPFSFFMGLLHQYIVHITFSILTKMAV
>vac_2_N.crassa
VPALSKAFFQSPYKNILPLLAHSTDPIPLLTSIVLVRLMA
>vac_2_D.rerio
NHQRVNIFFDYAWSYFLPMLNRQDLFTVHMAARIIAKLAA
>vac_2_X.tropicalis
NHQRVSIFFDYAWSYFLPMLNRQDLFTVHMAARIIAKLAA
>vac_2_S.pombe
SNKFLSAFGPAKDESYINYLDDDSKLLFARVFALCSSSSP
>vac_2_Y.lipolytica
LPEFAQAMLELPFGPLLKLLESEDEQIQLLAVKSIVVLLA
>vac_2_D.melanogaster
DRSRVDLFHDTIWGPFLNLLNRQDGFIVNMSSRILAKFAC
>vac_2_T.castaneum
DRSRVEIFHEYVWGPFLNLLNRQDGFITNMTSRIIAKIAC
>vac_2_C.neoformans
NSTIPYFHNLAPYGPIVKCLSMDEEFPVLGSLRILSLLIA

```
>vac_2_O.sativa
NPKRAALFYDNIYDPFLRLLLKGNWFVQEKSCKILTQIIS
>vac_2_A.thaliana
NPTRARLFHDETYEPFLRLLWKGNWFIQEKSCKILAWIIS
>vac_2_D.discoideum
DPRAAGAFSKIPYSVFFRLLNREDAYTNLHASIALAQIMC
>vac_2_T.thermophila
RRVLQYIVQSLWLTALNVIIHNDNITVTEAALRIKACIIG
>vac_3_S.cerevisiae                          320
KLVEKLLKNN-NLINILQNIEQM--DTCYVCIRLLQELAV
>vac_3_M.musculus
-AWGKELMEGSDLNYYFNWIKTQLSQYVQCVAGCLQLMLR
>vac_3_C.elegans
VFGNIKLSGD-ELDYCMGSLKEAMNDYIVTAVRCMQTLFR
>vac_3_N.crassa
SRDESTATAE-KALPMLFSYLGSLAGLQDIGVQEYSQLLY
>vac_3_D.rerio
-AWGRDLMEGSDLNYYFNWIKTQLSQYVQCVAGCLQLMLR
>vac_3_X.tropicalis
-AWGRELMEGSDLNYYFNWIKTQLSQYVQCVAGCLQLMLR
>vac_3_S.pombe
----PCSVAK-AFTLFLEYLGKLMPLTRLFAVQCLNGVLT
>vac_3_Y.lipolytica
DSKTVDLTTEKYAAQYLQFVGLLSVQVQDVGTVSLAVLLS
>vac_3_D.melanogaster
CWGHETMPKS-DLNFYLQFLKDQLNEYIQSVARCLQMMLR
>vac_3_T.castaneum
CWSQTPMERS-DLHFYLTWLKDQLNEYIQSVGRCLQMMLR
>vac_3_C.neoformans
--TDPKPFPN-DLVPTLLSSLQKLLPLWEVAAQVLGAVLG
>vac_3_O.sativa
NSKSKLTSTQDVLRGLVDWLCSQLNCSVPTAMHCLATLLR
>vac_3_A.thaliana
NAVIGNGIDD-VLKGLVEWLCAQLTRGVPIAISCLSSLLK
>vac_3_D.discoideum
---AGKPTQN-DVESFFNWILKLLSSEVEVGLIALQSLLL
>vac_3_T.thermophila
-ELDKKIYLKEQQEFLLKLLNLQNQVEDYCLITSLAFILK
>vac_4_S.cerevisiae                          3.8
IPEYRDVIWLHFMPTLFKILQRATIQLQYHSLLLIWLLTF
>vac_4_M.musculus
-VNEYRFAWVEGVNCIMGVLSNKCFQLQYQMIFSIWLLAF
>vac_4_C.elegans
-FDPYRVSFVNGYDSLTHALYSTRFQIQYQIIFCMWLLTF
>vac_4_N.crassa
SRVCRQQFWKQTVGPLIDILRAAALQLLYHVLLVLWQLSF
>vac_4_D.rerio
-VNEYRFAWVEGVNCITAVLSNKCFQLQYQMIFCVWLLAF
>vac_4_X.tropicalis
-VNEYRFAWVEGVNCIMSVLSNKCFQLQYQMIFCLWLLAF
>vac_4_S.pombe
LKAHRYALWAECSFRLAELLRNSITQLQYYSLFCFWQLTF
>vac_4_Y.lipolytica
NRSYRPLFWKFLVPKLLDIIKADKLHLQYYTLLVIWLESF
>vac_4_D.melanogaster
VDEYRFAFVGVGISTLIRILSTRVFQVQYQLIFCLWVLTF
>vac_4_T.castaneum
IDEYRFAFVSVGISTLLSVLSGRVFQVQYQLIFCLWVLTF
>vac_4_C.neoformans
TKQFRKFVWNECLSGLIKSLKTNPPQAQYWAITCLWQLSF
>vac_4_O.sativa
EQYVRALFVQAGVKLLIPLISPASIQLLYETCLCIWLLSF
```

>vac_4_A.thaliana
EPVVRSSFVQAGVKLLVPLISPASIQLLYETCLCIWLLSY
>vac_4_D.discoideum
KDDFRIFFNNIGSALLLNILQALSIQLLYETIYAIWLLTY
>vac_4_T.thermophila
AHLVNKFIDNRGQERIYQILKKNKLQIMYYTLLVLWLLSF
>vac_5_S.cerevisiae 0.22
-VFANELVQKYDFLDLLKLVKITIEKVSRLCISIILQCCS
>vac_5_M.musculus
-QMCEHLRRYNIIPVLSDILQESVEKVTRIILAAFRNFLE
>vac_5_C.elegans
-HAAEVALSGNLIQTISGILGNCQEKVIRIVVSTLRNLIT
>vac_5_N.crassa
-DIGDDLNNEYIVLLYTHLLRLSPEKTTRLVIATLYNLLS
>vac_5_D.rerio
-QLCEQLRRYNVVPALSDILQESVEKVTRIILAAFRNLLE
>vac_5_X.tropicalis
-QMCEYLRRYNIVPVLSDILQESVEKVTRIILAAFKNLLE
>vac_5_S.pombe
-HIAQDINKRFLIKLLVQIIRSDTTKVYRLVLAILVNLID
>vac_5_Y.lipolytica
-KAAHQLVSSFLIPTLLQAARTSVEKITRLCVAIIVNVLK
>vac_5_D.melanogaster
-LLAAKMNKFSVIPILADILSDCAEKVTRIILAVFRNLIE
>vac_5_T.castaneum
-LLAEKMNKFNVIPILADILSDSVEKVTRIILAVFRNLIE
>vac_5_C.neoformans
-EVAENLDKKYVVAILTDIAKAAVEKVTRVVVATFRNLLA
>vac_5_O.sativa
-AAVDYLSTTRVMPRLVEVVKGSTEKVVRVVIMSIRNLLA
>vac_5_A.thaliana
-PAIEYLATSRTMQRLTEVVKHSTEKVVRVVILTFRNLLP
>vac_5_D.discoideum
-DIAAAYSGTGLVANLVQLVKTVAEKIVRLSLSTLRNLLN
>vac_5_T.thermophila
-KSIESFLQPNFISSMVETIQKLSEKLIRVAFQCLKNLAE
>vac_7_S.cerevisiae 0.36
SDNIDEFKKDNIFRQLIELLQAKVKIIIQVALNDITHVVE
>vac_7_M.musculus
RENAVRLNEKNLLKILTKLLEVSDPQVLAVAAHDVGEYVR
>vac_7_C.elegans
NENAHRLNDNRLLKLLVAMLEKSNPLVLCVAAHDIGEFVR
>vac_7_N.crassa
AENARKILDYEIVRKLAEIMQKPWKAVLAIACNDVGCLVR
>vac_7_D.rerio
RENAVRLNEKNLLKILTRLLEVSDPQVIAVAAHDVGEYVR
>vac_7_X.tropicalis
RENAARLNEKNLLKILTKLLEVSDPQVLAVAAHDIGEYVR
>vac_7_S.pombe
HQNAKRLNEDNLLKKLFHIVQYNENTSLAVACHDLGAYIR
>vac_7_Y.lipolytica
QENVGQFKDDDVLKMLAGIVKSATPTVQAVACSDVANVCK
>vac_7_D.melanogaster
RENAQRLNEKNLLRILVHLLETSKAIILSVACFDIGEYVR
>vac_7_T.castaneum
RENAQRLNEKNLLRILIHLLETSKPLVLSVASYDIGEYVR
>vac_7_C.neoformans
KENGIRIGQEEAVKRLVELITTSKPLVLAVATHDIGQFVK
>vac_7_O.sativa
RENINNFEENDILRVLMTIIDTSSTTALAVACYDLSQFLQ
>vac_7_A.thaliana
RENVTCFEENDILRVLLTILDTSSPRSLAVACFDISQFIQ

```
>vac_7_D.discoideum
KENISKFEENNVIKHLHQILKTSQPLQLSIACHDLCEFVR
>vac_7_T.thermophila
KENVKKFEDNDLISKLCDLLDSDIPKNVAIACYDIGEFCR
>mo_1_H.sapiens                                    210
EAVAQLAQELYSSGLLVTLIADLQFEEKKDVTQIFNNILR
>mo_1_X.laevis
ETVAQLAQELYNSGLLVTLIANLHFEGKKDVSQIFNNILR
>mo_1_S.cerevisiae
EAIDELYTAMHRADVFYELLLHFVFEARRECMLIFSICLG
>mo_1_O.sativa
EQVLQITLEICKEDVLSLFVQNLPWGVRKDLVHCWCILLR
>mo_1_O.tauri
TQSRALVKAASKVSLATQLVEHLRFETRKDAATVFNCLIR
>mo_1_Y.lipolytica
ALVAALASEMHQTELFTQLVTSLRFASRKDVVLIFNTLLR
>mo_1_N.crassa
EHQYQLVTGMIEEDLLYLLAINLYFDSRKDTQVIFSYVFR
>mo_1_A.thaliana
EACAQLTQEFFKEDTLRLLITCLPLETRKDATQVVANLQR
>mo_1_S.pombe
DLVSDLSFQIYQSNLPFLLVRYLPFESKKDTGLIFSALLR
>mo_1_C.neoformans
DAVAQVANEVYAQDLLSLMVVHLGFEARKDVCNIYGVLLR
>mo_1_D.melanogaster
YVVAQLSQELYNSNLLLLLIQNLHFEGKKHVALIFNNLLR
>mo_1_A.mellifera
IVVAQLAQELYNSNLLLLLVQNLSFEGKKDVAQVFNNILR
>mo_1_T.castaneum
IIVAQLAQELYNSNLLLLLIQNLNFEGKKDVAQVFNNILR
>mo_1_D.rerio
ETVAQLAQELYNSSLLISLVENLQFEGKKDVCQIFNNILR
>mo_1_C.elegans
EQVTQLAQEVYNANVLPMLIKHLHFECKKDVASVFNNLLR
>mo_2_H.sapiens                                    430
GTRSPTVEYISAHPHILFMLLKGYPQIALRCGIMLRECIR
>mo_2_X.laevis
GTRSPTVEYISSHQHILFILLKGYPQVALHCGIMLRECVR
>mo_2_S.cerevisiae
DNKFVTVDYLVSQPKTISLMLRTAQDIFLTVGNMIIECIK
>mo_2_O.sativa
DESYCCVKYIENHLELLDFLVGCYLDVALNCGNMLRECIK
>mo_2_O.tauri
DGQEVVVEELSDSPELLEVIATGYTDAALTYGAILRDLCR
>mo_2_Y.lipolytica
GDRSPTVDYLVQHPRIFEILILSYHDSALTAGEILRDCNK
>mo_2_N.crassa
EPLALAYVVERRPQVLIELCKGYDKESAQHAGTVLKELIK
>mo_2_A.thaliana
NSRLIASDYLEANIDLMDVLIEGDTDMALHYGAMFRECIR
>mo_2_S.pombe
ASRYPTVDYMLAHPQIFPVLVSYYQEVAFTAGSILRECSR
>mo_2_C.neoformans
GSRSPTVDTIATRPDIIFNTLKGYQDIALNTGMILKEMLR
>mo_2_D.melanogaster
GTRSPTVEYICTKPEILFTLMAGYPEIALNSGTMLRECAR
>mo_2_A.mellifera
GTRSPTVEYICTKPEILFTLMSGYQDIALNCGTMLRECAR
>mo_2_T.castaneum
GTRSPTVEYICTKPEILFTLMSGYQEIALNCGTMLRECAR
>mo_2_D.rerio
GTRSPTVEYFCSHQEVLFILLKGYPQVALNCGIMLRECIR
```

>mo_2_C.elegans
GTRSPTVEYLAARPEILITLLLGYPDIALTCGSMLREAVR
>mo_3_H.sapiens 83
IRHEPLAKIILFSNQFRDFFKYVEDIASDAFATFKDLLTR
>mo_3_X.laevis
VRHEPLAKVILYSEQFGDFFKYVEDIASDAFATFKDLLTR
>mo_3_S.cerevisiae
IKYEQLCRIILKDPQLWKFFEFAKEISTESLQILSAAFTA
>mo_3_O.sativa
IKYPTLTKYILDSSSFELFFEYVEDIASDALNTFKDLLTK
>mo_3_O.tauri
CRNQTLVRKILYAESFWKLFEYMQEIASDAMATFREALTR
>mo_3_Y.lipolytica
NKWEQLSKIIIWSPQLWKFFEYVDQNATDAFGSLSDIVTV
>mo_3_N.crassa
DEPGSSARGVGGNGIFWSFFDWIDEVAADAFTTFRELLTK
>mo_3_A.thaliana
IRHQIVAKYVLESDHVKKFFDYIQDIAADAAATFKELLTR
>mo_3_S.pombe
SRHEALNEVLLNSRDFWTFFSLIQDMASDAFSTFKSILLN
>mo_3_C.neoformans
LRYEPLARILLYSDQFYTFPSYIEGISCDALANMKETLTR
>mo_3_D.melanogaster
ARYEALAKIMLHSDEFFKFFRYVEDIASDAFSTFKELLTR
>mo_3_A.mellifera
ARYEALAKIMIYSDDFYNFFRYVEDIASDAFSTFKELLTR
>mo_3_T.castaneum
ARYEALAKIMLYSDDFYNFFRYVEDIASDAFSTFKELLTR
>mo_3_D.rerio
IRHEPLAKIVLHSEHFKDFFSYVEDIASDAFATFKDLLTR
>mo_3_C.elegans
VRHEHLARIVLYSEYFQRFFVFVQDIATDAFSTFKDLMTK
>mo_4_H.sapiens no match
HKVLVADFLEQNYDTIFEDYEKLLYVTKRQSLKLLGELIL
>mo_4_X.laevis
HKLMVAEFLEQNYDRIFNDYEKLLYVTKRQSLKLLGELIL
>mo_4_S.cerevisiae
LVSKEFFSNEINIIRFIKCINKLMYVTKRQSTKLLASLIV
>mo_4_O.sativa
HETVVAEFLSSHYEQFFELYTRLLYVTRRQSVKFLSEFLL
>mo_4_O.tauri
HKDIAAEFLMANYDRFFKEYTELLYVTRRQSLKLLGELLL
>mo_4_Y.lipolytica
HQQVAGEFLAANKEKFIANINKLMYVTRRQSLKLMGQLIR
>mo_4_N.crassa
RHKDLVPHYLQNFDLFFSKYNSILYVTKRQSIKLLGEILL
>mo_4_A.thaliana
HKSTVAEFLTKNEDWFFADYNSKLYITRRQAIKLLGDILL
>mo_4_S.pombe
HKSQVAEFISYHFDEFFKQYTVLLYVTKRQSLKLLGEILL
>mo_4_C.neoformans
RHKPMVAQYIENYDRFFNMYNTLIYVTKRQSLKLLGEILL
>mo_4_D.melanogaster
RHKLLCAEFLDYDKFFSQHYQRLLYVTRRQSLKLLGELLL
>mo_4_A.mellifera
RHKILSAEFLENYDKVFSHYQRLLYVTRRQSLKLLGELLL
>mo_4_T.castaneum
RHKILCADFLENYDKVFSHYQRLLYVTRRQSLKLLGELLL
>mo_4_D.rerio
RHKALVAEFLENYDAVFDNYEKLLYVTKRQSLKLLGELLL
>mo_4_C.elegans
KHKNMCAEYLDNYDRFFGQYSALTYVTRRQSLKLLGELLL

```
>mo_5_H.sapiens                                  no match
AIMTKYISKPENLKLMMNLLRDKSPNIQFEAFHVFKVFVA
>mo_5_X.laevis
SIMTKYISKPENLKLMMNLLRDKSPNIQFEAFHVFKVFVA
>mo_5_S.cerevisiae
ALMNIYINSPENLKLIMTLMTDKSKNLQLEAFNVFKVMVA
>mo_5_O.sativa
RIMKRYITEVRFLNIMITLLKDSSKNIRICAFHVFKVFVA
>mo_5_O.tauri
QVMLKYVAEVENMCFMMNLLRDESKSIQFEAFHIFKVFVA
>mo_5_Y.lipolytica
PFMTTYVNEVENLKLIMMLLKDKSKNIVIEAFNIFKLFAA
>mo_5_N.crassa
NVMTAYVDRGEHLKICMNLLRDDRKMVQYEGFHVFKVFVA
>mo_5_A.thaliana
AVMTKYVSSRDNLRILMNLLRESSKSIQIEAFHVFKLFAA
>mo_5_S.pombe
SVMTRYISSAENLKLMMILLRDKSKNIQFEAFHVFKLFVA
>mo_5_C.neoformans
NIMTRYIASEANLKMMMNFLRDKSRNIQFEAFHVFKVFVA
>mo_5_D.melanogaster
TVMTRYISEPENLKLMMNMLKEKSRNIQFEAFHVFKVFVA
>mo_5_A.mellifera
TVMTRYISNPDNLKLMMNMLKEKSRNIQFEAFHVFKVFVA
>mo_5_T.castaneum
TVMTRYISNPDNLKLMMNMLKERSRNIQFEAFHVFKVFVA
>mo_5_D.rerio
TVMTRYISKPENLKLMMNLLRDKSPNIQFEAFHVFKVFVA
>mo_5_C.elegans
STMNKYITSPENLKTVMELLRDKRRNIQYEAFHVFKIFVA
>dia_1_M.musculus                                12
NNPVSWVQGAEGLASLLDILKRLHSRNQHEIIRCLKAFMN
>dia_1_X.tropicalis
NNPVSWVQGAEGLSCLLEILKRIQVRSEHEIIRCLKAFMN
>dia_1_M.musculus_2
SHPVSWVNGYEGLGVLLDVLEKLLKKNQYKVIQCLKAFMN
>dia_1_M.musculus_3
SHPVSWVNGYEGLGVLLDVLEKLLKKNQYKVIQCLKAFMN
>dia_1_D.rerio
SNPVSWVNGHEGLGLLLDALERLLKKNQHKLIQCLKAFMN
>dia_1_A.mellifera
NNPLSWVQGTKGLKQVLATLNECYERIQYECIRCLKAIMN
>dia_1_T.castaneum
NNPLSWVRGSQGMRSIFKILDLAINKIQIECLRCVEKYMN
>dia_1_D.melanogaster
SNPISWIKGVAGIGTIEKLLARSKEKIEFEAIRCLKAIMN
>dia_1_U.maydis
TCAIGWIQEAKGTPVLASFLSGLHLSLEYEVLKAFRSLFN
>dia_1_N.crassa
TQQIGWVKDCQGQIALTNVLLKMNLDREYDIVKCLKALMN
>dia_1_Y.lipolytica
TEPVDWVRDAQGQVALAAWLQQINLDREYDIVRCLKALLN
>dia_1_D.discoideum
NQPLKWLKSLDGVSLLIKVLILNEIYKIAQCLHSLKLIMN
>dia_1_C.elegans
GQGVSFLNGADLICCLYSLVLKRLIDFLQEIVRCVRTLIN
>dia_1_S.pombe
TQSDRWISELQGLRALHNLLTYFNQPQQAEVPRCMLTLLK
>dia_1_S.cerevisiae
TEQLDWVDDHQGHIAMANVLMNSILEKENSFFKCFRVLSM
>dia_2_M.musculus                                130
-NNKFGIKTMLETEEGILLLVRAMPNMMIDAAKLLSALCI
```

```
>dia_2_X.tropicalis
-NNNFGIKAMLGTEDGILLLARAVPAMMIDSLKLLSALCI
>dia_2_M.musculus_2
-NNKFGLQRILGDERSLLLLARAIQNMMTEIVKILSAICI
>dia_2_M.musculus_3
-NNKFGLQRILGDERSLLLLARAIQNMMTEIVKILSAICI
>dia_2_D.rerio
-NNKYGLQRILGDERSLLLLARAITNMMTEIVKILSAVCI
>dia_2_A.mellifera
-NNTVGIKEMLAHQEALTIVARSLPSVMSEAVKLLGAVCL
>dia_2_T.castaneum
-NNTEGLKNFLQYDKGHEIVAKCLSQVMIQALKILAALCF
>dia_2_D.melanogaster
-NNTWGLNVVLDQHSVVLLLAQSLPQTMCEALKLLASFCI
>dia_2_U.maydis
-NSKPGANDALAQPKCISGITHSMLTTRKQAADILLFLCH
>dia_2_N.crassa
-NNKFGADDALAHQQVIVALATSLLTTRKLVSEVLTFLCH
>dia_2_Y.lipolytica
-NLRDGADHAVRTSKCVAPIVRSLLSTRKLVTDVLTFLAH
>dia_2_D.discoideum
-NTKFGLESVIKQPTNIHSISLVMLKTRIMVIELLAALCV
>dia_2_C.elegans
-NTHVGLVLVLRNSPVYSLLIQTLRAIRVDVVRTCYTLIF
>dia_2_S.pombe
-KKKPTLVT--SNSYIFQAITVTLLLPRKVAADLLTWVLS
>dia_2_S.cerevisiae
-MLSQGLYEFSTHRLMTDTVAEGLLATRKMATEIFVCMLE
>dia_3_M.musculus                               no match
LEAMTERAEMDEVERFQPLLDGLKIALKVGCLQLINALIT
>dia_3_X.tropicalis
LEALTERAEMEEMERFKPLLDGLNVALKVSCMQLINAVIS
>dia_3_M.musculus_2
LGGITAAAELNNRERFSPIVEGLELHLQVACMQFINALVT
>dia_3_M.musculus_3
LGGITAAAELNNRERFSPIVEGLELHLQVACMQFINALVT
>dia_3_D.rerio
LAAMTIAAERNNKERFAPIVEGLEQQLQVACMQLINALVT
>dia_3_A.mellifera
LDAITMNGEFKGRERFLPIVQGLMENLRVECLQLINSIIS
>dia_3_T.castaneum
LAAVTKVADAKDMPRFMPIVNGITADLQSMCFQFINALLS
>dia_3_D.melanogaster
LRAITTIAATSASERFRPIVDALFRDLACHSLIFINTLTN
>dia_3_U.maydis
LKAFDDLKTSQGRGKMGSMVGASDNEYAINNMFLINAILN
>dia_3_N.crassa
IEAMDVVKAQQGRGKMGSLVGASEMEYAVATLILVNMLVD
>dia_3_Y.lipolytica
LAGLSQLKQHLGRGKFGSLVGASDMEYSLATMFLINILIQ
>dia_3_D.discoideum
LSAMDNYREVKEKKPFIHLFQGLKGSLQATTFALINTLIS
>dia_3_C.elegans
FMELSLIAKAEPVSRFRPLISCIDPKQGMYVLLMINMMIN
>dia_3_S.pombe
AEYEKEVPLFFRTPPSSPARNSASLEYCTSTMEFINQLIV
>dia_3_S.cerevisiae
LTSLDKKFRIGGRGKMGSLVGASDLEYCQWTMVFINHLCS
>rcd_1_M.musculus                               320
PDLAPMLWHSFLLQEIVNIYPSIQSNRVCNALALLQCVAS
>rcd_1_T.thermophila
NNLAPLLWHSNLIDDIVSVYSQLVSTQVCSVLGLLQCLAL
```

>rcd_1_S.cerevisiae
DDLAVVLWSSFLLNEIISVYPMLLSNRVCNALVLLQCVAS
>rcd_1_O.sativa
PDLAPLLWHSFLLQEIIVVYPVLASSRVCNALALLQCVAS
>rcd_1_C.elegans
PDLPIWLWHSFLLQEVVAIYPAIQSNRVCNALALMQCVAS
>rcd_1_A.mellifera
PDLAPMLWHSFLLQEIINIYPAIQSNRVCNALALLQCVAS
>rcd_1_S.purpuratus
PDLAPMLWHSFLLQEIVNIYPYIQSNRVCNALALLQCIAS
>rcd_1_D.rerio
TDLAPMLWHSCLLQEIVNIYPSIQSNRVCNALALLQCVAS
>rcd_1_X.tropicalis
PDLAPMLWHSFLLQEIVNIYPSIQSNRVCNALALLQCVAS
>rcd_1_V.carteri
PELAPYLWHSFLLQEIVAIYPLLASNRVCNALALLQCVAS
>rcd_1_A.thaliana
QDLAPLLWNSFLLQEIVSIYSVLQSNRVCNSLALLQCVAS
>rcd_1_U.maydis
EDLALVLWHSFLLQEIVSVYPLLASNRVCNALALLQCVAS
>rcd_1_S.pombe
EDLALILWHSYLLQEIISVYPLLTSNRVCNALALLQCIAS
>rcd_1_Y.lypolytica
EDLALILWHSFLLQEIMNVYSLLASNRVCNALALLQCVAS
>rcd_1_N.crassa
PELALILWHSFLLQEIISVYTLLASNRVCNALALLQCVAS
>rcd_2_M.musculus 48
PETRSAFLAAHFLYPFLHTVSKTFEYLRLTSLGVIGALVK
>rcd_2_T.thermophila
PETKPHFIKAHFLYPFLNTSAKLFENLRVTSLGVIGALVK
>rcd_2_S.cerevisiae
PETKHLFLQAHFLFPFLNTTSRQFEYLRLTSLGVIGALVK
>rcd_2_O.sativa
PETRSHFLKAHYLCSFLENTSKTFEYLRLTSLGVIGALVK
>rcd_2_C.elegans
RDTRGPFLHAHYLYPFLHTTKVSFEYLRLTSLGVIGALVK
>rcd_2_A.mellifera
PETRSSFLQAHFLYPFLHTVSKTFEYLRLTSLGVIGALVK
>rcd_2_S.purpuratus
PETRSAFLQAHFLYPFLHTVSKTFEYLRLTSLGVIGALVK
>rcd_2_D.rerio
VETRSAFLAAHFLYPFLHTVSKTFEYLRLTSLGVIGALVK
>rcd_2_X.tropicalis
PETRSAFLAAHFLYPFLHTVSKTFEYLRLTSLGVIGALVK
>rcd_2_V.carteri
NETRALFLQAHFLYPFLQTMSKTFEYLRLTSLGVIGALVK
>rcd_2_A.thaliana
SDTRMLFLKAHYLYPFLNTTSKSFEYLRLTSLGVIGALVK
>rcd_2_U.maydis
SETRGLFLQAHFLYPFLNTTSKTFEYLRLTSLGVIGALVK
>rcd_2_S.pombe
PETRIHFLNAHFLYPFLNTLSKSFEYLRLTSLGVIGALVK
>rcd_2_Y.lypolytica
ADTRTPFLVAHFLYPFLNTTVKSFEYLRLTSLGVIGALVK
>rcd_2_N.crassa
NETRTLFLNAHFLYPFLNTTSKSFEYLRLTSLGVIGALVK
>rcd_3_M.musculus 280
TDEQEVINFLLIIPLCLRIMESGSELSKTVATFILQKILL
>rcd_3_T.thermophila
GDDTEAITFLMIIPLCLRIMKRGQELSRTVATFIVQKILI
>rcd_3_S.cerevisiae
NDSQDVITFLLIVPLCLRIMESSSELSKTVAIFILQKILL

>rcd_3_O.sativa
AEGTEVINFLLFVPLCLHAMAVGSELSKTVATFIIEKIVL
>rcd_3_C.elegans
TQLLIVINFLLIIPLCLRIMEQGTELSKTVATFILQKILL
>rcd_3_A.mellifera
TDEQEVITFLLIIPLCLRIMESGSELSKTVATFILQKILL
>rcd_3_S.purpuratus
TDEQEVINFLLIIPLCLRIMESGSELSKTVATFILQKILL
>rcd_3_D.rerio
TDEQEVINFLLIIPLCLRIMESGSELSKTVATFILQKILL
>rcd_3_X.tropicalis
TDEQEVINFLLIIPLCLRIMESGSELSKTVATFILQKILL
>rcd_3_V.carteri
VDDTDVINFLLIIPLCLRTMEIGTELSKTVATFIVQKILL
>rcd_3_A.thaliana
VDDTEVISFLLIIPLCLRTMEMGSELSKTVATFIVQKILL
>rcd_3_U.maydis
NDNSDVITFLLIIPLCLRIMETGSELSKTVAIFIVQKILL
>rcd_3_S.pombe
NDSPEVINFLLIIPLCLRIMENGSELSKTVAIFIVQKFLC
>rcd_3_Y.lypolytica
NDSPEVISFLLIIPLCLRIMESGTELSKTVAIFIVQKILM
>rcd_3_N.crassa
NDSTEVINFLLIIPLCLRIMETGSELSKTVAIFIVQKILL
>rcd_4_M.musculus 320
DDTGLAYICQTVAMILGKMVLQLSARLLKHVVRCYLRLSD
>rcd_4_T.thermophila
DDNGLNYICQTVSTVLQSMIEDLDQRLLRHIIKCYQRLSE
>rcd_4_S.cerevisiae
DDVGLQYICATVTNVLKDMVEHLPGRLLKHIIRCYLRLSD
>rcd_4_O.sativa
DDAGLGYICATVGTALAGMVTSMSPRLLKHIIHCYLRITD
>rcd_4_C.elegans
DDTGLLYICQTVVLILGKMVMKLSVRLLKHVVRCYSRLSD
>rcd_4_A.mellifera
DDSGLSYICQTVAMILGKMVLSLSARLLKHVVRCYLRLSD
>rcd_4_S.purpuratus
DETGLSYICQTVAMILGKMVIALSARLLKHVVRCYLRLSD
>rcd_4_D.rerio
DDTGLAYICQTVAMILGKMVLQLSARLLKHVVRCYLRLSD
>rcd_4_X.tropicalis
DDTGLAYICQTVAMILGKMVLQLSARLLKHVVRCYLRLSD
>rcd_4_V.carteri
DDVGLNYICATVGAVLGNMVVAQSQRLLKHIIRCYLRLSD
>rcd_4_A.thaliana
DDVGMDYICTTVGRVLGNMVQSLSPRLLKHITRCYLRLSD
>rcd_4_U.maydis
DDMGLAYICQTVGTVLSNMVSQIAVRLLKHVVRCYLRLSD
>rcd_4_S.pombe
DDVGLQYICQTVASVLNNMVMQLAFRLLKHVIRCYLRLSD
>rcd_4_Y.lypolytica
DDMGLNYVCQTVSQVLANMVQQLASRLLKHVVRCYLRLSD
>rcd_4_N.crassa
DDNGLNYICATVGTVLSNMVAQLTARLLKHVVRCFLRLSD
>fhod_1_H.sapiens 9.2
DLVPEFVHSE-GLSCLIRVGAAADHNYQSYILRALGQLML
>fhod_1_T.guttata
DLVPEFVNLE-GLTCLIKVGAEADQNYQNYILRALSQIML
>fhod_1_D.rerio
DLVPEFVISE-GLTCFIKVGAEADHNYQNYILRALSQIML
>fhod_1_N.vectensis
DLVHEFVNSD-GLNALITVGQDADQNYQNYILRAIGQIML

```
>fhod_1_A.pisum
DLVHEFIQND-GLRCLIKLGSDVDQNYQNYILRALGQVML
>fhod_1_D.melanogaster
DLVHAFVALG-GLNCLVRVGNCADQNYQNYILRALGQVML
>fhod_1_C.elegans
DLVHEFVQNQ-GLDCMIRLGRTADQNHQNYILRALGQLML
>fhod_2_H.sapiens
VDGMLGVVAHSTIQWLYTLCASLSRLVVKTALKLLLVFVE
>fhod_2_T.guttata                                   230
VDGMQGVINHNTVQWLYTLSGSPFRLVVKTALKLLLVFVE
>fhod_2_D.rerio
VDGMNGIVNHNTVQWLYTLCGSLSRLVVKTALKLLIVFVE
>fhod_2_N.vectensis
VDGMNGVINHNTIQWLYSLTASKFRLVAKTALKLLLVFIE
>fhod_2_A.pisum
VDGMNGVIEDNTIQWLYSLLTSRFRLVVKTALKLLLVFIE
>fhod_2_D.melanogaster
VDGMNGVMKHETMQWLYSLIASNYRSVVKTALKLLLVFVE
>fhod_2_C.elegans
VDGMNGIIAHNTIQWLYELLDSPFRLVVKTALKLLLVFIE
>fhod_3_H.sapiens                                   240
IRAVNSVASTTPWANLVSILEEKDPELLVYTVTLINKTLA
>fhod_3_T.guttata
IRAVNAVDQARPWSNLMAILEQRDTELLVFTMTLINKTLA
>fhod_3_D.rerio
IQAVNIVDGKRPWTILIDILSAKDTELLIYTMTLINKTLA
>fhod_3_N.vectensis
VEAVQCVDDAQRWSEIVGILSEKDEEILIYAMTLVNKALY
>fhod_3_A.pisum
IQAVLAVHQSNLWSNVMKLLTEPDTELLVYAMTLINKTLN
>fhod_3_D.melanogaster
VSAIHAVDASQPWSNIMRLLKDYDAELVIYATSLINKTLA
>fhod_3_C.elegans
LTAIQTVDKSKDWSGLMKVLTEKDAETLVYGMTVVNKALH
```
